# Reaction of Diethyl 2‑Hydroxyazulene-1,3-dicarboxylate with Metal Acetates and Alkoxides: Metal Complex Formation and Transesterification

**DOI:** 10.1021/acsomega.5c01929

**Published:** 2025-08-25

**Authors:** Tatsuya Iwashina, Ryohei Hayami, Yohei Sato, Takuya Sagawa, Kazuki Yamamoto, Takahiro Gunji

**Affiliations:** † Department of Pure and Applied Chemistry, Faculty of Science and Technology, 26413 Tokyo University of Science, 2641 Yamazaki, Noda, Chiba 278-8510, Japan; ‡ Department of Industrial Chemistry, Faculty of Engineering, 26413 Tokyo University of Science, 6-3-1 Niijuku, Katsushika-ku, Tokyo 125-8585, Japan; § Photocatalysis International Research Center, 26413 Tokyo University of Science, 2641 Yamazaki, Noda, Chiba 278-8510, Japan; ∥ Research Group for Advanced Energy Conversion, Research Institute for Science and Technology (RIST), 26413 Tokyo University of Science, 2641 Yamazaki, Noda, Chiba 278-8510, Japan

## Abstract

Azulene-embedded heterometallacycles can be handled in air and are expected to have a glossy color derived from the azulenyl ligand. However, very few studies have clearly described the synthesis, structure, and effects of metal species. In this study, the reactions of diethyl 2-hydroxyazulene-1,3-dicarboxylate (DEHA, other abbreviation as H-L) with various metals were investigated to synthesize metal complexes (**Metals-L**). **Metals-L** were synthesized by the reaction of metal acetates (Pd, Cu, Ni, Co, and Zn acetate) with DEHA and Cs_2_CO_3_. Single-crystal X-ray structural analysis showed that **Pd-L** and **Cu-L** had mononuclear planar and binuclear structures, respectively. In the case of zinc acetate, the formation of a one-dimensional coordination polymer, [ZnCs­(OC_10_H_5_(COOC_2_H_5_)_2_)_3_]_*n*_, was confirmed. For cobalt and nickel acetates, the formation of similar coordination polymers was suggested by mass spectrometry, infrared spectrometry, and energy-dispersive X-ray spectrometry. These complexes were mixed in poly­(methyl methacrylate) (PMMA), and the PMMA composite films exhibited coloration corresponding to the color of the complexes. When DEHA was mixed with titanium tetraisopropoxide and Cs_2_CO_3_ in an alcohol (*i*PrOH, *n*BuOH, or allyl alcohol), transesterification was observed by nuclear magnetic resonance spectroscopy. This transesterification was necessary for both titanium tetraisopropoxide and Cs_2_CO_3_, and a facile transesterification reaction of DEHA was observed for the first time.

## Graphical abstract

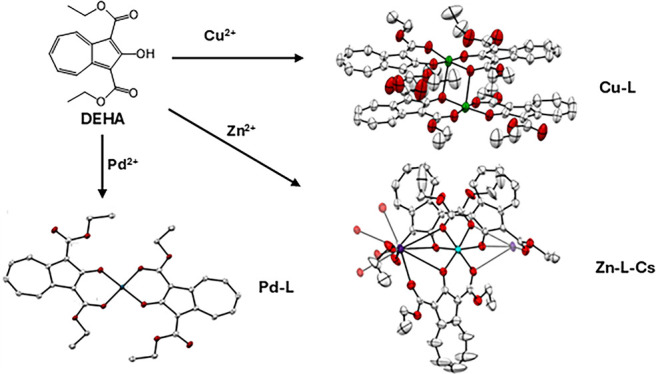

## 1 Introduction

Azulene, a structural isomer of naphthalene, is known for its unique fused five- and seven-membered rings and tunable colors.^1,2^ Azulene exhibits both electron-donating and electron-accepting abilities, resulting in a dipole moment of 1.08 D.^3^ The unique nature of azulene derivatives has attracted attention, and many researchers have studied these syntheses and properties.^4–8^

Coordination chemistry of azulene is also attracting attention as a research subject.^9^ Azulene complexes have been reported as organometallic complexes, which contain chemical bonds between the carbon atom of ligands and metal species, and metal organic complexes, which have bonds between noncarbon elements of ligands and metal species (Figure 1).^10–21^ Some organometallic complexes have been proposed as intermediates in catalytic reactions, including exchange reaction and cross-coupling reactions.^22–25^ In metal organic complexes, sensors utilizing the vivid color of azulene groups and changes in catalytic activity caused by alterations in the electron configuration of metal species due to azulene groups have been reported.^16,21,26,27^ The synthesis of azulene complexes and their application in research are increasingly intriguing and are gaining attention. However, the synthesis of these ligands, starting from tropolone as the raw material, requires more than five reaction steps. We focused on diethyl 2-hydroxyazulene-1,3-dicarboxylate (DEHA) and diethyl 2-aminoazulene-1,3-dicarboxylate (DEAA), which can be obtained through two reaction steps starting from tropolone (Scheme 1).

**Figure 1:**
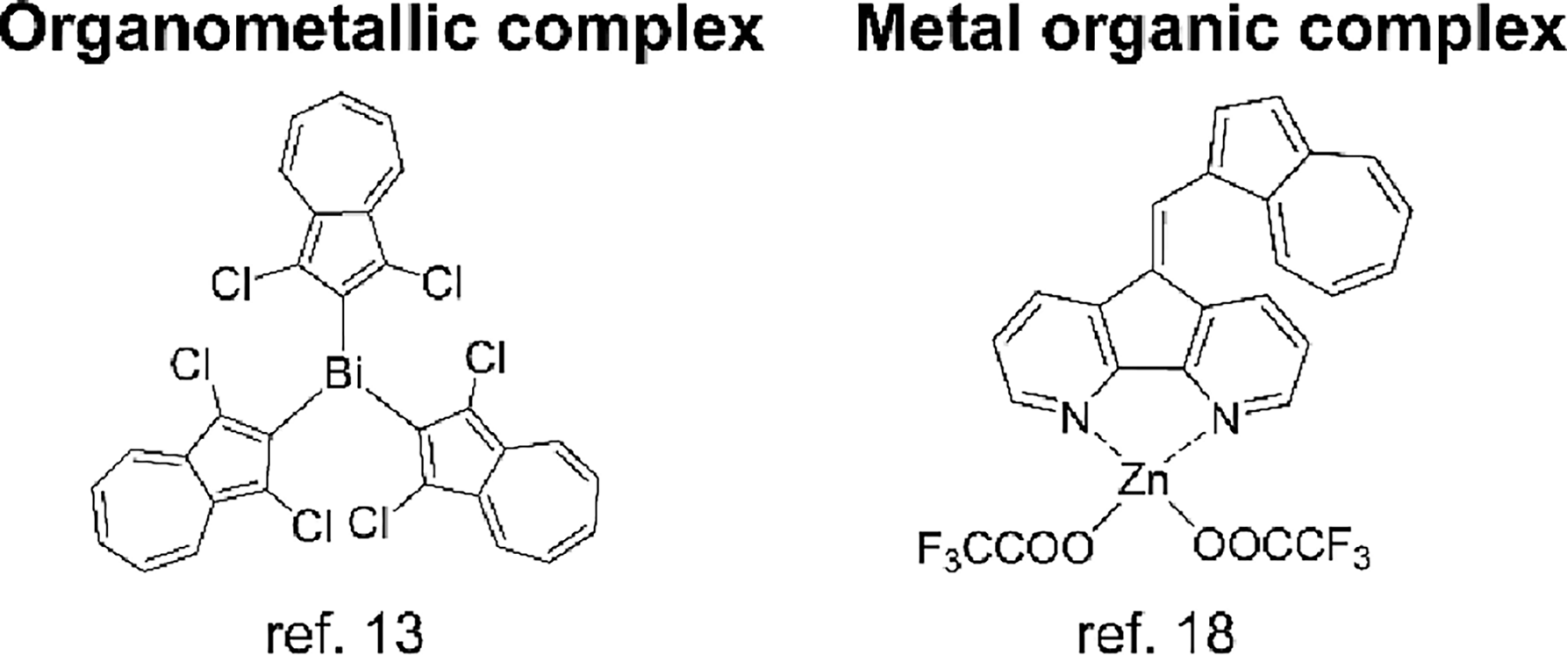
Organometallic and metal organic complexes.

**Scheme 1:**
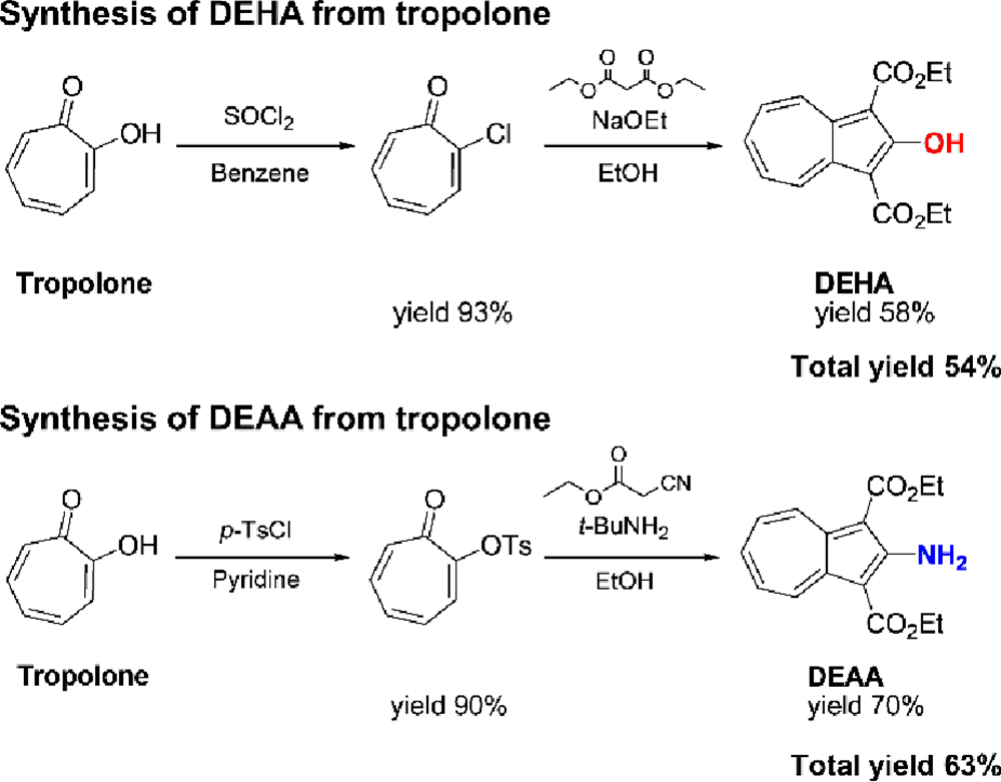
Syntheses of Diethyl 2-Hydroxyazulene-1,3-dicarboxylate (DEHA) and Diethyl 2-Aminoazulene-1,3-dicarboxylate (DEAA)

Using these azulene ligands, we investigate azulene-embedded heterometallacycles, in which the metallacycles are combined with azulene groups, and this is one of the metal organic complexes. Previously, our investigation revealed that DEAA forms a complex exclusively with a palladium ion, highlighting its high affinity for palladium and potential for adsorption applications.^28^ However, DEAA did not form complexes with other metal ions, and research on the synthesis and properties of DEAA complexes was not pursued further. In contrast, DEHA has suggested versatility, with studies by Zhang and Petoud reporting high quantum yields in potassium lanthanide complexes due to efficient triplet-state energy transfer and cation protection.^29^ Furthermore, fluorescence emission and wavelength variation, depending on metal species, have been observed. These observations highlight the potential of azulene ligands, particularly DEHA, in advancing coordination chemistry and developing functional materials. Building on this foundation, we directed our efforts toward the synthesis of metal complexes and the investigation of their structural and physical properties. Moreover, given previous reports on composite films of poly­(methyl methacrylate) (PMMA) and metal complexes,^30,31^ we became interested in developing composite films incorporating PMMA and azulene-embedded heterometallacycles.

In this study, we synthesized metal complexes (**Metals-L**) by reacting DEHA with divalent metal species in the presence of Cs_2_CO_3_ (Scheme 2) and investigated the changes in the structure and physical properties caused by the metal species. Palladium, copper, nickel, cobalt, and zinc acetates were chosen as metal sources to explore their interactions with DEHA. Next, PMMA and **Metals-L** composite films were prepared, and their dispersibility and heat resistance were evaluated. Additionally, the transesterification of DEHA was examined by reacting it with titanium tetraisopropoxide (Ti­(O*i*Pr)_4_) and Cs_2_CO_3_ in alcohols (Scheme 2).

**Scheme 2:**
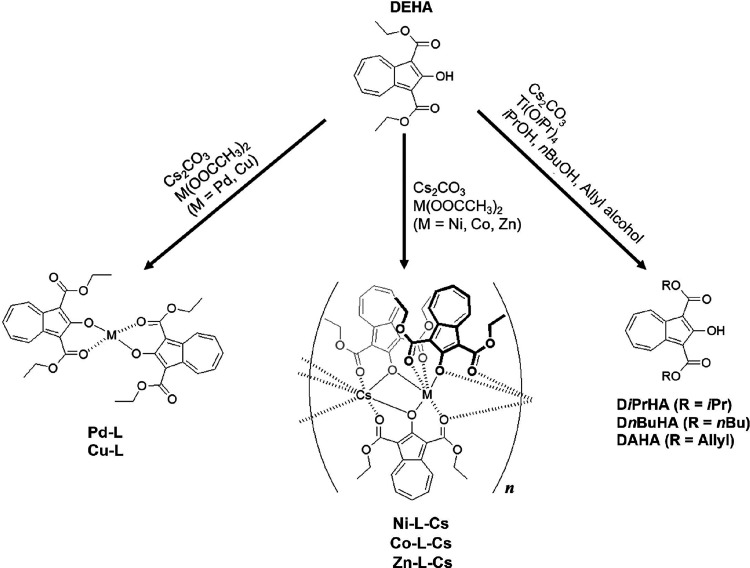
Reaction of DEHA with Metal Acetates (Formation of Azulene-Embedded Heterometallacycles, **Metals-L**) and Metal Alkoxides in Alcohols (Transesterification)

## 2 Experimental Section

### 2.1 Measurements

The ^1^H and ^13^C­{^1^H} nuclear magnetic resonance (NMR) spectra were measured using a JNM-ECZ400S spectrometer (JEOL, Japan; ^1^H at 399 MHz; ^13^C­{^1^H} at 100 MHz) at 22 ± 5 °C. The chemical shifts of ^1^H and ^13^C­{^1^H} NMR spectra were calibrated using the residual protons and ^13^C atoms in deuterated solvents,^32^ respectively. Fourier-transform infrared (FTIR) spectroscopy was performed using an FT/IR-6100 spectrometer (JASCO, Japan) with an attenuated total reflectance (ZnSe prism, JASCO ATR PRO 0450-S). Molecular weight was measured using a JMS-T100CS AccuTOF CS instrument (JEOL, Japan). Ionization was performed by electrospray ionization (ESI) or atmospheric-pressure chemical ionization (APCI). Elemental analyses were performed using a 2400II elemental analyzer (PerkinElmer, USA). Thermogravimetric-differential thermal analysis (TG-DTA) was performed using a TG-DTA 2000SE instrument (Netzch Japan, Japan). The samples were heated to 1000 °C at a rate of 10 °C/min under an air flow. The decomposition temperatures were visually recorded using an SMP3 melting point apparatus (Stuart, UK). Molecular weight was determined by gel permeation chromatography (GPC) performed using an LC-20AD high-performance liquid chromatography prominence liquid chromatograph (Shimadzu, Japan) attached to a PLgel 5 μm Mixed-D column. Ultraviolet–visible (UV–Vis) spectroscopy was performed using a V-670 UV–vis–NIR spectrophotometer (JASCO, Japan). Fluorescence spectra were performed using an FP-8600 instrument (JASCO, Japan). Energy-dispersive X-ray spectrometry (EDX) was performed by using an SU9000 field-emission scanning electron microscope (Hitachi High-Technologies, Japan). The samples for EDX were gold-deposited. Scanning electron microscopy (SEM) was performed using a JCM-6000 (JEOL, Japan) instrument at an accelerating voltage of 15 kV after gold vapor deposition onto the sample. Single crystal X-ray structural analysis was performed using an XtaLAB Synergy-S instrument (RIGAKU, Japan) with a monochromatic Mo Kα radiation source (0.7107 Å). The deposition numbers were CCDC 2406168 for **Cu-L**, 2406169 for **Pd-L**, and 2406173 for **Zn-L-Cs**. These data can be obtained free of charge at http://www.ccdc.cam.ac.uk/conts/retrieving.html or from the Cambridge Crystallographic Data Centre, 12 Union Road, Cambridge CB2 1EZ, UK; Fax: (+44) 1223–336–033; E-mail: deposit@ccdc.cam.ac.uk.

### 2.2 Reagents

Hexane, tetrahydrofuran (THF), and sodium hydroxide were purchased from Kanto Chemical Co. (Tokyo, Japan). Cesium carbonate (Cs_2_CO_3_), copper­(II) acetate (Cu­(OAc)_2_), dichloromethane (CH_2_Cl_2_), palladium­(II) acetate (Pd­(OAc)_2_), cobalt­(II) acetate tetrahydrate (Co­(OAc)_2_·4H_2_O), nickel­(II) acetate tetrahydrate (Ni­(OAc)_2_·4H_2_O), zinc acetate (Zn­(OAc)_2_), 6 mol/L hydrochloric acid, and sulfuric acid (H_2_SO_4_) were purchased from FUJIFILM Wako Pure Chemical Corporation (Tokyo, Japan). Ti­(O*i*Pr)_4_ was purchased from Tokyo Chemical Industry Co., Ltd. (Tokyo, Japan). PMMA (*M*_w_ = 999,600 g mol^–1^) was purchased from Sigma-Aldrich Japan (Tokyo, Japan). Chloroform (CHCl_3_) and methanol were purchased from Godo Co., Ltd. (Tokyo, Japan). All solvents were dried over molecular sieves. A 100 mL vial was purchased from AGC Techno Glass Co., Ltd. (Shizuoka, Japan), dried in a drying oven at 110 °C for more than 2 h, and then cooled in a desiccator before use. Diethyl 2-hydroxyazulene-1,3-dicarboxylate (DEHA) and diethyl 2-aminoazulene-1,3-dicarboxylate (DEAA) were synthesized according to the literature,^28,33,34^ and the synthetic method is described in the Supporting Information.

### 2.3 Synthesis of **Pd-L**

In a dried 100 mL vial, DEHA (0.10 g, 0.35 mmol) and THF (25 mL) were added, and then, Pd­(OAc)_2_ (0.039 g, 0.17 mmol) was added and dissolved with stirring. Cs_2_CO_3_ (0.17 g, 0.52 mmol) was then added, and the mixture was stirred at 22 ± 5 °C for 30 min. A yellow precipitate was obtained, which was filtered and washed with THF. The precipitate was dissolved in CHCl_3_ and washed with water. The organic layer was evaporated, and **Pd-L** was obtained as a yellow powder (yield 19%). Single crystals of **Pd-L** were prepared by slowly evaporating a CH_2_Cl_2_ solution of **Pd-L** in air.

#### 2.3.1 Pd-L

Decomp. 196 °C; anal. for C_32_H_30_O_10_Pd (%): C, 56.47; H, 4.41; found (%): C, 56.84; H, 4.52. APCI-HRMS (*m*/*z*): calcd for [Pd­(OC_10_H_5_(COOC_2_H_5_)_2_)_2_+Na]^+^ 703.07841; found: 703.07794.

^1^H NMR (399 MHz, CD_2_Cl_2_, ppm) 1.39 (t, *J* = 7.0 Hz, 6.0H), 1.56 (t, *J* = 7.0 Hz, 6.0H), 4.35 (q, *J* = 7.2 Hz, 4.0H), 4.63 (q, *J* = 7.2 Hz, 4.0H), 7.46 (t, *J* = 9.2 Hz, 2.0H), 7.57 (t, *J* = 9.2 Hz, 2.0H), 7.59 (t, *J* = 9.2 Hz, 2.0H), 8.85 (d, *J* = 10.0 Hz, 2.0H), 9.26 (d, *J* = 10.0 Hz, 2.0H). ^13^C NMR (100 MHz, CD_2_Cl_2_, ppm): 14.8, 14.6, 60.0, 60.3, 64.1, 64.2, 100.8, 106.5, 131.3, 132.5, 133.7, 133.8, 134.3, 134.4, 145.0, 150.0, 165.7, 169.8, 181.3.

### 2.4 Synthesis of **Cu-L**

In a dried 100 mL vial, DEHA (0.10 g, 0.35 mmol), EtOH (30 mL), and Cu­(OAc)_2_ (0.032 g, 0.17 mmol) were added and dissolved with stirring. Cs_2_CO_3_ (0.17 g, 0.52 mmol) was added, and a green precipitate was quickly formed. The mixture was then stirred at 80 °C for 24 h. The resulting solid was filtered and washed with EtOH and then with H_2_O. **Cu-L** was obtained as a green solid (yield 46%). Single crystals of black block crystals were obtained by the liquid–liquid diffusion method from **Cu-L** by using a THF solution covered with hexane.

#### 2.4.1 Cu-L

Decomp. 241 °C; ESI-HRMS (*m*/*z*): calcd for [Cu­(OC_10_H_5_(COOC_2_H_5_)_2_)_2_+Na]^+^ 660.10279; found: 660.10327, calcd for [Cu_2_(OC_10_H_5_(COOC_2_H_5_)_2_)_4_+Na]^+^ 1297.21677; found: 1297.21934.

### 2.5 Synthesis of **Ni-L-Cs**

In a dried 100 mL vial, DEHA (0.10 g, 0.35 mmol), EtOH (30 mL), and Ni­(OAc)_2_·4H_2_O (0.031 g, 0.17 mmol) were added and dissolved with stirring. Cs_2_CO_3_ (0.17 g, 0.52 mmol) was added and stirred at 80 °C. A yellow precipitate was formed after 2 h, and the mixture was stirred at 80 °C for 22 h. The precipitate was filtered and washed with EtOH, followed by washing with water. **Ni-L-Cs** was obtained as a yellow solid (yield 44%).

#### 2.5.1 Ni-L-Cs

Decomp. 267 °C; anal. for C_48_H_45_O_15_NiCs (%): C, 54.73; H, 4.31; found (%): C, 54.53; H, 4.18. ESI-HRMS (*m*/*z*): calcd for [Ni­(OC_10_H_5_(COOC_2_H_5_)_2_)_3_]^−^ 919.21119; found: 919.21034.

### 2.6 Synthesis of **Co-L-Cs**

In a dried 100 mL vial, DEHA (0.10 g, 0.35 mmol), EtOH (30 mL), and Co­(OAc)_2_·4H_2_O (0.043 g, 0.17 mmol) were added and dissolved with stirring. Cs_2_CO_3_ (0.17 g, 0.52 mmol) was added, and the mixture was stirred at 80 °C. An orange precipitate was formed after 2 h, and the mixture was stirred at 80 °C for 22 h. The precipitate was filtered and washed with EtOH, followed by washing with water. The precipitate was extracted with CH_2_Cl_2_, and the mixture was evaporated. **Co-L-Cs** was obtained as an orange solid (yield 41%).

#### 2.6.1 Co-L-Cs

Decomp. 254 °C; anal. for C_48_H_45_O_15_CoCs (%): C, 54.71; H, 4.30; found (%): C, 54.47; H, 4.12. ESI-HRMS (*m*/*z*): calcd for [Co­(OC_10_H_5_(COOC_2_H_5_)_2_)_3_]^−^ 920.20904; found: 920.20764.

### 2.7 Synthesis of **Zn-L-Cs**

In a dried 100 mL vial, DEHA (0.10 g, 0.35 mmol), EtOH (30 mL), and Zn­(OAc)_2_ (0.032 g, 0.17 mmol) were added, and the mixture was dissolved with stirring. Cs_2_CO_3_ (0.17 g, 0.52 mmol) was added and stirred at 80 °C. A yellow precipitate was formed after 12 h, and the mixture was stirred at 80 °C for 12 h. The precipitate was filtered and washed with EtOH, followed by washing with water. The precipitate was extracted with CH_2_Cl_2_ and evaporated. **Zn-L-Cs** was obtained as an orange solid (yield 47%). When this reaction was performed in MeOH, a single crystal was obtained (Supporting Information).

#### 2.7.1 Zn-L-Cs

Decomp. 231 °C; anal. for C_48_H_45_O_15_ZnCs (%): C, 54.38; H, 4.28; found (%): C, 54.50; H, 4.12. ESI-HRMS (*m*/*z*): calcd for [Zn­(OC_10_H_5_(COOC_2_H_5_)_2_)_3_]^−^ 925.20499; found: 925.20484.

^1^H NMR (399 MHz, CD_2_Cl_2_, ppm) 1.35 (t, *J* = 7.2 Hz, 18.0H), 4.31 (q, *J* = 7.2 Hz, 12.0H), 7.22 (t, *J* = 9.6 Hz, 3.0H), 7.40 (t, *J* = 9.6 Hz, 6.0H), 8.81 (d, *J* = 9.6 Hz, 6.0H). ^13^C NMR (100 MHz, CD_2_Cl_2_, ppm): 14.8, 60.1, 104.8, 129.4, 130.5, 132.1, 146.3, 168.1, 184.5.

### 2.8 Preparation of PMMA Composite Films

PMMA (0.3 g) and DEHA or **Metal-L** (0.1, 1, 3 wt % based on PMMA) were mixed in THF (5 mL) (**Pd-L** and **Cu-L** were mixed in a mixed solvent of 3 mL of THF and 2 mL of CH_2_Cl_2_), and the solution was cast in 3 cm diameter glass Petri dishes. The Petri dishes were allowed to stand at room temperature for 1 day and then dried in an electrical oven at 80 °C for 1 day.

### 2.9 Reaction of DEHA with Ti­(O*i*Pr)_4_ and Cs_2_CO_3_ (Transesterification)

In a dried 100 mL vial, DEHA (0.10 g, 0.35 mmol) was dissolved in alcohol (30 mL, *i*PrOH or *n*BuOH), and then Ti­(O*i*Pr)_4_ (0.049 g, 0.17 mmol) was added, and the mixture was dissolved with stirring. Cs_2_CO_3_ (0.17 g, 0.52 mmol) was then added, and the mixture was stirred at 80 °C for 6 h. The solvent was removed by vacuum evaporation at 100 °C. The residue was extracted with CHCl_3_, and the CHCl_3_ was evaporated. The transesterification conversion of the crude compound was determined by ^1^H NMR spectroscopy.

### 2.10 Reaction of DEHA with HCl Aq. in *i*PrOH

In a dried 100 mL vial, DEHA (0.10 g, 0.35 mmol), *i*PrOH (30 mL), and 6 mol/L HCl (aq, 0.17 mL) were added and stirred at 80 °C for 6 h. Solvent was removed by evaporation, and a yellow powder was obtained. The transesterification conversion of the crude compound was determined by ^1^H NMR spectroscopy.

### 2.11 Reaction of DEHA with H_2_SO_4_ in *i*PrOH

In a dried 100 mL vial, DEHA (0.10 g, 0.35 mmol), *i*PrOH (30 mL), and 95% H_2_SO_4_ (0.11 g) were added, and the mixture was stirred at 80 °C for 6 h. The reaction solution was transferred to a separatory funnel, washed with water, and extracted with CH_2_Cl_2_. Solvent was removed by evaporation, and a yellow powder was obtained. The transesterification conversion of the crude compound was determined by ^1^H NMR spectroscopy.

### 2.12 Reaction of DEHA with NaOH in *i*PrOH (formation of **Na-L**)

In a dried 100 mL vial, DEHA (0.10 g, 0.35 mmol), *i*PrOH (30 mL), and sodium hydroxide (0.042 g, 1.05 mmol) were added and stirred at 80 °C for 6 h. The solvent was removed by evaporation, and an orange powder was obtained. Although no transesterification of DEHA occurred, **Na-L** was obtained.

#### 2.12.1 Na-L

^1^H NMR (399 MHz, DMSO-*d*_6_, ppm) 1.30 (t, *J* = 7.0 Hz, 6.0H), 4.23 (q, *J* = 7.0 Hz, 4.0H), 6.89 (t, *J* = 10.0 Hz, 1.0H), 7.19 (t, *J* = 10.0 Hz, 2.0H), 8.53 (d, *J* = 10.0 Hz, 2.0H).

### 2.13 Reaction of DEHA with Al­(O*i*Pr)_3_ and Cs_2_CO_3_ in *i*PrOH (Formation of **Al-L-Cs** and Little Transesterification)

In a dried 100 mL vial, DEHA (0.10 g, 0.35 mmol), *i*PrOH (30 mL), and Al­(O*i*Pr)_3_ (0.024 g, 0.17 mmol) were added, and the mixture was dissolved with stirring. Cs_2_CO_3_ (0.17 g, 0.52 mmol) was added, and the mixture was stirred at 80 °C for 6 h. The yellow precipitate and filtrate were separated by filtration. The yield of this yellow precipitate was 0.09 g. The precipitate was identified as an **Al-L-Cs** tube using ^1^H NMR spectroscopy and EDX. The filtrate was then extracted with CH_2_Cl_2_ and washed with water. After evaporation, a crude product was obtained. The transesterification conversion of the crude product was determined by ^1^H NMR spectroscopy.

#### 2.13.1 Al-L-Cs

^1^H NMR (399 MHz, THF-*d*_8_, ppm) 1.34 (t, *J* = 7.2 Hz, 6.0H), 4.27 (q, *J* = 7.2 Hz, 4.0H), 6.82 (t, *J* = 10.2 Hz, 1.0H), 7.10 (t, *J* = 10.2 Hz, 2.0H), 8.59 (d, *J* = 10.2 Hz, 2.0H).

### 2.14 Reaction of DEHA with Ti­(O*i*Pr)_4_ and Cs_2_CO_3_ in Allyl Alcohol (Transesterification)

In a dried 100 mL vial, DEHA (0.050 g, 0.18 mmol) was dissolved in allyl alcohol (15 mL), and then, Ti­(O*i*Pr)_4_ (0.025 g, 0.085 mmol) was added and dissolved with stirring. Cs_2_CO_3_ (0.085 g, 0.26 mmol) was then added, and the mixture was stirred at 80 °C for 6 h. The reaction solution was allowed to cool to room temperature, washed with water, and extracted with dichloromethane. After evaporation, the solution was again dissolved in dichloromethane and passed through silica gel. After evaporation, yellow powdered DAHA was obtained in a 47% yield.

#### 2.14.1 DAHA

ESI-HRMS (*m*/*z*): calcd for [M + Na]^+^ 335.08954; found: 335.08937.

^1^H NMR (399 MHz, CDCl_3_, ppm) 4.97 (d, *J* = 5.6 Hz, 4.0H), 5.34 (dd, *J* = 10.6, 1.2 Hz, 2.0H), 5.52 (dd, *J* = 17.0, 1.2 Hz, 2.0H), 6.08–6.18 (m, 2.0H), 7.72 (t, *J* = 6.0 Hz, 3.0H), 9.40 (d, *J* = 6.0 Hz, 2.0H), 11.7 (s, 1.0H). ^13^C NMR (100 MHz, CDCl_3_, ppm): 66.2, 101.2, 118.6, 132.4, 132.9, 135.1, 136.5, 143.7, 166.5, 172.5.

### 2.15 Reaction of DEAA with Ti­(O*i*Pr)_4_ and Cs_2_CO_3_ (Transesterification)

In a dried 100 mL vial, DEAA (0.050 g, 0.17 mmol) was dissolved in alcohol (15 mL, *i*PrOH or allyl alcohol), and then Ti­(O*i*Pr)_4_ (0.025 g, 0.087 mmol) was added and dissolved with stirring. Cs_2_CO_3_ (0.085 g, 0.026 mmol) was then added, and the mixture was stirred at 80 °C for 6 h. The solvent was removed by vacuum evaporation at 100 °C. Transesterification conversion of the crude compound was determined by ^1^H NMR spectroscopy.

### 2.16 Computational Methodology

Density functional theory (DFT) and molecular modeling calculations for **Pd-L** and **Pd-DEAA** were performed by using the Gaussian16 package at the DFT level. The molecular geometries of these complexes were fully optimized using DFT based on the B3LYP 6-31­(d,p) method along with the LANL2DZ basis set.^35–37^ These optimized structures were visualized using Gaussian View version 6.1.1.^38^

## 3 Result and Discussion

### 3.1 Synthesis and Solubility of **Metals-L**

The **Metals-L** were synthesized by reacting DEHA with metal acetate and Cs_2_CO_3_ (molar ratio of 2:1:3). Metal acetates, except for palladium, were reacted in ethanol. Palladium acetate was reacted in THF because black palladium metal was precipitated by reducing ethanol.^39^ The elements contained in these complexes were analyzed using EDX. For complexes synthesized using nickel, cobalt, and zinc acetates, cesium cations were included, as shown in Figures S1–S3. In contrast, **Pd-L** and **Cu-L** did not contain cesium cations (Figures S4 and S5). The solubilities of these complexes are summarized in Table 1. These complexes were soluble in chloroform and dichloromethane and insoluble in hexane and ethanol. These complexes did not decompose after being washed with water and were resistant to hydrolysis. Moreover, these complexes were stable up to around 200 °C in air, determined by TG-DTA (see Supporting Information).

**Table 1:** Solubilities of **Metals-L**

	solubility^a^							
complex	hexane	toluene	CH_2_Cl_2_	CHCl_3_	THF	acetone	ethanol	methanol
**Pd-L**	––	––	++	++	––	––	––	––
**Cu-L**	––	–	+	+	–	––	––	––
**Ni-L-Cs**	––	–	++	++	+	+	––	–
**Co-L-Cs**	––	–	++	++	++	+	––	–
**Zn-L-Cs**	––	–	++	++	++	+	––	–

a Solubility was estimated from the volume of solvent required to dissolve 1 mg of the complex at r.t.; ++: <1 mL, + : 1–5 mL, −: 5–10 mL, −–: >10 mL.

### 3.2 Structure of **Pd-L**, **Cu-L**, and **Zn-L-Cs**

The crystallographic data from single-crystal X-ray structural analysis are shown in Table 2. **Pd-L** was found to exist in the *trans* form, in which the palladium atom was covalently bonded to the oxygen atoms of the hydroxy and ester groups, as shown in Figure 2. The distances of Pd1–O1 and Pd1–O2 were 1.978 and 1.993 Å, respectively. The covalent bond between palladium and the oxygen atoms in the hydroxy group was shorter than that between the palladium and oxygen atoms in the ester group. For bis (troponato)­palladium­(II) (Pd­(Tp)_2_), the distances of Pd1–O1 and Pd1–O2 were 1.974 and 1.990 Å, respectively (Figure S6, CCDC: 283792).^40^ For bis­(2,4-pentanedionato)-palladium­(II) (Pd­(acac)_2_), the distance of Pd1–O1 and Pd1–O2 were 1.982 and 1.984 Å, respectively (Figure S7, CCDC: 289749).^41^ Hence, the Pd–O distances of **Pd-L** are similar to those of Pd­(Tp)_2_ and Pd­(acac)_2_. The distances between coordinated C=O (C1–O2) and free C=O (C2–O3) were 1.256 and 1.217 Å, respectively. The dihedral angle between palladium and the ligand was 172.9°, indicating a high degree of flatness, comparable to that of **Pd-DEAA** (average 175.2°) in the *cis* form.^28^

**Figure 2:**
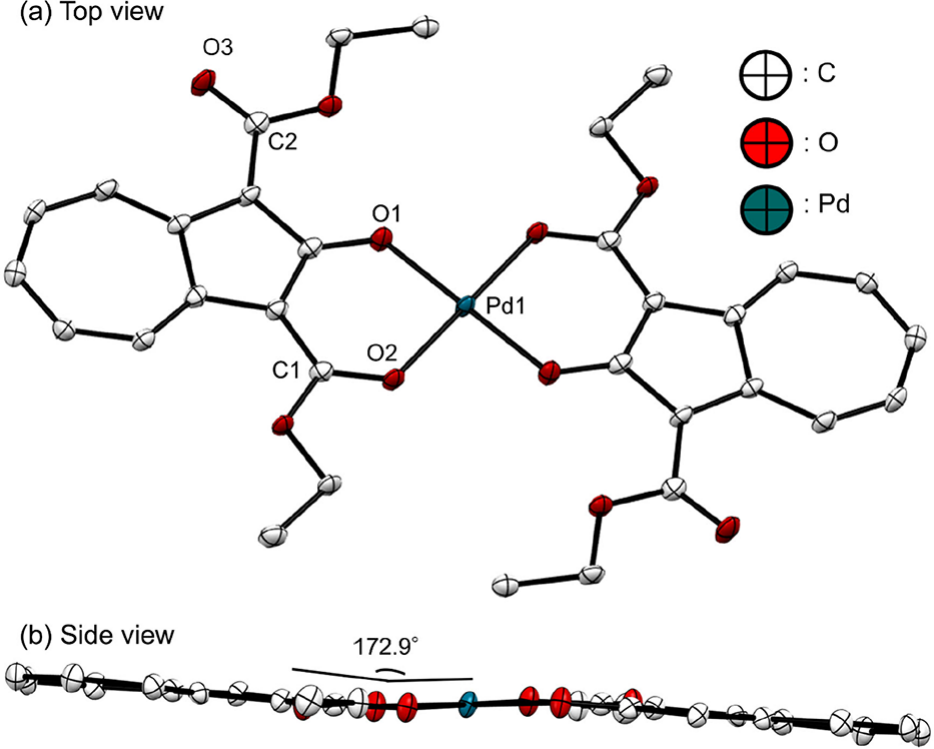
ORTEP drawing of **Pd-L** with the thermal ellipsoids shown at the 50% probability level. The hydrogen atoms are omitted for clarity.

**Table 2:** Crystallographic Data of **Pd-L**, **Cu-L**, and **Zn-L-Cs**

compound	**Pd-L**	**Cu-L**	**Zn-L-Cs**
CCDC	2406169	2423218	2406173
empirical formula	[C_32_H_30_O_10_Pd]	[C_32_H_30_O_10_Cu]	[C_48_H_43_O_15_CsZn]_4_
formula weight	680.96	638.1	4224.3
space group	*P*1̅	*P*2_1_/*c*	*P*2_1_/*n*
crystal system	triclinic	monoclinic	monoclinic
*a* (Å)	8.7784(3)	20.3360(3)	13.6998(7)
*b* (Å)	9.6728(3)	14.0929(2)	24.2443(9)
*c* (Å)	10.1115(2)	20.2291(4)	14.4629(6)
α (°)	64.908(3)	90	90
β (°)	77.909(3)	90.252(2)	113.695(5)
γ (°)	65.629(3)	90	90
*Z*	1	8	1
temp. (K)	94	295	85
*F*(000)	348	2648	2136
*V* (Å^3^)	707.65	5797.5	4398.8
*d* (g/cm^3^)	1.598	1.462	1.595
μ (mm^–1^)	0.717	0.813	1.446
reflection collected	7578	125,491	34,058
independent reflections	3207	15,119	10,147
(*R*_int_)	0.0671	0.0756	0.0891
*R*_1_ (*I* > 2σ(*I*))	0.0386	0.0860	0.0667
*wR*_2_ (all data)	0.1056	0.1684	0.1790
GOF	0.868	1.128	1.182

We tried to understand why **Pd-L** was in the *trans* form and **Pd-DEAA** was in the *cis* form. For **Pd-DEAA**, the intramolecular N–H···N hydrogen bond formation was confirmed by FTIR and single-crystal X-ray structure (CCDC: 2212893).^28^ Therefore, the free energy of *cis*-**Pd-DEAA** is low. In contrast, no intramolecular interactions were observed for **Pd-L**. As the *trans* form is most likely to reduce the intramolecular moment, **Pd-L** exists as *trans*-**Pd-L**. DFT calculations were performed for structural optimization, and vibrational calculations were applied to compare the stabilities of *the cis* and *trans* forms, as shown in Figure 3. The thermodynamic energy difference for **Pd-L** was 32.4 kJ/mol, indicating that *trans*-**Pd-L** was more stable. The thermodynamic energy difference of **Pd-DEAA** was 19.2 kJ/mol, and *cis*-**Pd-DEAA** was more stable. This result is consistent with the single-crystal structure observed in the analysis.

**Figure 3:**
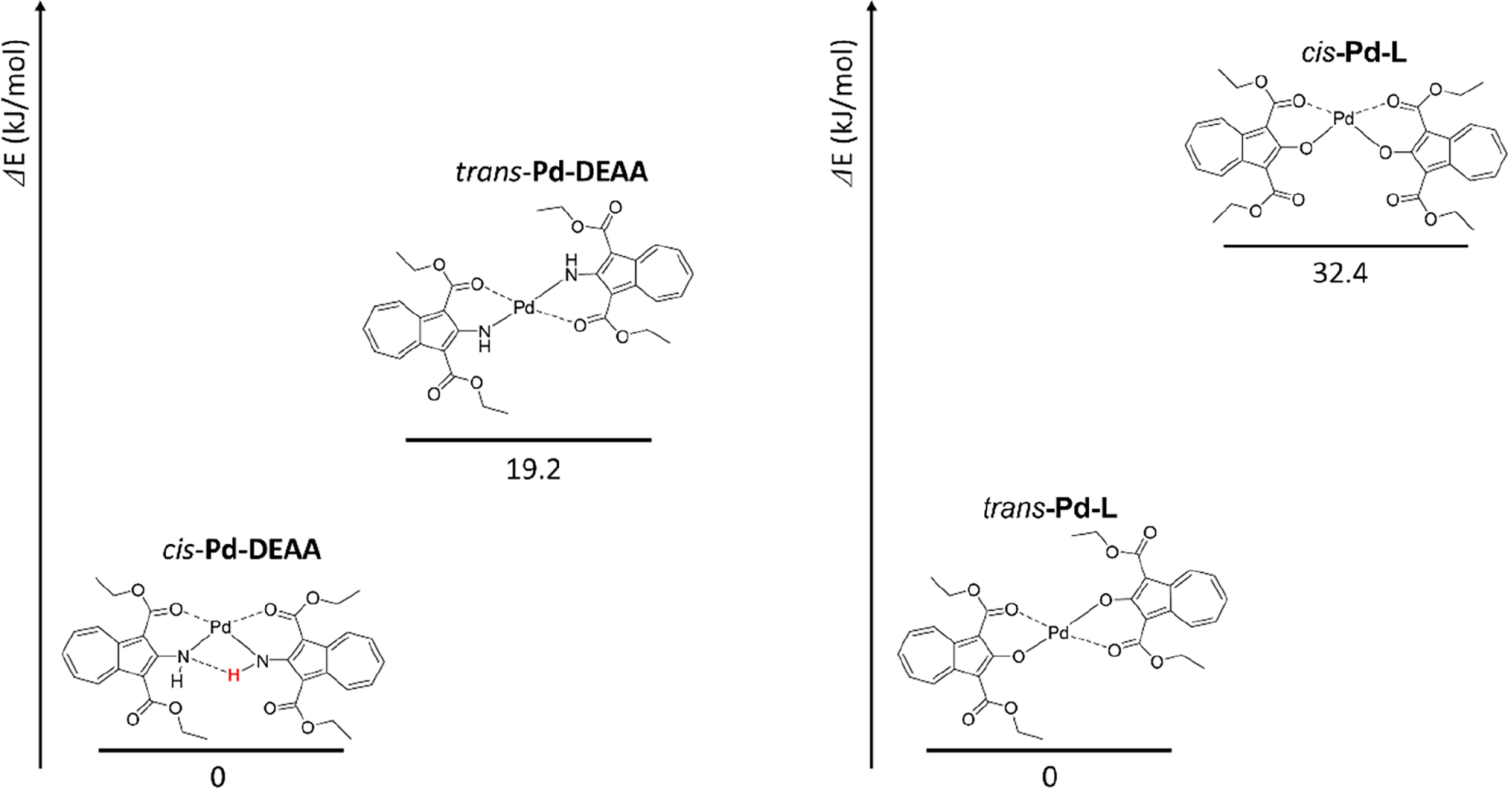
Comparison of the thermodynamic energies of **Pd-DEAA** (left) and **Pd-L** (right) calculated using DFT.

**Cu-L** was confirmed to be a dimer of centrosymmetric axial- and basal-edged square pyramids,^42^ and the coordination number of the copper atoms was five, as shown in Figure 4. The distances of the covalent bonding Cu–O (such as for Cu1–O1) and coordination bonding Cu–O (such as for Cu1–O2) were 1.888–1.904 and 1.937–1.958 Å, respectively. The distances of the copper-atom (μ_2_-O) of Cu1–O3 and Cu2–O2 differed depending on whether the μ_2_-O was derived from the hydroxy or ester groups (2.631 and 2.695 Å, respectively). The average dihedral angle between copper and the ligand was 168.7°. The flatness of **Cu-L** is lower than that of **Pd-L**, probably because of the strain from dimerization.

**Figure 4:**
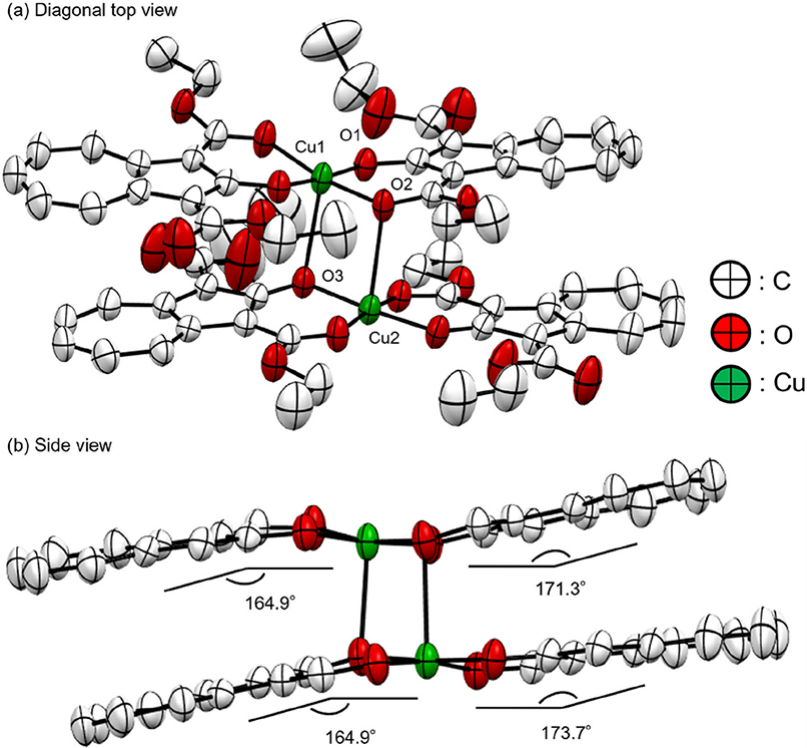
ORTEP drawing of **Cu-L** with the thermal ellipsoids shown at the 50% probability level. The hydrogen atoms are omitted for clarity. For the side view, the ethoxy- and free-carboxylate groups are omitted for clarity.

The molecular structure of **Zn-L-Cs** was composed of three ligands, one zinc atom, and one cesium atom, and **Zn-L-Cs** was a one-dimensional bridged Zn­(II)–Cs­(I) coordination polymer, [ZnCs­(OC_10_H_5_(COOC_2_H_5_)_2_)_3_]_*n*_, as shown in Figure 5. The coordination numbers of Zn and Cs were six and nine, respectively, with one zinc atom and one cesium atom bound to three and five ligands, respectively. The Zn–O and Cs–O distances were 2.012–2.169 and 3.057–3.312 Å, respectively. The average dihedral angle between zinc and the ligand was 173.7°. Although azulene-based coordination polymers have been reported, such as tin azulenyl-1-carboxylate [Me_3_Sn­(OOCC_10_H_7_)]_*n*_,^43^ to the best of our knowledge, **Zn-L-Cs** is the first polymer composed of a new type of coordination polymer of azulene.

**Figure 5:**
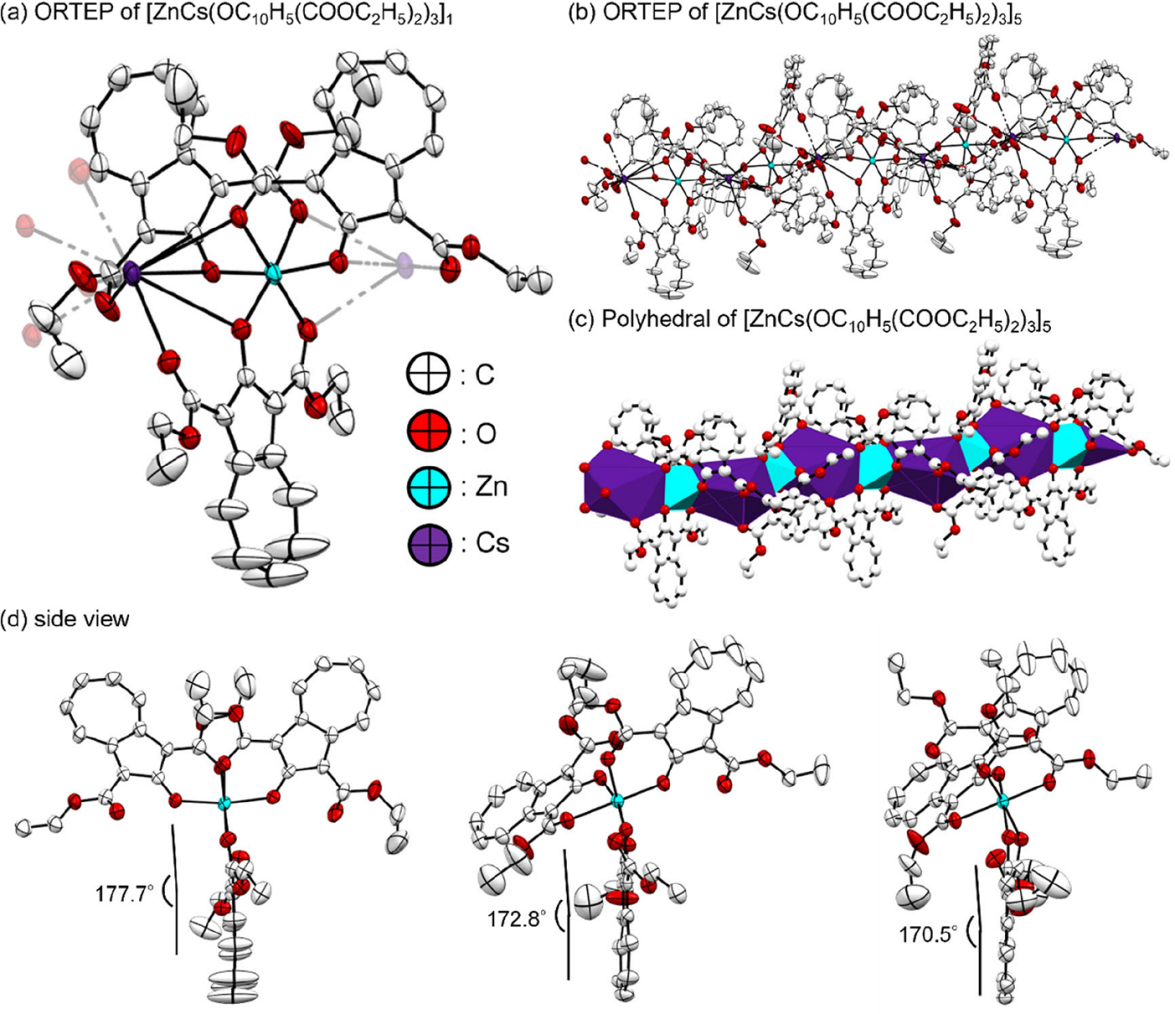
ORTEP drawing of **Zn-L-Cs** with the thermal ellipsoids shown at the 50% probability level. The hydrogen atoms are omitted for clarity. ((a) ORETP of [Zn­(Cs­(OC_10_H_5_(COOC_2_H5)_2_)_3_]_1_, (b) ORTEP of [ZnCs­(OC_10_H_5_(COOCsH_5_)_2_)_3_]_5_ (c) polyhedral of [ZnCs­(OC_10_H_5_(COOC_2_H_5_)_2_)_3_]_5_ (d) side view).

### 3.3 Spectra of **Metals-L**

The **Metals-L** were characterized using ^1^H NMR and FTIR spectroscopy. However, ^1^H NMR spectra of **Cu-L**, **Co-L-Cs**, and **Ni-L-Cs** showed broadened signals owing to the paramagnetism of these metals (Figure S8). The ^1^H NMR spectra of DEHA, **Pd-L**, and **Zn-L-Cs** are shown in Figure 6. The signals due to the ethoxy and azulenyl moieties in **Pd-L** and **Zn-L-Cs** shifted to higher magnetic fields compared to those in DEHA. This shift can be explained by nucleus-independent chemical shifts (NICS). The NICS(1)_*zz*_ index was adopted because its calculation is computationally easy and highly accurate.^44^ The NICS(1)_*zz*_ values of the seven-membered rings of DEHA, **Pd-L**, and **Zn-L-Cs** were −26.0670,–23.4008, and −18.3260, respectively, indicating that the aromaticity of **Pd-L** and **Zn-L-Cs** was reduced compared with that of DEHA.^45,46^ Furthermore, bond alternations of DEHA (CCDC: 1100333),^47^ **Pd-L**, and **Zn-L-Cs** were investigated in their single crystal structure (Figures S9–S11), and aromaticity was evaluated from the Harmonic Oscillator Model of Aromaticity (HOMA) value.^48^ HOMA calculates the difference between the ideal bond lengths of aromatic ring structure and the actual bond lengths, normalizing this value to use as an indicator of aromaticity (the fewer the ideal bond length and the actual bond length, the smaller the value of HOMA will be than 1). The results showed that the values of HOMA for 5-, 7-, and 10-membered rings in **Pd-L** and **Zn-L-Cs** were smaller than those in DEHA (Table 3), suggesting a decrease in aromaticity due to complex formation.^49^ This may be due to the formation of a heterometallacycle, which shortens the C–O bond at the 2-position (1.329 Å in DEHA; 1.283Å in **Pd-L**; 1.263 Å in **Zn-L-Cs**) and the C–C bond at the 1-position (1.453 Å in DEHA; 1.434 Å in **Pd-L**; 1.435 Å in **Zn-L-Cs**) of the azulene ring, resulting in a direct like double bond to the azulene ring.^50^ These results supported a shift in the ^1^H NMR signals of the complexes.

**Table 3:** HOMA of **DEHA**, **Pd-L**, and **Zn-L-Cs**

compound	HOMA (5-membered-ring)	HOMA (7-membered-ring)	HOMA (10-membered-ring)
DEHA	0.8423	0.9025	0.9859
**Pd-L**	0.7324	0.8512	0.9443
**Zn-L-Cs**	0.6681	0.8530	0.9211

**Figure 6:**
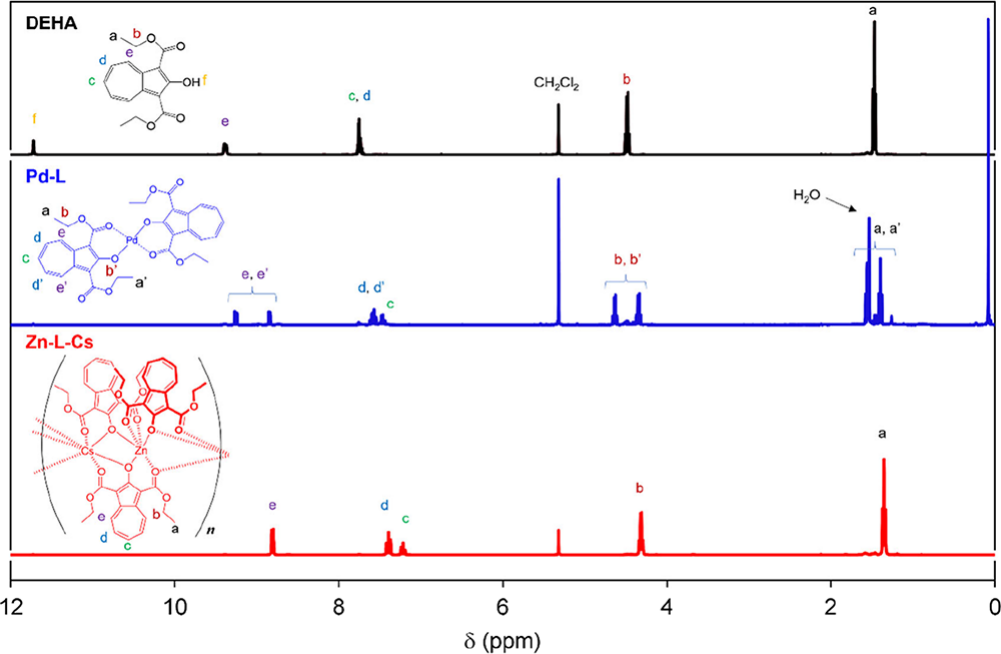
^1^H NMR spectra of DEHA, **Pd-L**, and **Zn-L-Cs** in CD_2_Cl_2_.

The FTIR spectra of DEHA and **Metals-L** are shown in Figure 7. The absorption bands of DEHA at 1673 and 1637 cm^–1^ correspond to the carbonyl group, υC=O.^51,52^ The absorption band of the coordinated carbonyl group in these complexes was shifted to lower wavenumbers (from 1637 to 1600–1590 or 1540 cm^–1^) compared with that of DEHA, indicating the complexation.^53,54^ For **Ni-L-Cs**, **Co-L-Cs**, and **Zn-L-Cs**, the spectral pattern at 1135, 1095, 1026, and 603 cm^–1^ was very similar. These absorption bands can be attributed to the skeletal vibrations of the azulene-based coordination polymer. The structures of **Ni-L-Cs** and **Co-L-Cs** are believed to be of the type [MCs­(OC_10_H_5_(COOC_2_H_5_)_2_)_3_]_*n*_ (M = Ni, Co) because (i) the skeletal vibration of the azulene-based coordination polymer was observed in the FTIR spectrum, (ii) [M­(OC_10_H_5_(COOC_2_H_5_)_2_)_3_]^−^ (M = Ni, Co) was observed by mass spectroscopy, and (iii) the value of elemental analysis was consistent with that of MCs­(OC_10_H_5_(COOC_2_H_5_)_2_)_3_. The FTIR assignments of DEHA and these complexes are summarized in Table 4.

**Figure 7:**
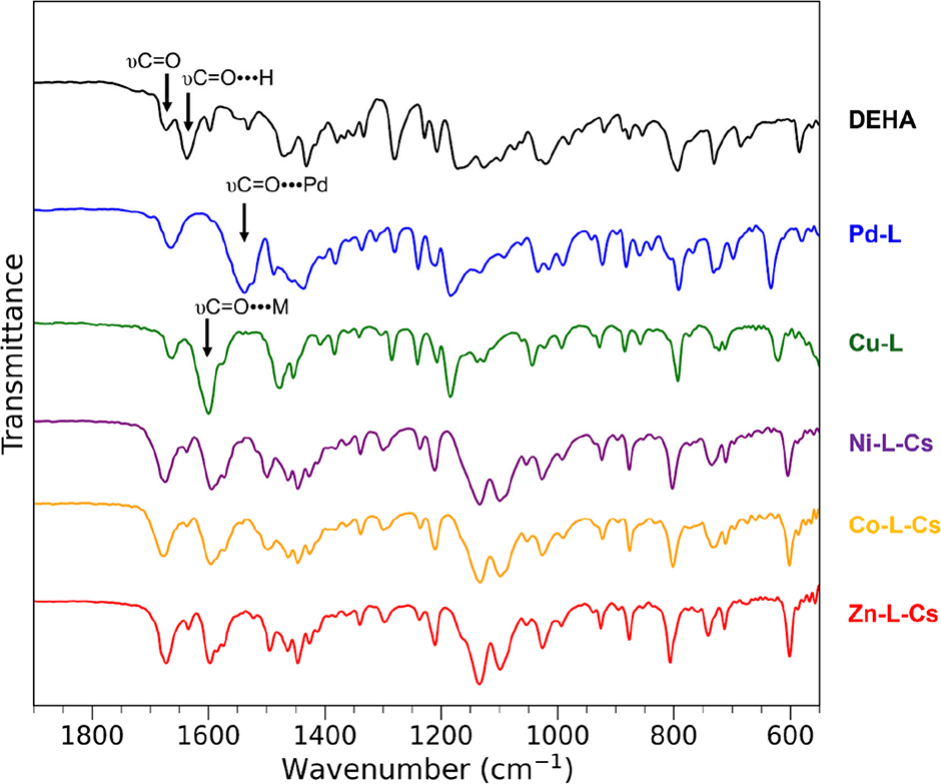
FTIR spectra of DEHA and DEHA complexes using the ATR method.

**Table 4:** FTIR Assignment of DEHA and DEHA Complexes

wavenumber (cm^–1^)						
DEHA	**Pd-L**	**Cu-L**	**Ni-L-Cs**	**Co-L-Cs**	**Zn-L-Cs**	assignment^a^
1673	1666	1660	1671	1681	1677	υC=O
1639						υC=O of hydrogen bond
	1540	1591	1594	1600	1600	υC=O of coordinated C=O
1019	1014	1015	1026	1026	1026	ρCH_3_
			604	602	602	

a υ, stretching; ρ, rocking.

The UV–Vis spectra of DEHA and these complexes are shown in Figure 8 and Table 5. The S_0_–S_1_ transition of DEHA was observed at approximately 440 nm;^29^ however, this transition was too weak compared to its complex. The absorption band at approximately 363 nm corresponded to the S_0_–S_2_ transition of DEHA.^29^ The strongest absorption band was attributed to the S_0_–S_3_ and S_0_–S_4_ transitions by TD-DFT calculation (Figure S12), and this band in CH_2_Cl_2_ and THF was observed at 313 nm, whereas in methanol, it was blue-shifted by 2 nm. The S_0_–S_1_ transitions of these complexes were clearly observed at 450–470 nm. The S_0_–S_2_ transitions of these complexes in CH_2_Cl_2_ and THF were observed at 388–406 nm. The absorption band due to the S_0_–S_3_ and S_0_–S_4_ transitions of these complexes in CH_2_Cl_2_ and THF was red-shifted compared to that of DEHA to 330–340 nm.

**Figure 8:**
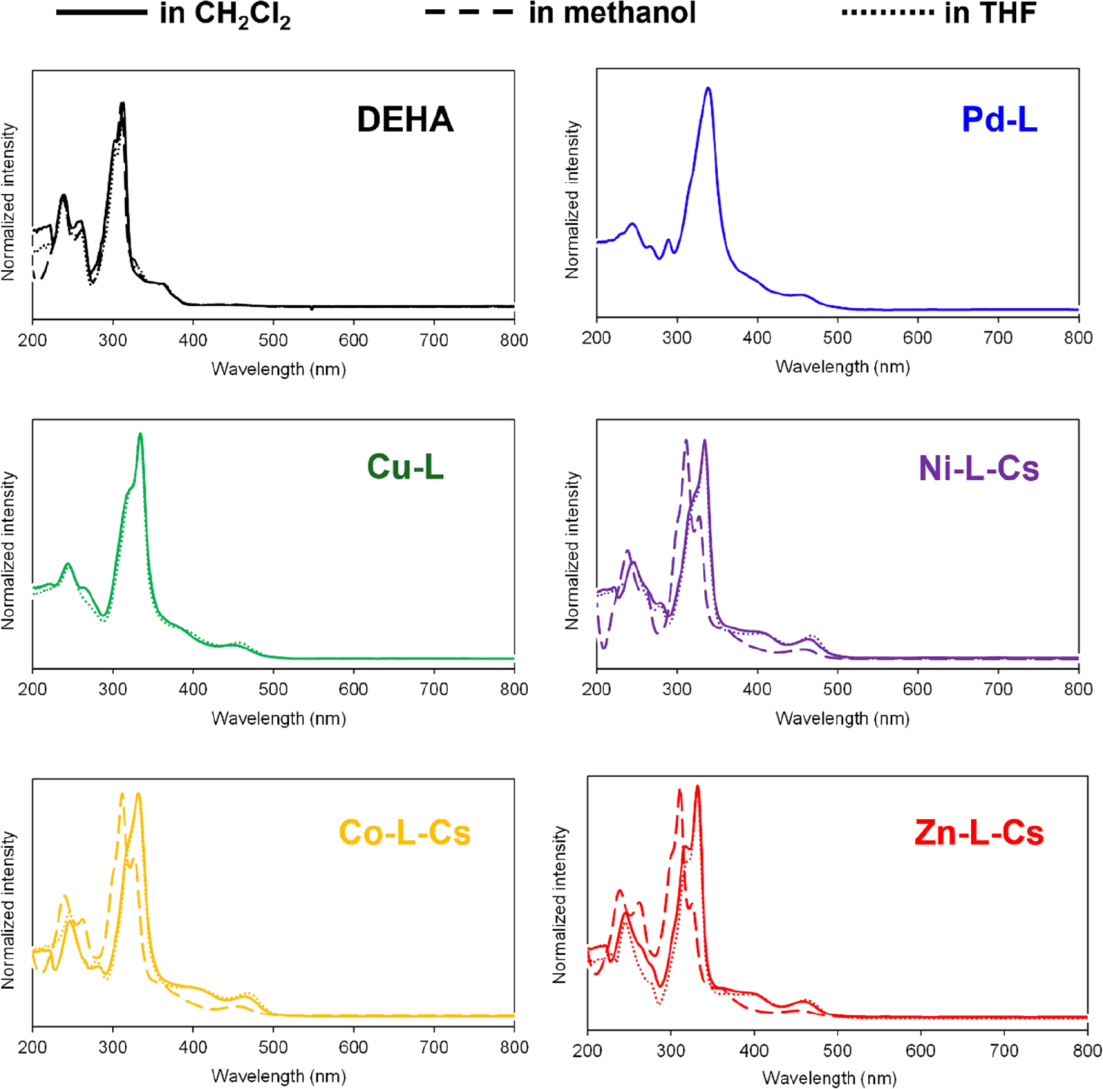
UV–Vis spectra of DEHA and the DEHA complexes in CH_2_Cl_2_ (solid line), CH_3_OH (dashed line), and THF (dot line).

**Table 5:** UV–Vis Wave Absorption of DEHA and These Complexes in CH_2_Cl_2_, THF, and CH_3_OH

	wavelength (nm) (CH_2_Cl_2_/methanol/THF)					
assignment	DEHA	**Pd-L**	**Cu-L**	**Ni-L-Cs**	**Co-L-Cs**	**Zn-L-Cs**
S_0_–S_1_	430/451/439	456/-/-	453/-/457	463/458/468	465/456/468	459/454/463
S_0_–S_2_	363/361/365	395/-/-	388/-/392	403/-/406	406/-/ 401	397/-/ 399
S_0_–S_3,_ S_0_–S_4_	313/311/313	339/-/-	335/-/335	335/328/335	332/362/332	332/326/332
	-/-/-	316/-/-	322/-/322	323/312/323	319/312/319	319/311/318
	302/302/303	-/-/-	-/-/-	-/300/-	-/301/-	-/301/-

The UV–Vis spectra of **Ni-L-Cs**, **Co-L-Cs**, and **Zn-L-Cs** in methanol were different from those in CH_2_Cl_2_ and THF. To investigate the reason for this observation, titration was conducted by adding methanol to the **Zn-L-Cs** solution. When increasing the titration amount of methanol added to the **Zn-L-Cs**/CH_2_Cl_2_ solution, the absorption intensities at 332, 397, and 459 nm decreased, whereas those at 303 and 313 nm increased, as shown in Figures 9 and S13. The strongest absorption was blue-shifted from 332 to 313 nm, and the maximum absorption was similar to that for DEHA. The methanol-induced degradation of **Zn-L-Cs** (possibility of dissociation of the DEHA ligand) was suggested by UV–Vis titration. To validate this hypothesis, ^1^H NMR titration was performed (Figure S14). Increasing the titration amount of methanol in the **Zn-L-Cs**/CD_2_Cl_2_ solution resulted in only a minor dissociation of DEHA. Moreover, there was no change in the signals of the azulenyl moiety before and after methanol titration, indicating that the structure and aromaticity of **Zn-L-Cs** were not changed. Furthermore, the FTIR spectra of **Zn-L-Cs** before and after methanol dissolution were measured, but no significant change was observed (Figure S15). These results suggest that the UV–Vis change in methanol was not due to the structural change or degradation of **Zn-L-Cs** but due to the solvent effect, such as the interaction between the two azulene derivatives or between the azulene derivative and the solvent molecule.

**Figure 9:**
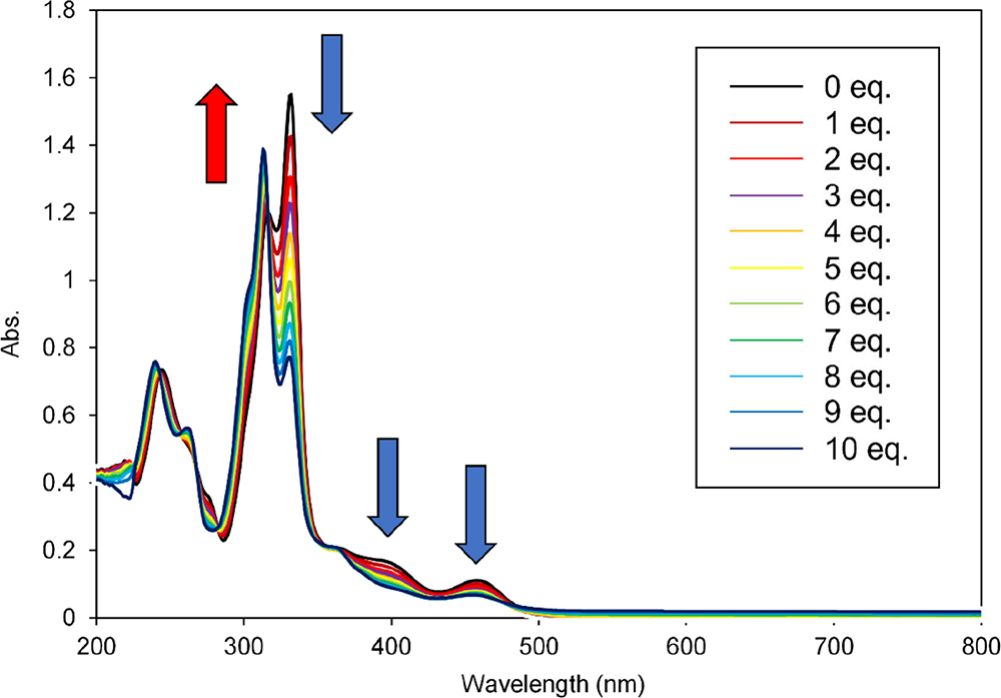
UV–vis spectra of **Zn-L-Cs** in CH_2_Cl_2_ upon increasing addition of methanol.

The fluorescence spectra of DEHA and these complexes are shown in Figure 10. In the TD-DFT calculation of DEHA, oscillator strengths of S_1_ → S_0_ and S_2_ → S_0_ were 0.0026 (λ_em_ = 491 nm) and 0.1678 (λ_em_ = 367 nm), respectively (Figures S16–S18). Fluorescence emission of DEHA showed a broad peak at 320–500 nm (peak center was around 400 nm), suggesting that the emission is caused by S_2_ → S_0_ fluorescence (Figure S17). This result was similar to that of the reported azulene derivatives,^55–57^ and this behavior has been reported as the anti-Kasha rule.^58^ For **Metals-L**, only **Zn-L-Cs** exhibited fluorescence emission (λ_em_ = ca. 530 nm), corresponding to S_1_ → S_0_ fluorescence (Figure S19), similar to KGd­(OC_10_H_5_(COOC_2_H_5_)_2_)_4_ reported by Zhang and Petroud.^29^ Hence, the appearance of fluorescence emission for **Metals-L** depended on the metal species, and **Zn-L-Cs** showed emission.

**Figure 10:**
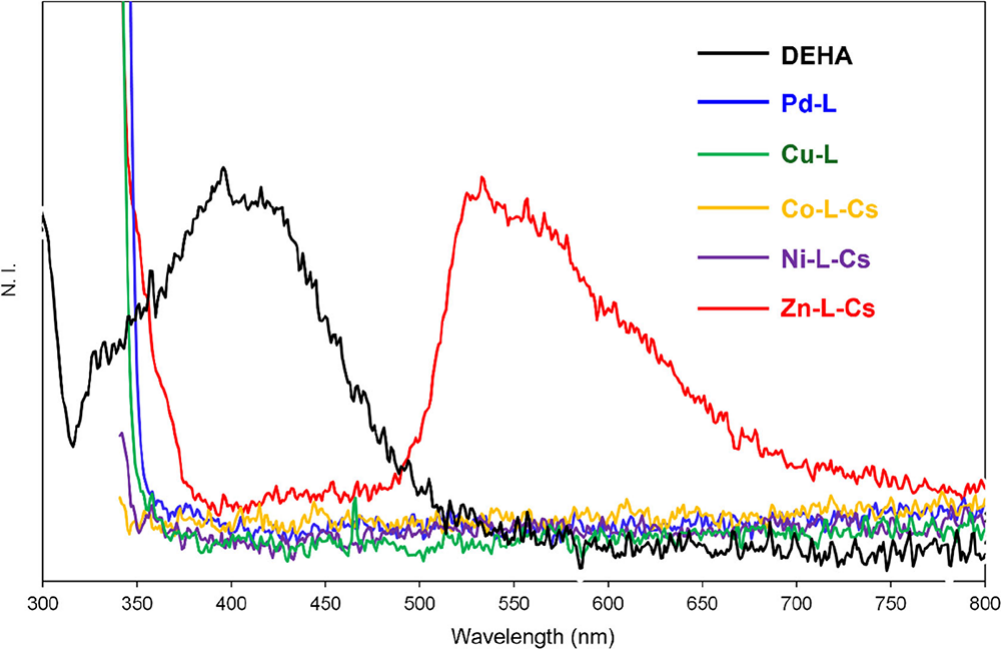
Fluorescence spectra of DEHA and these complexes in CH_2_Cl_2_ (λ_ex_ = 310 nm, DEHA; λ_ex_ = 330 nm, **Metals-L**; ca. 25 °C).

PMMA composite films containing 0.1, 1, and 3 wt % of DEHA, **Pd-L**, **Cu-L**, **Co-L-Cs**, **Ni-L-Cs**, and **Zn-L-Cs** were prepared for application as dyes dispersed in polymers. The appearance and UV–Vis spectra of the PMMA composite films are shown in Figures 11 and 12, respectively. In DEHA, **Co-L-Cs**, and **Zn-L-Cs**, the films appeared transparent and colored due to dyes. In 3 wt % PMMA–**Pd-L**, **Cu-L**, and **Ni-L-Cs**, a cloudy and mottled appearance was observed. UV–Vis spectra indicated reduced transparency, and the presence of needle-like aggregation and holes was confirmed by SEM (Figure S20). Elemental mapping analysis clearly revealed that **Pd-L** showed pronounced aggregation behavior (Figures S21–S25). The difference in dispersibility is considered to be related to the solubility. During the concentration in a drying process to form composite films, **Pd-L**, **Cu-L**, and **Ni-L-Cs**, which have lower solubility, have formed aggregates in the PMMA matrix. From FTIR spectra, no shift of carbonyl groups around 1730 cm^–1^ of PMMA^59^ was observed (Figures S27 and S28). The composite films exhibited improved thermal stability, as reflected by higher temperatures of 20% weight loss (*T*_d20_) compared to pure PMMA (Figures S29–S33). However, the temperatures of 10% weight loss (*T*_d10_) were almost the same. When a strong intermolecular interaction, such as hydrogen bonding, occurs between PMMA and the additive, it is known that even a small amount of the additive tends to increase *T*_d10_.^59^ Accordingly, **Metals-L**s are presumed to exist physically in mixing with PMMA.

**Figure 11:**
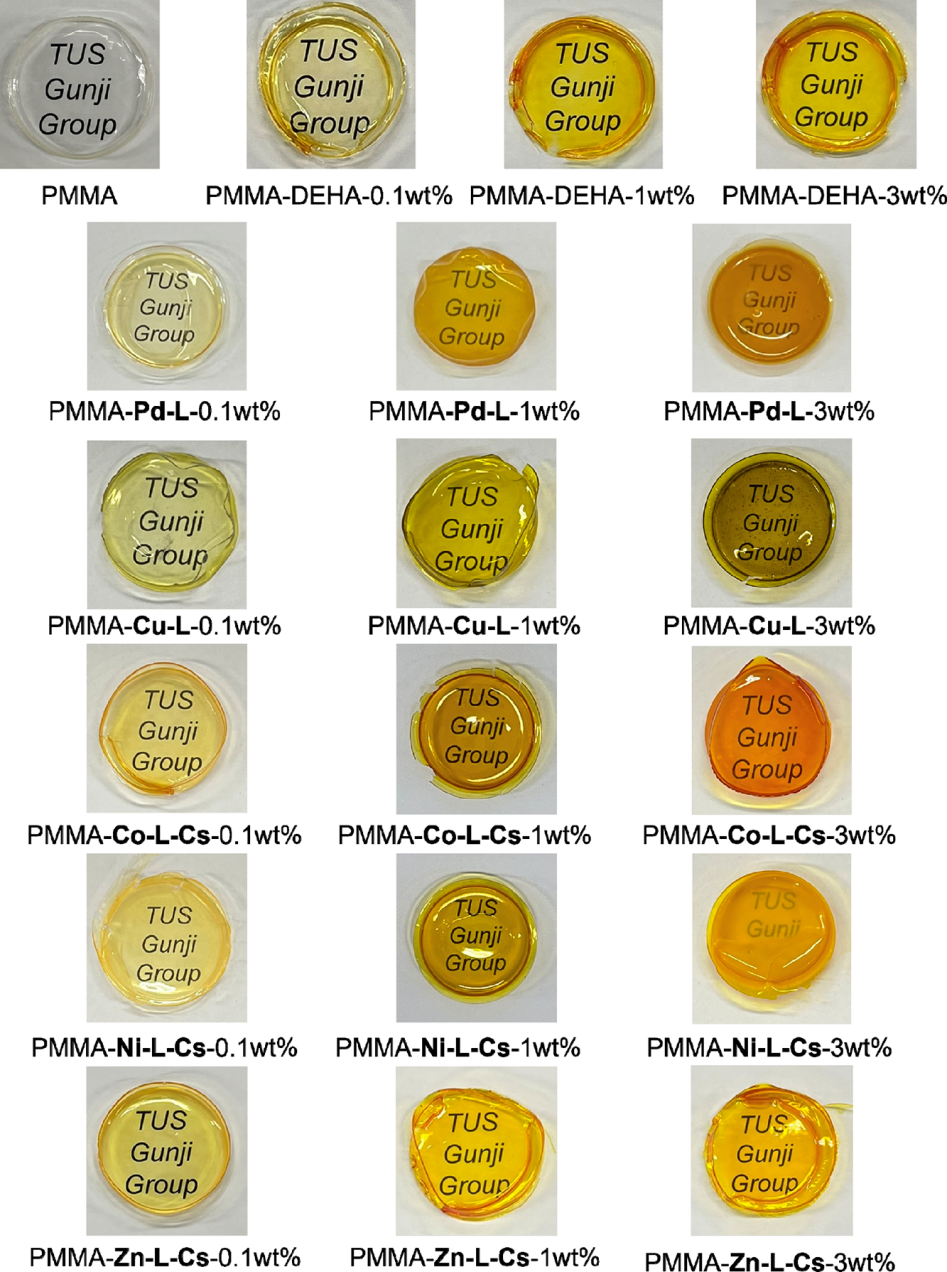
Appearance of PMMA composite films.

**Figure 12:**
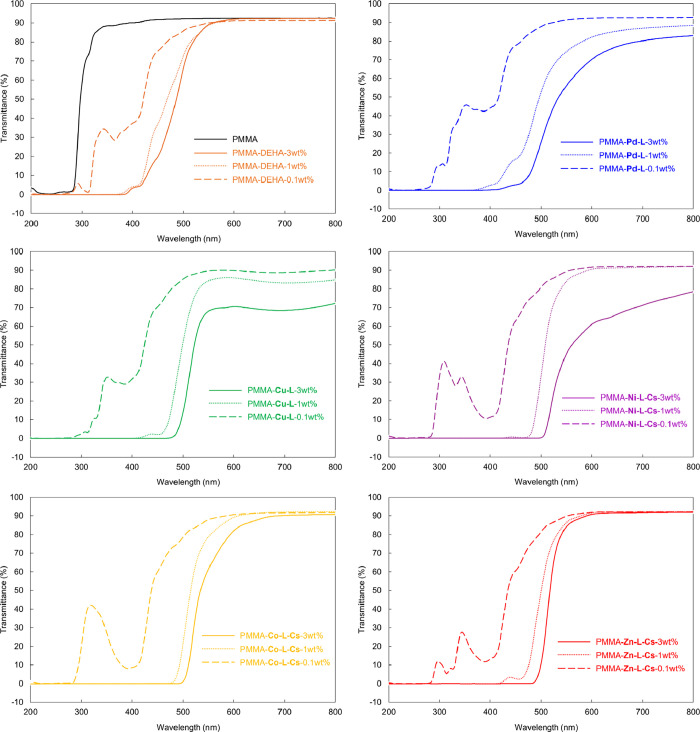
UV–Vis spectra of PMMA composite films.

### 3.4 Transesterification of DEHA and DEAA

We attempted to synthesize Ti­(O*i*Pr)_2_(OC_10_H_5_(COOC_2_H_5_)_2_)_2_ by the reaction of DEHA with Ti­(O*i*Pr)_4_ in the presence of Cs_2_CO_3_ in *i*PrOH. Yellow powder was obtained by evaporation of the reaction solution at 100 °C under reduced pressure and extraction with CHCl_3_. The ^1^H NMR spectrum of the yellow powder is shown in Figure 13. The signals due to the azulenyl moieties were confirmed at 7.5–12 ppm and were at the same position as in DEHA, suggesting that the target complex was not formed and no decomposition of the azulenyl skeleton had occurred. The ethyl group in the ester group (1.5 and 4.5 ppm) almost disappeared, while the isopropyl group in the ester group (1.5 and 5.4 ppm) was observed. The yellow powder was also determined to be diisopropyl 2-hydroxyazulene-1,3-dicarboxylate (D*i*PrHA) by mass spectrometry (Figure S34); hence, the transesterification from ethyl to isopropyl groups had occurred (Scheme 3). Based on the intensity ratio of CH_2_ in the ethyl group to CH in the isopropyl group, the transesterification conversion rate was 85% that of the isopropyl groups. When either Ti­(O*i*Pr)_4_ or Cs_2_CO_3_ was added, and the mixture was heated, transesterification was not observed. Therefore, the addition of Ti­(O*i*Pr)_4_ and Cs_2_CO_3_ was necessary for the transesterification of DEHA. The reaction was performed using *n*BuOH instead of *i*PrOH. The transesterification conversion rate of the butyl group was >99%, and dibutyl 2-hydroxyazulene-1,3-dicarboxylate (D*n*BuHA) was formed. The transesterification of DEHA was also performed by using allyl alcohol. The transesterification conversion rate of the allyl group was more than 99%, forming diallyl 2-hydroxyazulene-1,3-dicarboxylate (DAHA). When this solution was evaporated at 100 °C, the residual powder exhibited (i) lower solubility in organic solvents, (ii) broadened signals in ^1^H NMR spectroscopy, and (iii) a molecular weight of approximately 1200 g mol^–1^ as determined by GPC. It was suggested that DAHA undergoes decomposition (oligomerization) upon heating; therefore, evaporation must be performed at a low temperature. To evaporate at low temperature, the mixture was washed with water, extracted with dichloromethane, and evaporation. In the ^1^H NMR spectrum of the mixture, the asterisk (*) marked signals attributed to byproducts were observed. The byproducts can be removed by passing them through silica gel, suggesting that the byproduct is titanium complexes such as Ti­(OAllyl)_4_ and Ti­(OC_10_H_5_(COOAllyl)_2_)_2_(OAllyl)_2_.

**Figure 13:**
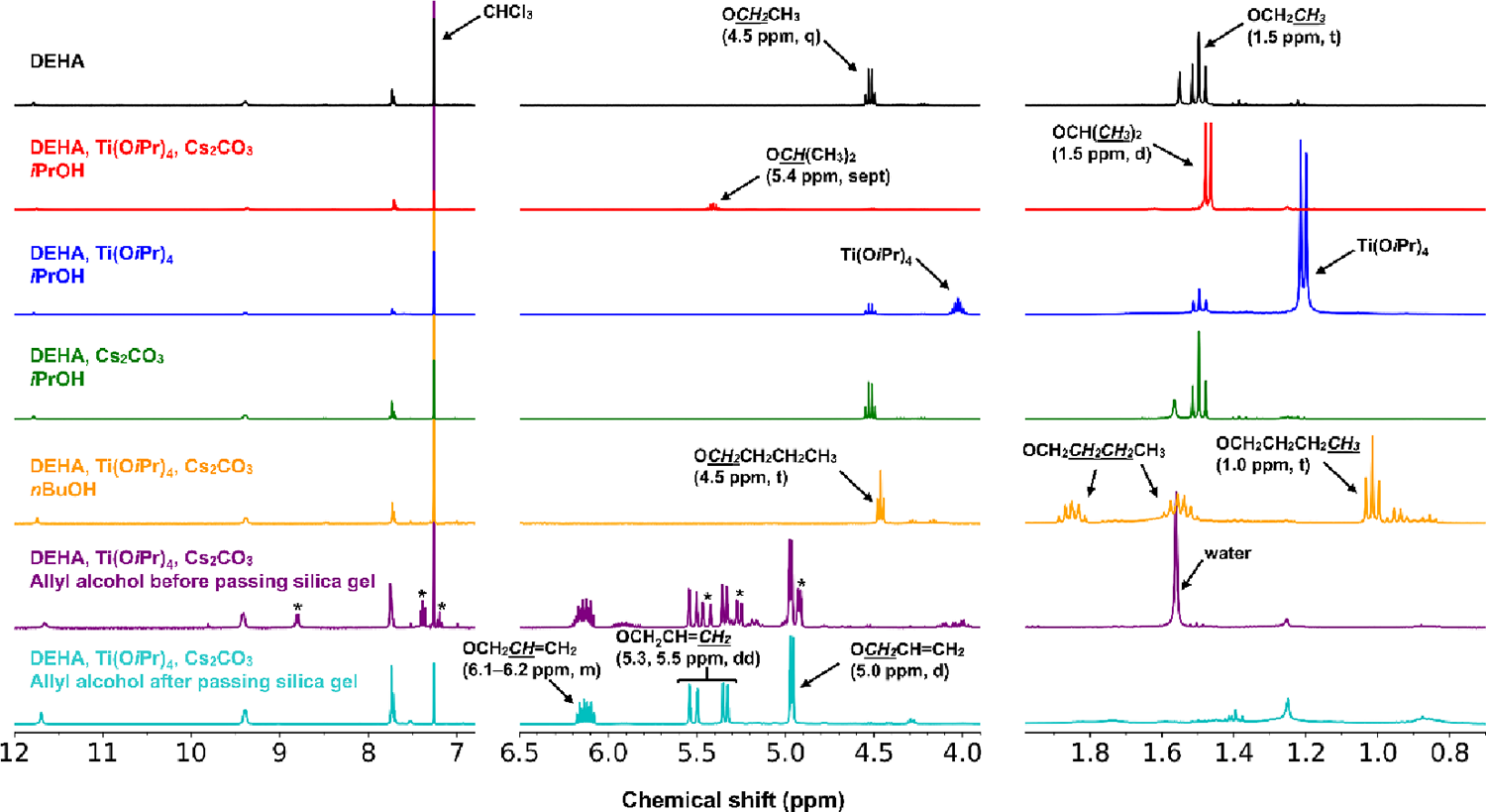
^1^H NMR spectra of the reaction of DEHA in alcohols with Ti­(O*i*Pr)_4_ and/or Cs_2_CO_3_ in CDCl_3_.

**Scheme 3:**
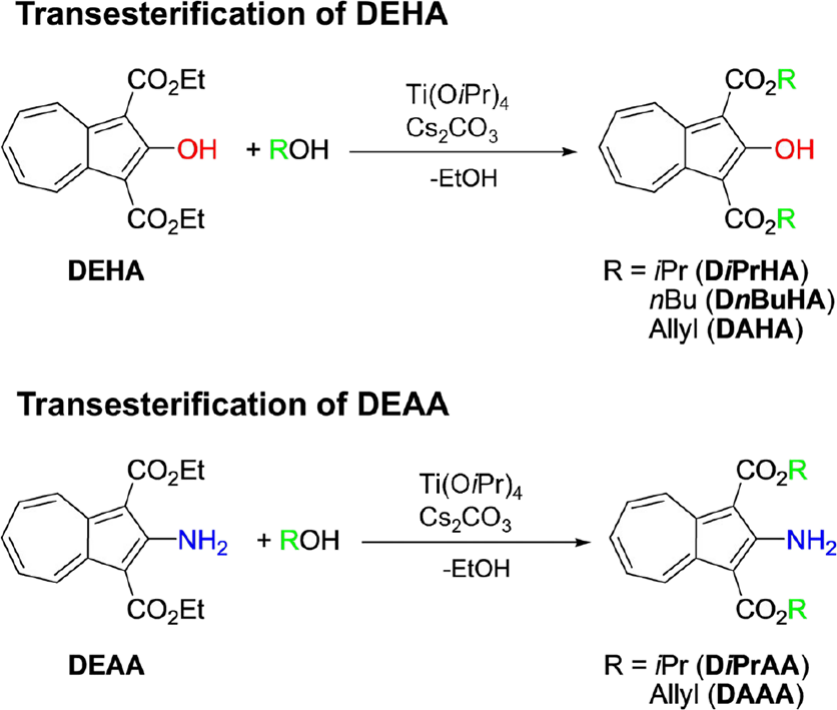
Transesterification of DEHA and DEAA

We investigated the transesterification of DEHA using a classical method with both acidic and basic catalysts because transesterification of alkyl azulene-carboxylates, which do not have a hydroxy group, can proceed with these methods.^60,61^ DEHA in *i*PrOH was stirred at 80 °C in the presence of aqueous HCl, but no transesterification was observed. Unfortunately, the signals observed at approximately 7 ppm suggest that some degradation reactions occurred (Figure S35). No transesterification was observed when DEHA and H_2_SO_4_ were mixed in *i*PrOH. When DEHA and NaOH were mixed in *i*PrOH, no progress in transesterification was observed, but the formation of **Na-L** (yield 85%) was confirmed by ^1^H NMR (Figure S36). Following other classical transesterification methods, we chose to use Al­(O*i*Pr)_3_ instead of Ti­(O*i*Pr)_4_.^62,63^ In this reaction, the yellow powder was precipitated quickly with a yield of 0.09 g from 0.10 g of DEHA. The yellow powder was confirmed to form a complex with DEHA by ^1^H NMR (Figure S28) and to contain aluminum and cesium atoms by EDX. Although we could not obtain a single crystal from this yellow powder (**Al-L-Cs**), we believe that **Al-L-Cs** was AlCs­(OC_10_H_5_(COOC_2_H_5_)_2_)_4_ based on the similarity of their structures to KLn­(OC_10_H_5_(COOC_2_H_5_)_2_)_4_ (Ln = Pr, Nd, Gd, Ho, Er, Tm, Yb, Lu).^29^ Assuming **Al-L-Cs** to be the estimated structure, 78% of DEHA was consumed for complexation. After the **Al-L-Cs** was filtered off, ^1^H NMR of the filtrate confirmed that transesterification had progressed by a small amount (D*i*PrHA 9%, DEHA 91%), as shown in Figure S23. The optimal conditions for the transesterification of DEHA were Ti­(O*i*Pr)_4_ and Cs_2_CO_3_ in alcohol (Table 6).

**Table 6:** Results of the Transesterification of DEHA and DEAA

azulene	reagent	base	alcohol^a^	product^b^	rate (%)^c^
DEHA	Ti(O*i*Pr)_4_	Cs_2_CO_3_	*i*PrOH	D*i*PrHA	85
DEHA	Ti(O*i*Pr)_4_		*i*PrOH	N.R.	0
DEHA		Cs_2_CO_3_	*i*PrOH	N.R.	0
DEHA	Ti(O*i*Pr)_4_	Cs_2_CO_3_	*n*BuOH	D*n*BuHA	>99
DEHA	HCl aq.		*i*PrOH	decomp.	0
DEHA	H_2_SO_4_		*i*PrOH	N.R.	0
DEHA		NaOH	*i*PrOH	**Na-L**	85
DEHA	Al(O*i*Pr)_3_	Cs_2_CO_3_	*i*PrOH	**Al-L-Cs**	78
DEHA	Ti(O*i*Pr)_4_	Cs_2_CO_3_	Allyl–OH	DAHA	>99
DEAA	Ti(O*i*Pr)_4_	Cs_2_CO_3_	*i*PrOH	D*i*PrAA	52
DEAA	Ti(O*i*Pr)_4_	Cs_2_CO_3_	Allyl–OH	DAAA	11

a *i*PrOH, *n*BuOH, and Allyl-OH are isopropyl, normalbutyl, and allyl alcohols.

b N.R. means “No Reaction”.

c The generation rate was measured using ^1^H NMR spectra.

The transesterification of DEAA with alcohols (*i*PrOH and allyl alcohol) was also confirmed (Figure 14). However, the transesterification rate of DEAA was slower than that of DEHA, requiring a longer reaction time. For the reaction of DEAA with allyl alcohol, the yield of diallyl 2-aminoazulene-1,3-dicarboxylate (DAAA) depended on reaction times (6 h: transesterification rate 11%, more than 24 h: transesterification >99%).

**Figure 14:**
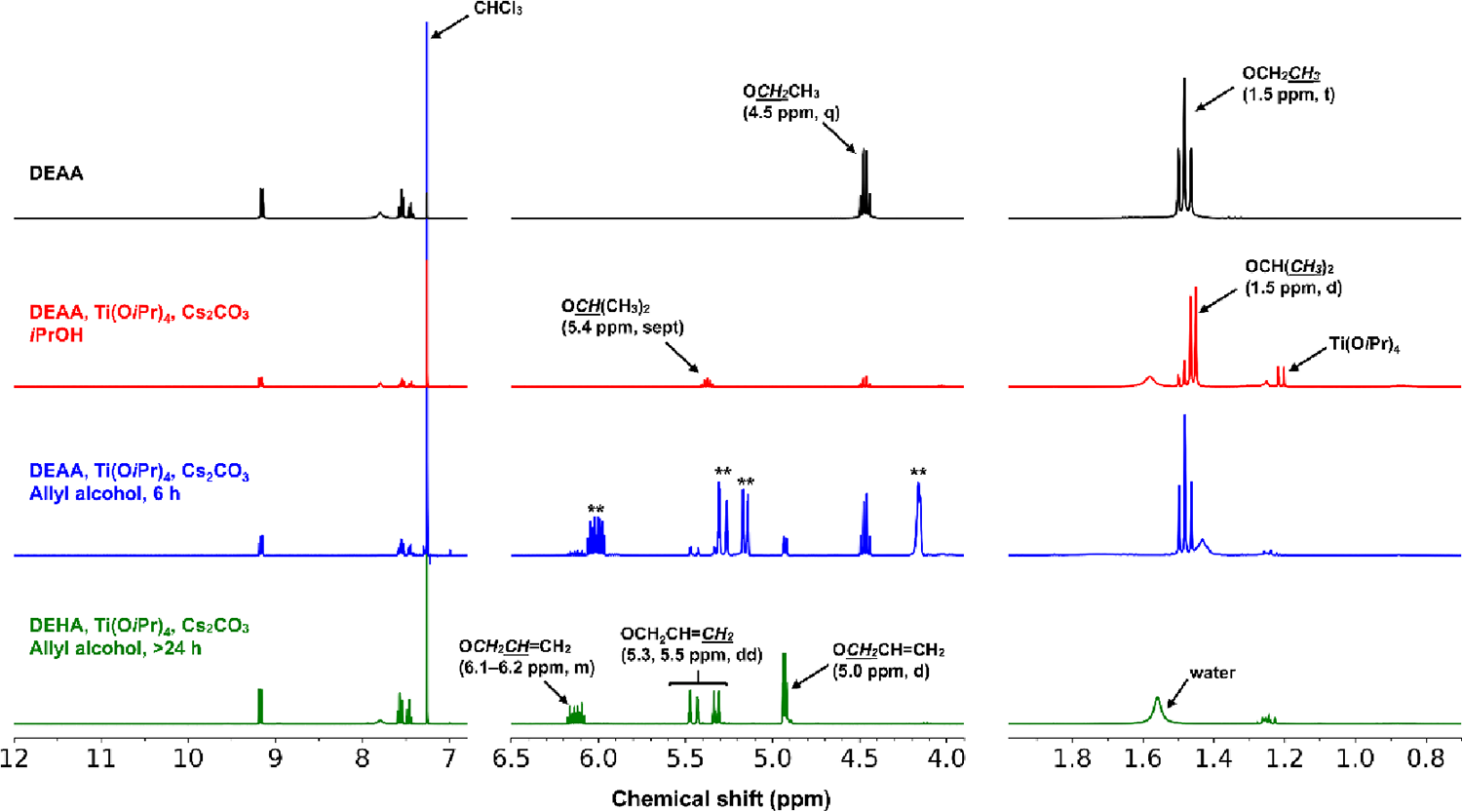
^1^H NMR spectra of the reaction of DEAA in alcohols with Ti­(O*i*Pr)_4_ and Cs_2_CO_3_ in CDCl_3_. The double asterisk (**) marked signals are due to the allyl alcohol.

This transesterification method is simple and straightforward and can be used to synthesize a variety of 2-hydroxy and 2-aminoazulene esters using various alcohols. Especially, the successful introduction of allyl groups is expected to facilitate polymerization and materialization.

## 4 Conclusions

Metal-DEHA complexes (**Metals-L**) were formed when DEHA was reacted with metal acetates M­(OAc)_2_ (M = Pd, Cu, Ni, Co, Zn) and Cs_2_CO_3_. Single-crystal X-ray structural analysis revealed that **Pd-L** and **Cu-L** had a mononuclear planar structure (Pd­(OC_10_H_5_(COOC_2_H_5_)_2_)_2_) and a binuclear structure ([Cu­(OC_10_H_5_(COOC_2_H_5_)_2_)_2_]_2_), respectively. **Zn-L-Cs** was a one-dimensional coordination polymer [ZnCs­(OC_10_H_5_(COOC_2_H_5_)_2_)_3_]_*n*_. In Co^2+^ and Ni^2+^, the formation of similar coordination polymers was suggested by MS, FTIR, and EDX. In UV–Vis spectroscopy, the maximum absorption peak of DEHA was observed at 451 nm, whereas that of **Metals-L** was observed at ∼ 458 nm. This red shift is due to the change in the energy level caused by the conjugated system and complexation, and to a more favorable S_0_–S_1_ transition. DEHA and **Zn-L-Cs** exhibited a fluorescent emission. PMMA–**Metal-L** composite films were prepared, among which **Co-L-Cs** and **Zn-L-Cs** exhibited higher dispersibility in PMMA compared to **Pd-L**, **Cu-L**, and **Ni-L-Cs**. In heat resistance tests using TG-DTA, the results showed improved heat resistance compared to that of pure PMMA. When DEHA was reacted with Ti­(O*i*Pr)_4_ and Cs_2_CO_3_, no stable complex was formed; however, transesterification with alcohols (*i*PrOH, *n*BuOH, and allyl alcohol) proceeded in the presence of both Ti­(O*i*Pr)_4_ and Cs_2_CO_3_. Moreover, transesterification of DEAA also proceeded. The transesterification reaction is simpler and more useful than the traditional methods, and a variety of 2-hydroxyazulene and 2-aminoazulene esters can be synthesized using various alcohols. Especially, the successful introduction of allyl groups is expected to facilitate polymerization and materialization.

## Supplementary Material



## Notes

Experimental details, NMR, IR, UV–Vis, EDX, Mass spectra, TG-DTA traces, DFT calculations, and SEM images (PDF)
