# Supplementary material for: Reaction of Diethyl 2‑Hydroxyazulene-1,3-dicarboxylate with Metal Acetates and Alkoxides: Metal Complex Formation and Transesterification
Source: ACS Omega. 2025 Aug 25;10(35):39540–54. doi: 10.1021/acsomega.5c01929 (PMC12423842; doi:10.1021/acsomega.5c01929)
Supplement: Supplementary file 1 [file ao5c01929_si_004.pdf]

Supporting Information

Reaction of diethyl 2-hydroxyazulene-1,3-dicarboxylate with metal acetates and alkoxides: metal complex formation and transesterification

Tatsuya Iwashina[1,*,#], Ryohei Hayami[1,#], Yohei Sato[1], Takuya Sagawa[2], Kazuki Yamamoto[1], Takahiro Gunji[1,3,4,*]

[1] Department of Pure and Applied Chemistry, Faculty of Science and Technology, Tokyo University of Science, 2641 Yamazaki, Noda, Chiba 278-8510, Japan.

[2] Department of Industrial Chemistry, Faculty of Engineering, Tokyo University of Science, 6-3-1 Niijuku, Katsushika-ku, Tokyo, 125-8585, Japan

[3] Photocatalysis International Research Center, Tokyo University of Science, 2641 Yamazaki, Noda, Chiba 278-8510, Japan.

[4] Research Group for Advanced Energy Conversion, Research Institute for Science and Technology (RIST), Tokyo University of Science, 2641 Yamazaki, Noda, Chiba 278-8510, Japan.

* Corresponding authors E-mail: 7223701@ed.tus.ac.jp (T. Iwashina), gunji@rs.tus.ac.jp (T. Gunji)

#These authors contributed equally to this work.

Table of Contents

1. EDX spectra of **Ni-L-Cs**, **Co-L-Cs**, **Zn-L-Cs**, **Pd-L**, and **Cu-L**	3–5
2. Single crystal Structure of (troponato)palladium(II) ($Pd(Tp)_2$) and bis(2, 4-pentanedionato)-palladium(II) ($Pd(acac)_2$).	6
3. ^{1}H NMR of **Cu-L**, **Ni-L-Cs**, and **Co-L-Cs.**	7
4. Bond alternations of DEHA, **Pd-L**, and-**Zn-L-Cs** from single crystal structure.	8–10
5. Assignment of UV–Vis spectra of DEHA in CH_2Cl_2 by TD-DFT calculation.	11
6. Titration of **Zn-L-Cs** in CH_2Cl_2 adding MeOH (UV-Vis spectra).	12
7. Titration of **Zn-L-Cs** in CD_2Cl_2 adding MeOH (^{1}H NMR).	13
8. IR spectra of **Zn-L-Cs** before and after MeOH dissolution	14
9. Assignment of fluorescence of DEHA in CH_2Cl_2 by TD-DFT calculation.	15–17
10. PMMA composite films	18–24
11. Mass spectrum of compounds when mixing of DEHA, $Ti(OiPr)_4$, and Cs_2CO_3 in iPrOH	25
12. Ester-exchange reaction when mixing of DEHA with HClaq., NaOH, and Al(O*i*Pr)$_3$ in *i*PrOH.	26, 27
13. Mass spectra of **Cu-L**	28
14. DFT calculation conducted to compare thermodynamic stability between **Pd-DEAA** and **Pd-L**.	29
15. EDX spectra, NMR spectra, and TG-DTA traces	30–37
16. Experimental sections	38–42

1. EDX spectra of **Ni-L-Cs**, **Co-L-Cs**, **Zn-L-Cs**, **Pd-L**, and **Cu-L**

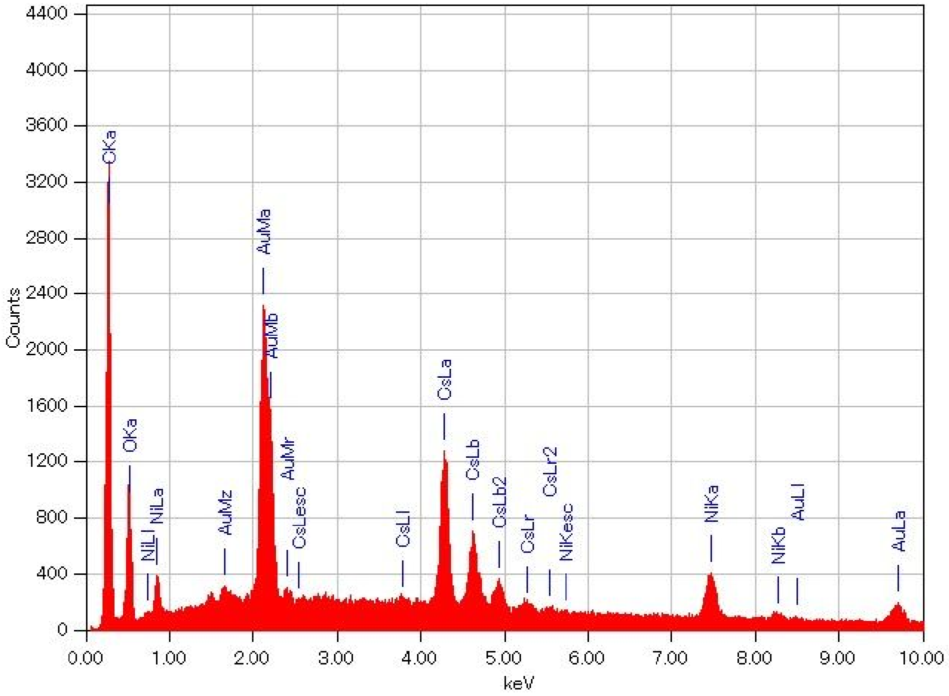

Figure S1 EDX spectra of **Ni-L-Cs**

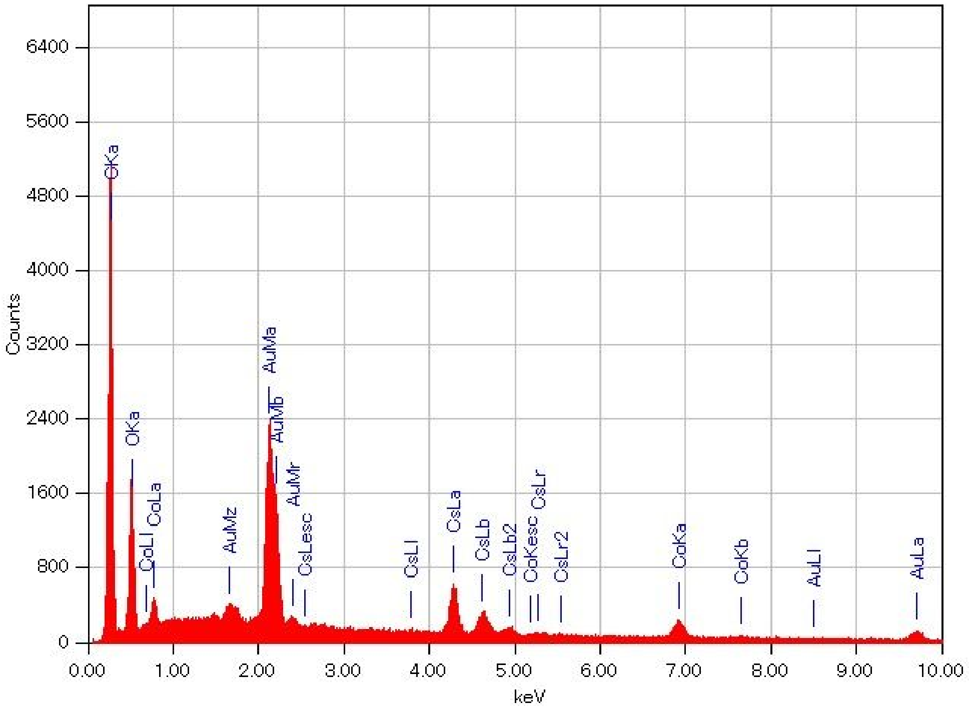

Figure S2 EDX spectra of **Co-L-Cs**

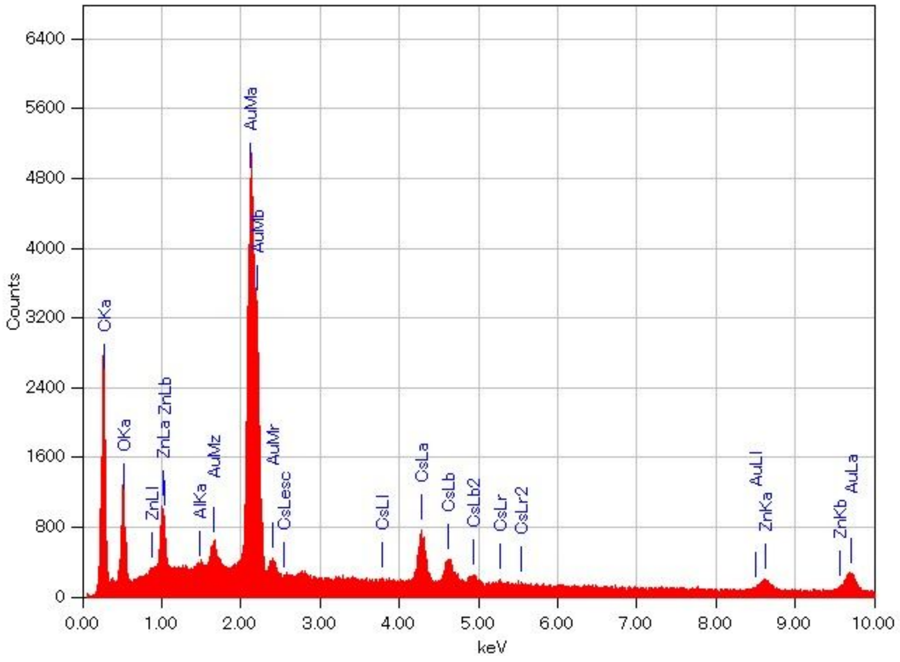

Figure S3 EDX spectra of **Zn-L-Cs**

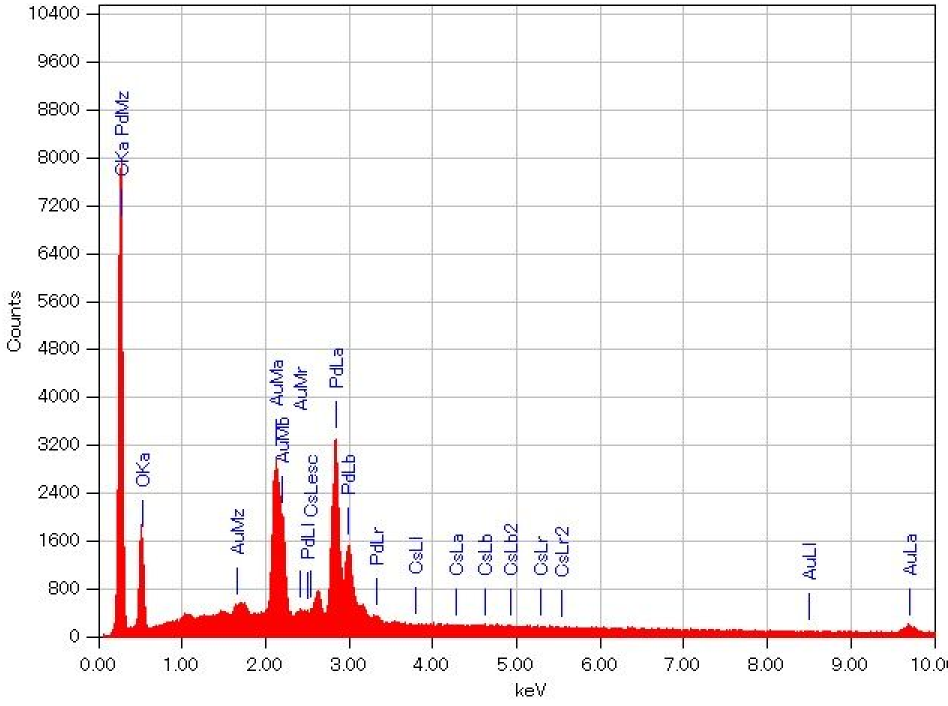

Figure S4 EDX spectra of **Pd-L**

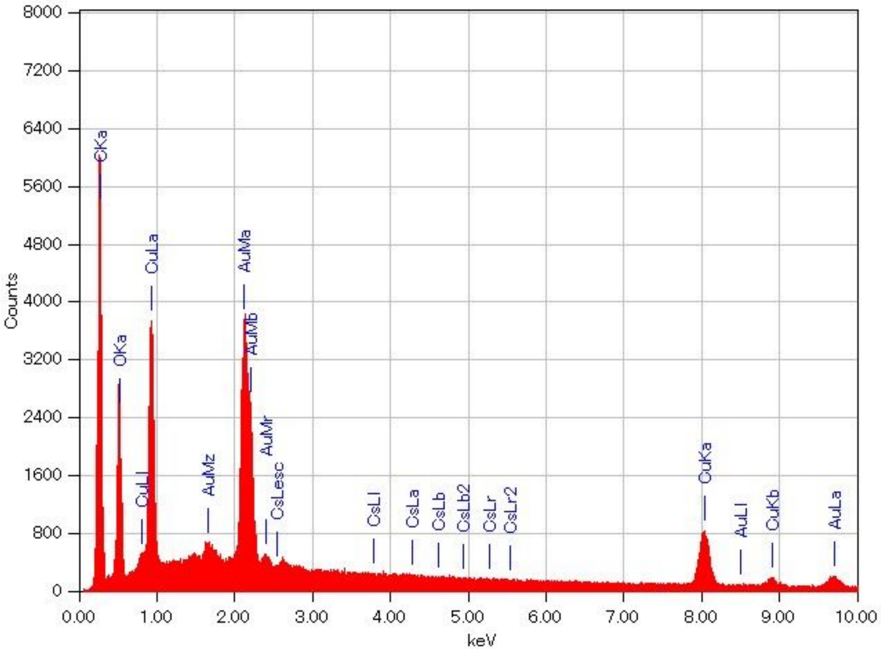

Figure S5 EDX spectra of **Cu-L**

2. Single crystal Structure of (troponato)palladium(Ⅱ) (Pd(Tp)$_2$) and bis(2, 4-pentanedionato)-palladium(Ⅱ) (Pd(acac)$_2$).

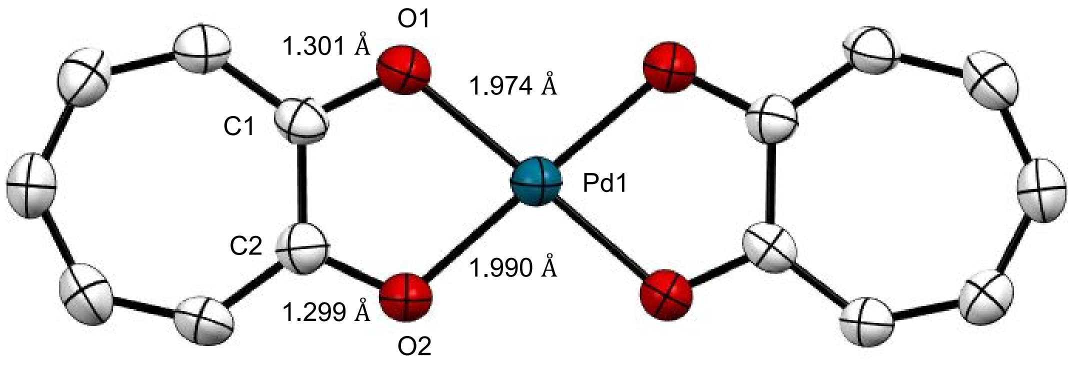

Figure S6 ORTEP drawing of Pd(Tp)$_2$ with the thermal ellipsoids shown at the 50% probability level. The hydrogen atoms are omitted for clarity; CCDC: 283792 [S1].

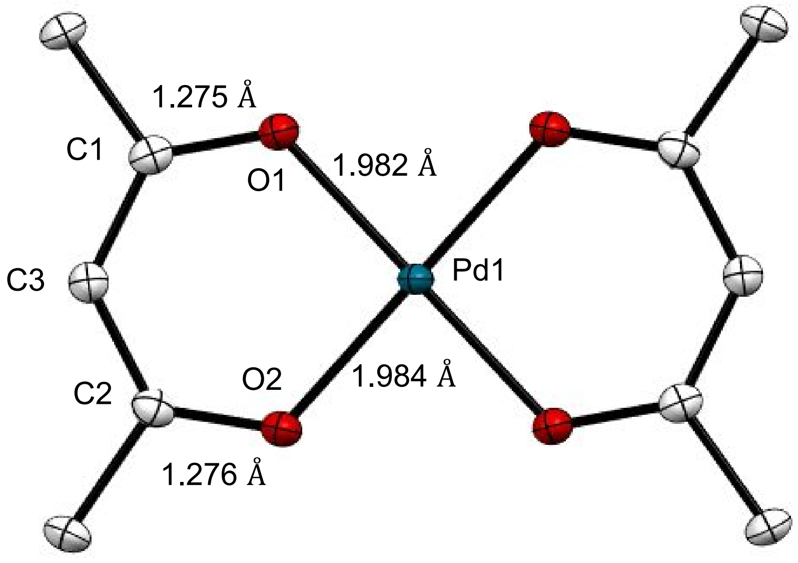

Figure S7 ORTEP drawing of Pd(acac)$_2$ with the thermal ellipsoids shown at the 50% probability level. The hydrogen atoms are omitted for clarity, CCDC: 289749 [S2].

3. ^{1}H NMR of **Cu-L, Ni-L-Cs, and Co-L-Cs.**

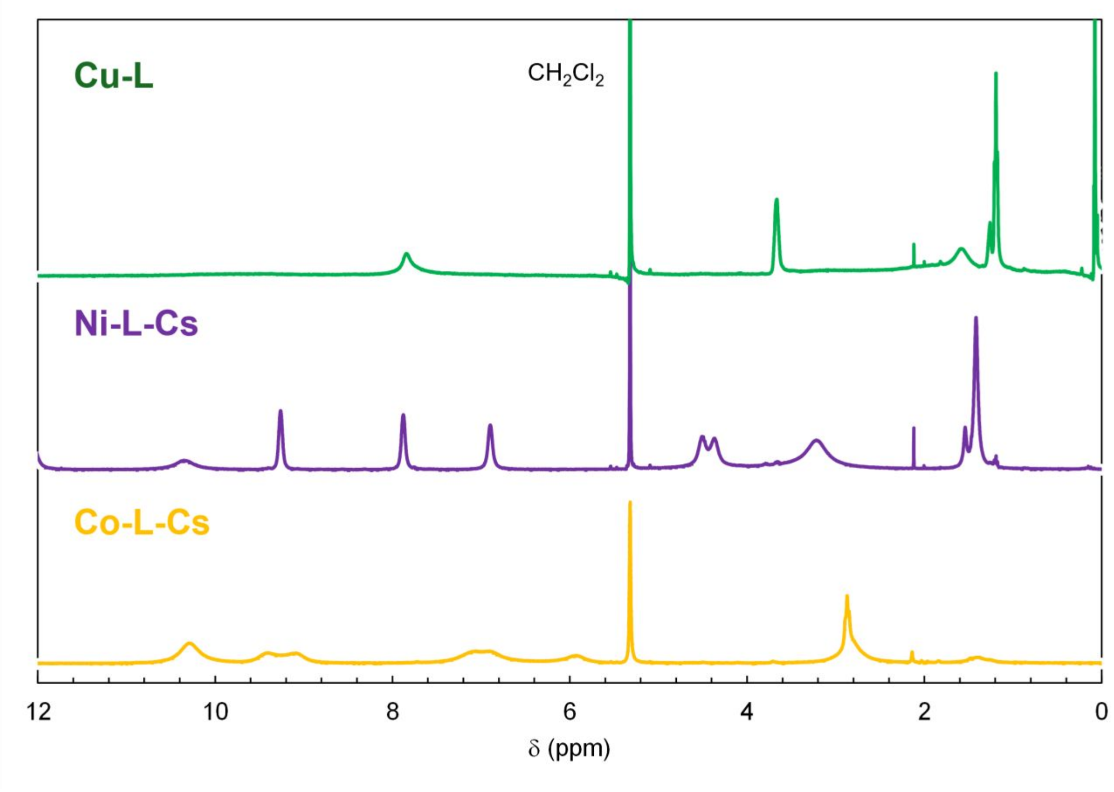

Figure S8 ^{1}H NMR spectra of **Cu-L**, **Ni-L-Cs**, and **Co-L-Cs** in CD_2Cl_2

4. Bond alternations of DEHA, **Pd-L**, and **Zn-L-Cs** from single crystal structure.

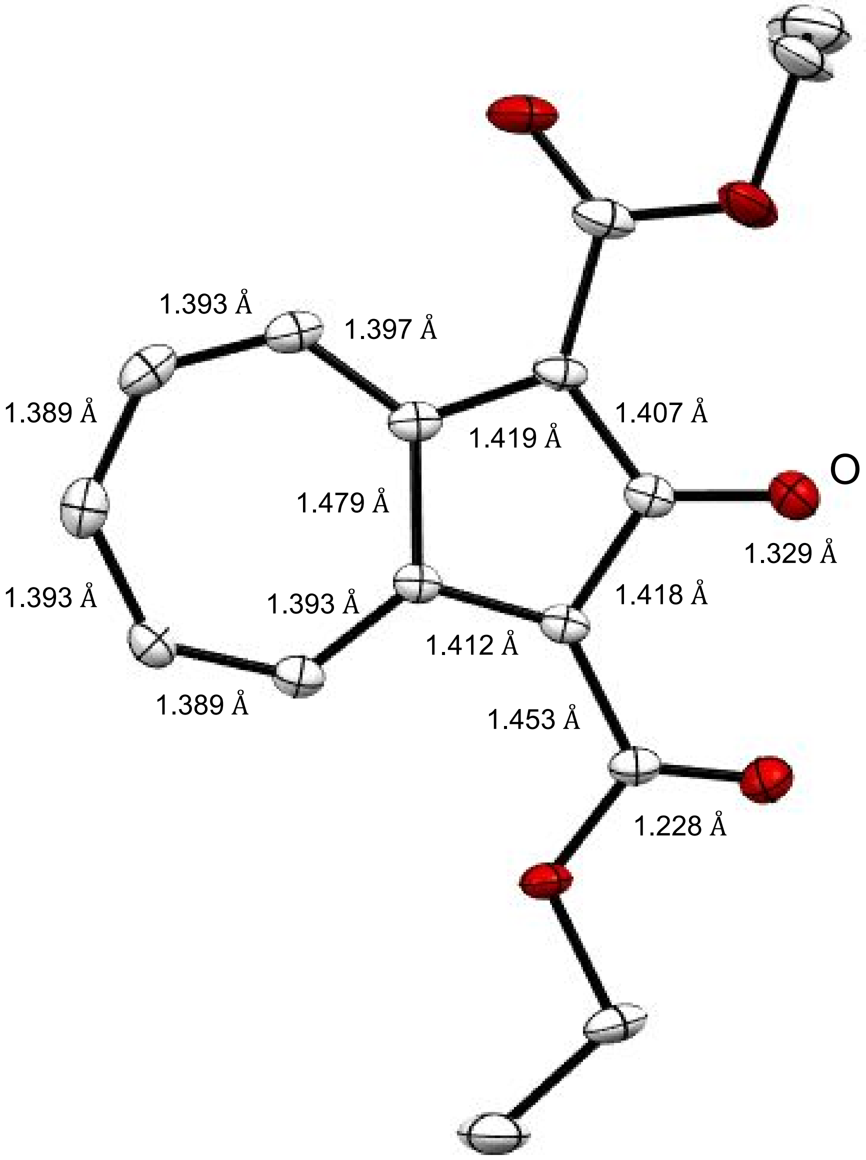

Figure S9 ORTEP drawing of DEHA with the thermal ellipsoids shown at the 50% probability level. The hydrogen atoms are omitted for clarity.

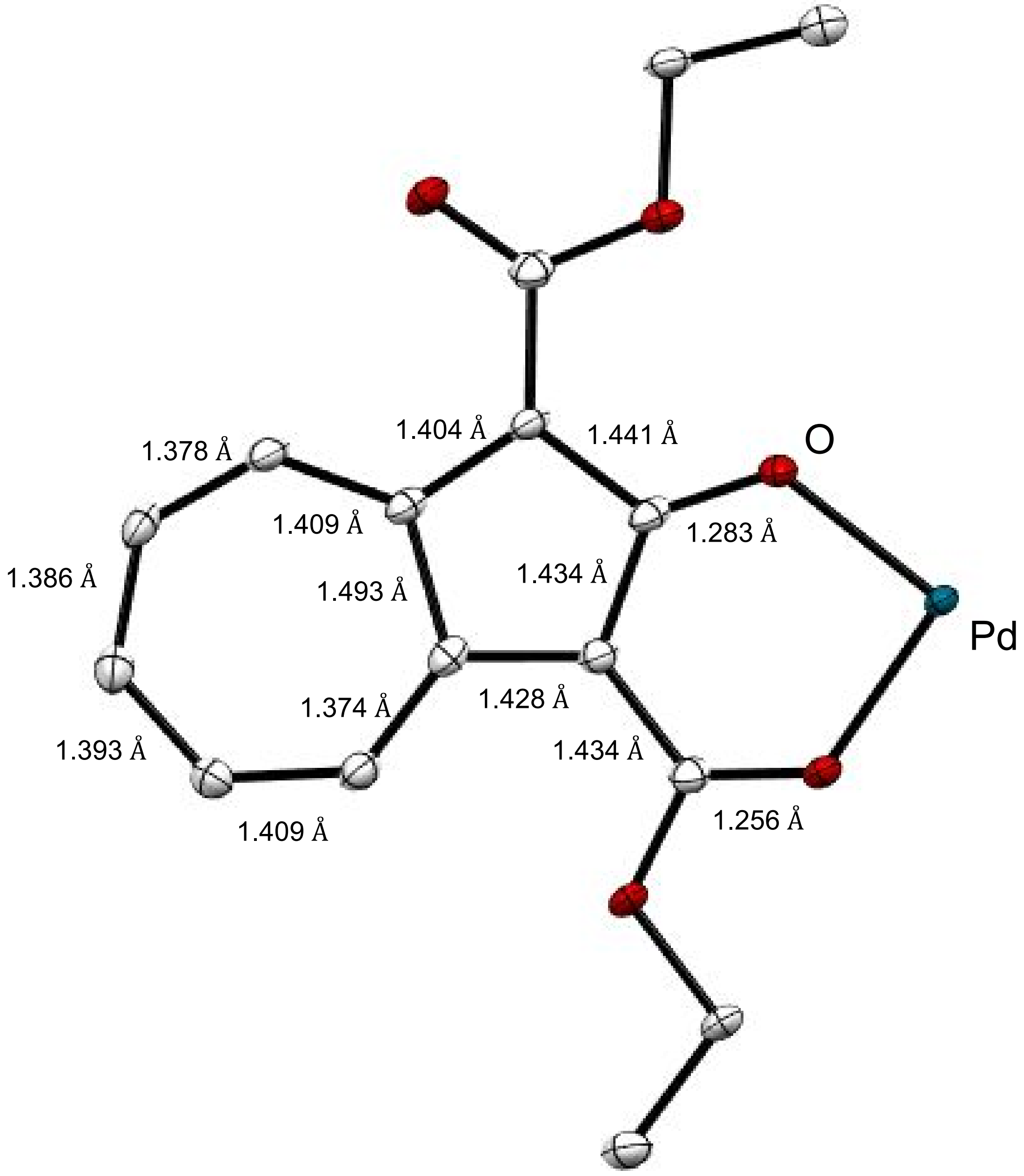

Figure S10 ORTEP drawing of **Pd-L** with the thermal ellipsoids shown at the 50% probability level. The hydrogen atoms are omitted for clarity.

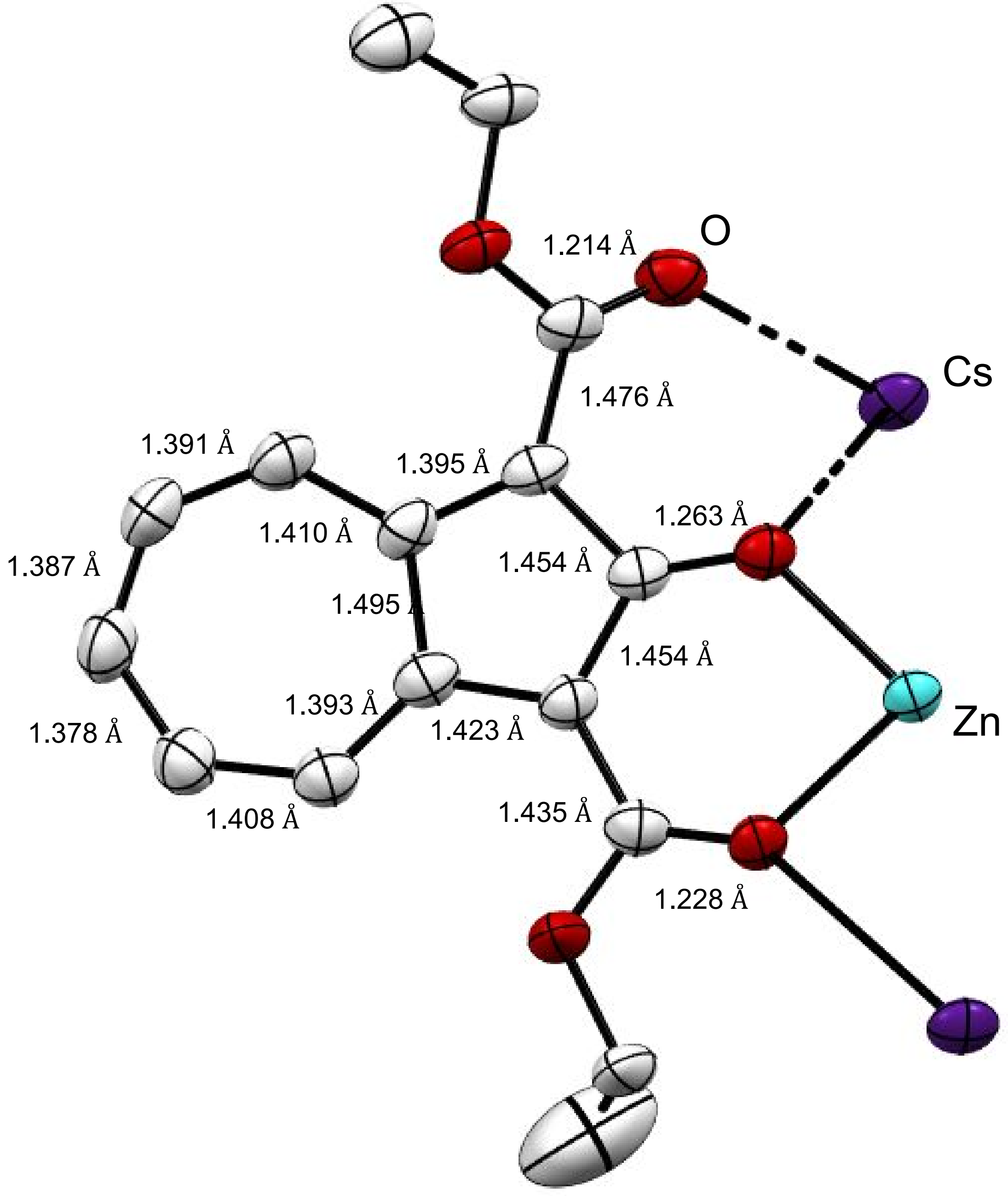

Figure S11 ORTEP drawing of **Zn-L-Cs** with the thermal ellipsoids shown at the 50% probability level. The hydrogen atoms are omitted for clarity.

5. Assignment of UV-Vis spectra of DEHA in CH_2Cl_2 by TD-DFT calculation.

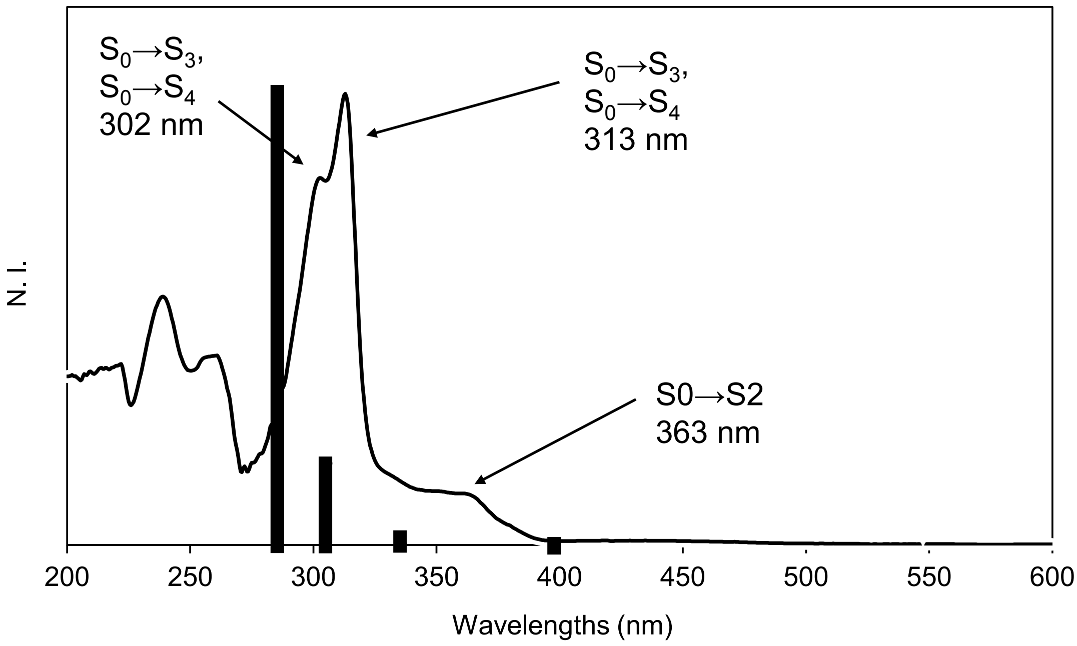

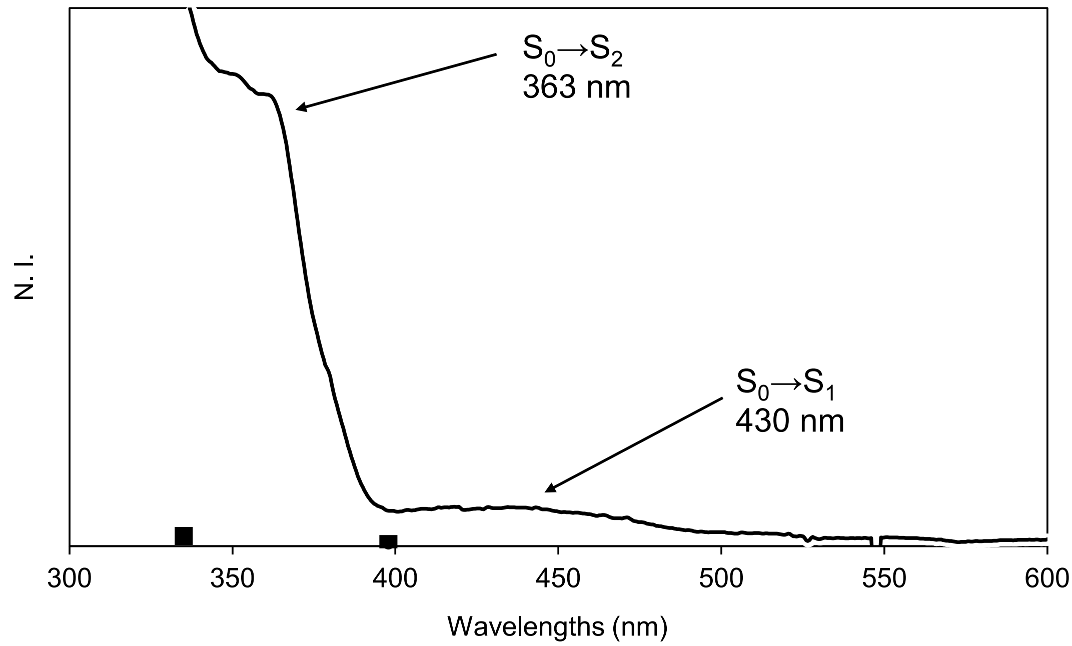

Figure S12 UV-Vis spectra of DEHA in CH_2Cl_2 and assignment by TD-DFT calculation.

6. Titration of Zn-L-Cs in CH_2Cl_2 adding MeOH (UV–Vis spectra).

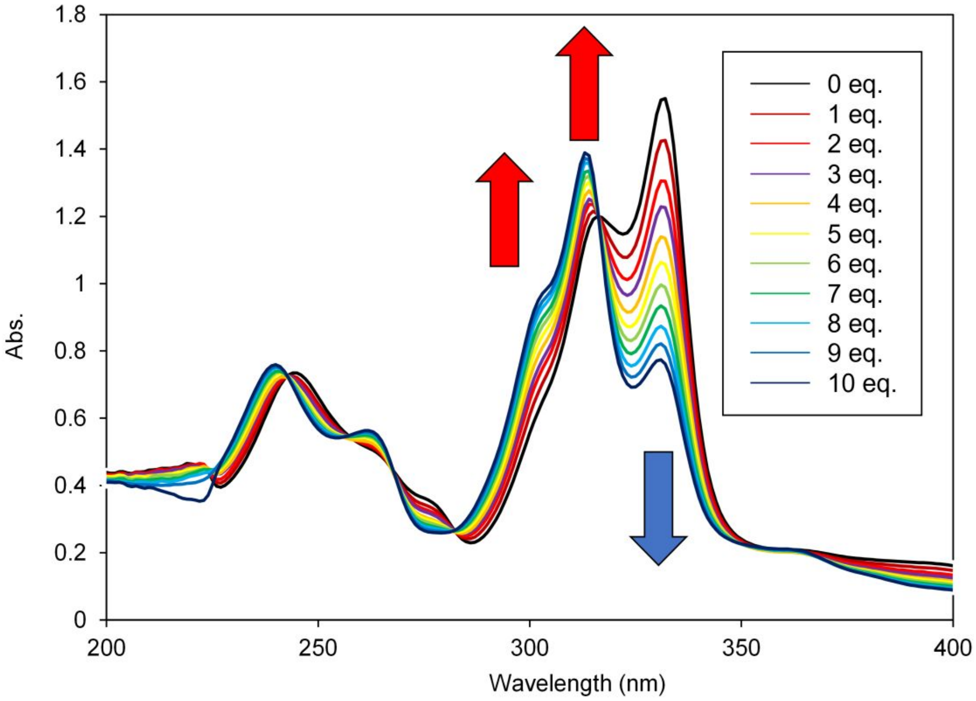

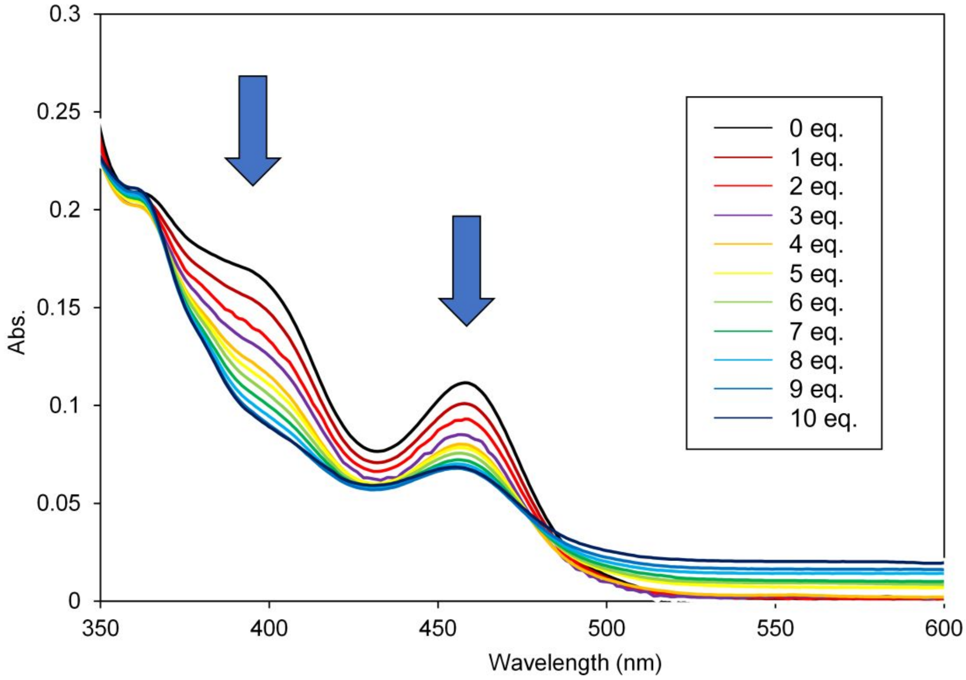

Figure S13 UV–Vis spectra of **Zn-L-Cs** in CH_2Cl_2 adding MeOH.

7. Titration of Zn-L-Cs in CD_2Cl_2 adding MeOH (^{1}H NMR).

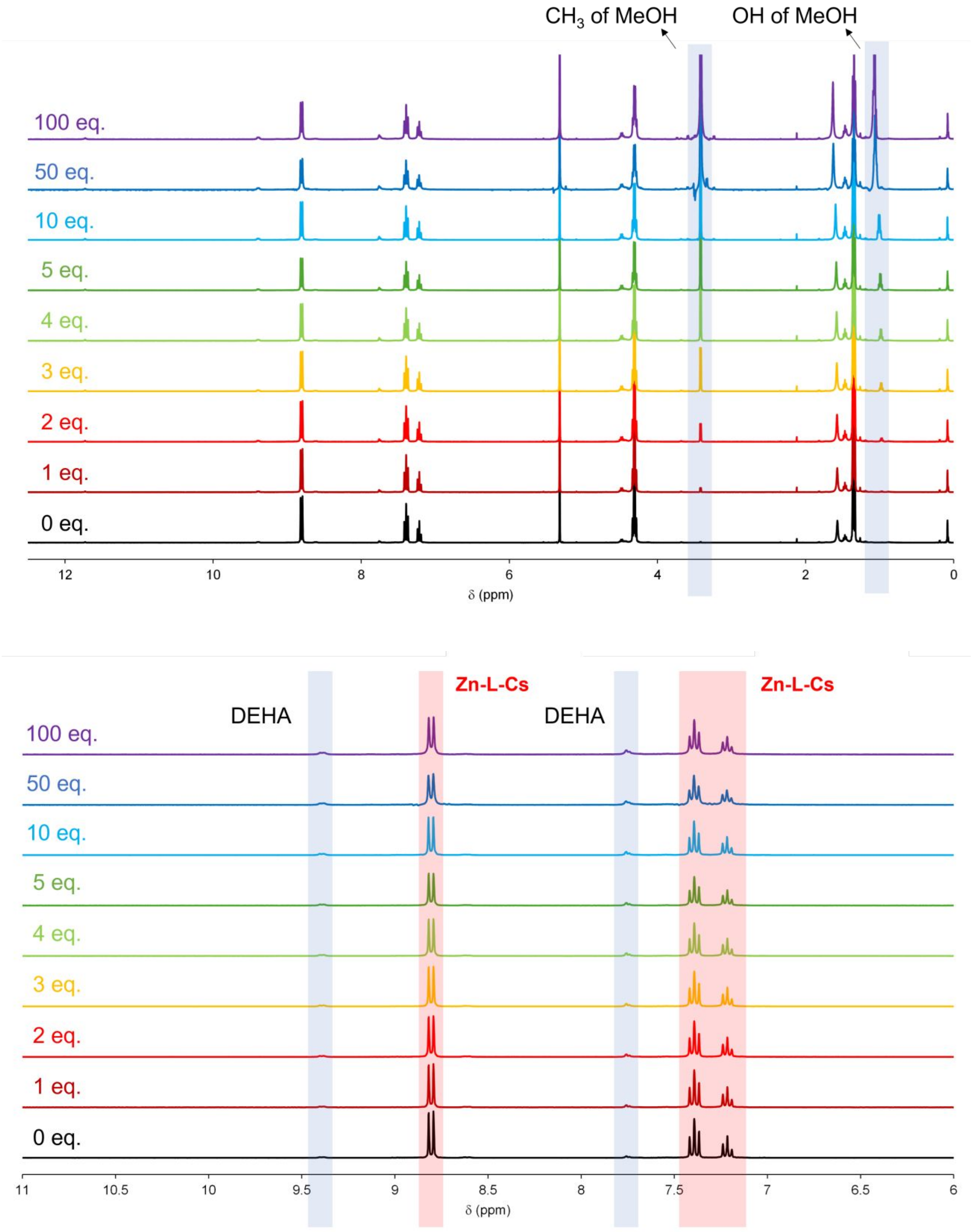

Figure S14 ^{1}H NMR spectra of **Zn-L-Cs** in CD_2Cl_2 adding MeOH.

8. IR spectra of Zn-L-Cs before and after MeOH dissolution

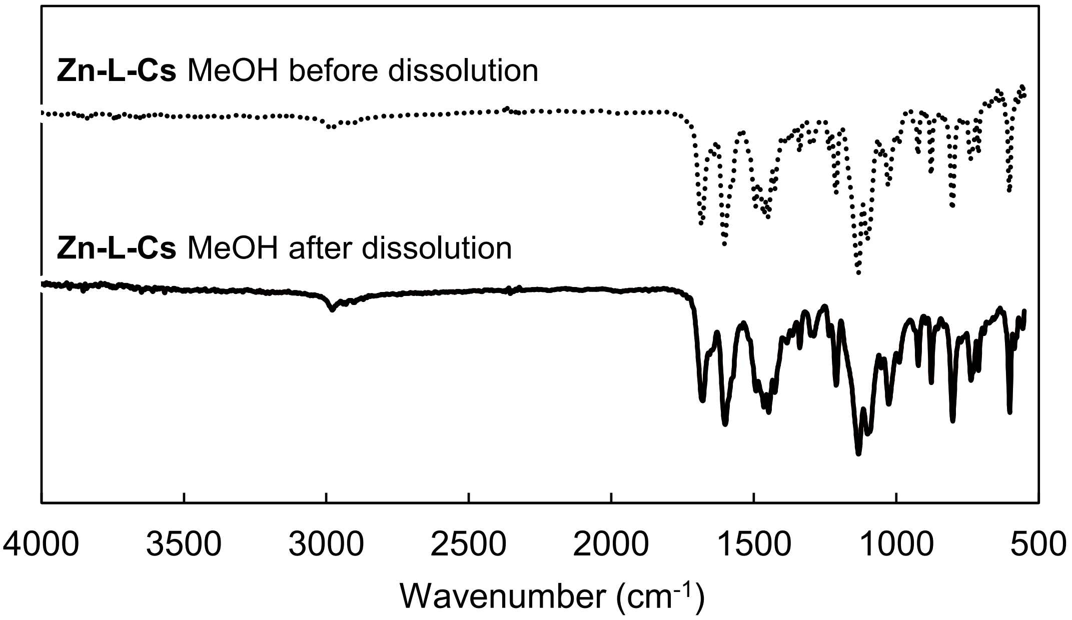

Figure S15 IR spectra of **Zn-L-Cs** before and after MeOH dissolution

9. Assignment of fluorescence of DEHA and Zn-L-Cs in CH_2Cl_2 by TD-DFT calculation.

Excited State ($S_n \to S_0$)	DEHA		
S1	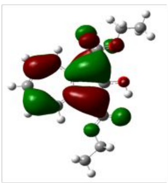<br>HOMO	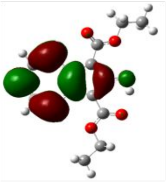<br>LUMO	2.5247 eV<br>f = 0.0026<br>491 nm<br>HOMO (H)→LUMO (L) = 0.69589
S2	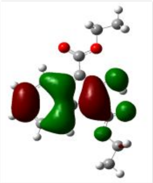<br>HOMO-1<br>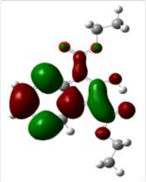<br>LUMO	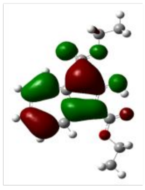<br>HOMO<br>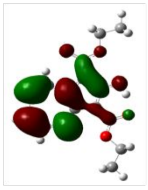<br>LUMO+1	3.3761 eV<br>f = 0.1678<br>367 nm<br>HOMO-1 (H-1)→LUMO (L) = -0.26316<br>HOMO-1 (H-1)→LUMO+1 (L+1) = -0.13385<br>HOMO (H)→LUMO (L) = -0.56668<br>HOMO (H)→LUMO+1 (L+1) = 0.29341

Figure S16 Assignment of fluorescence of DEHA in CH_2Cl_2 by TD-DFT calculation.

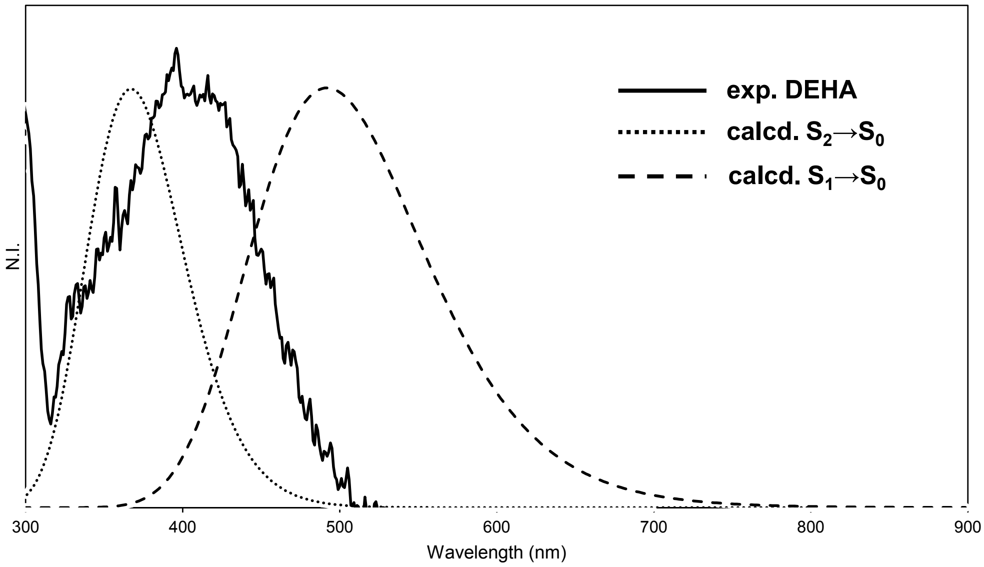

Figure S17 Fluorescence spectra of exp. DEHA in CH_2Cl_2 and calcd. DEHA in CH_2Cl_2 by TD-DFT calculation.

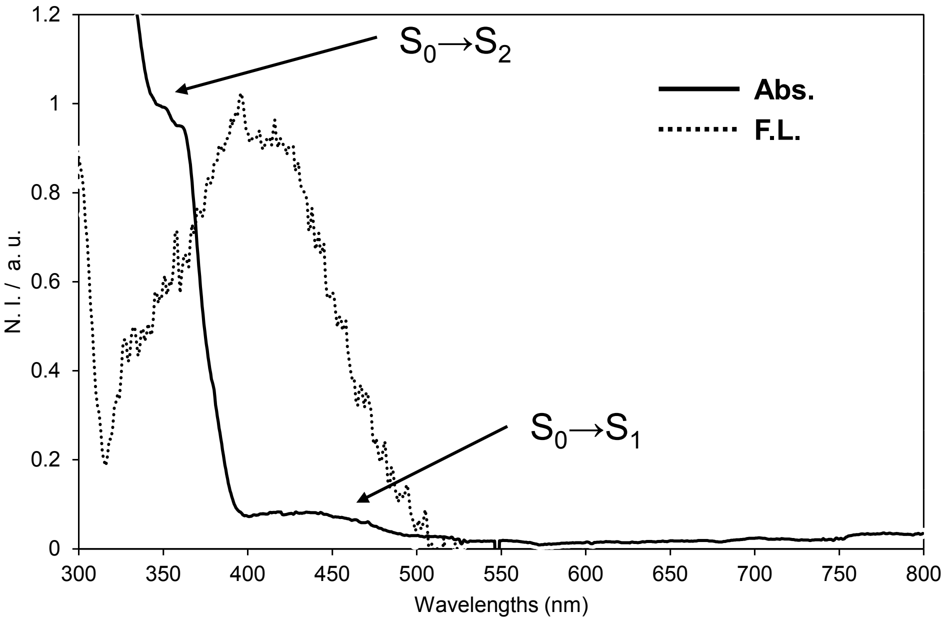

Figure S18 UV-Vis spectra and fluorescence (F.L.) spectra DEHA in CH_2Cl_2.

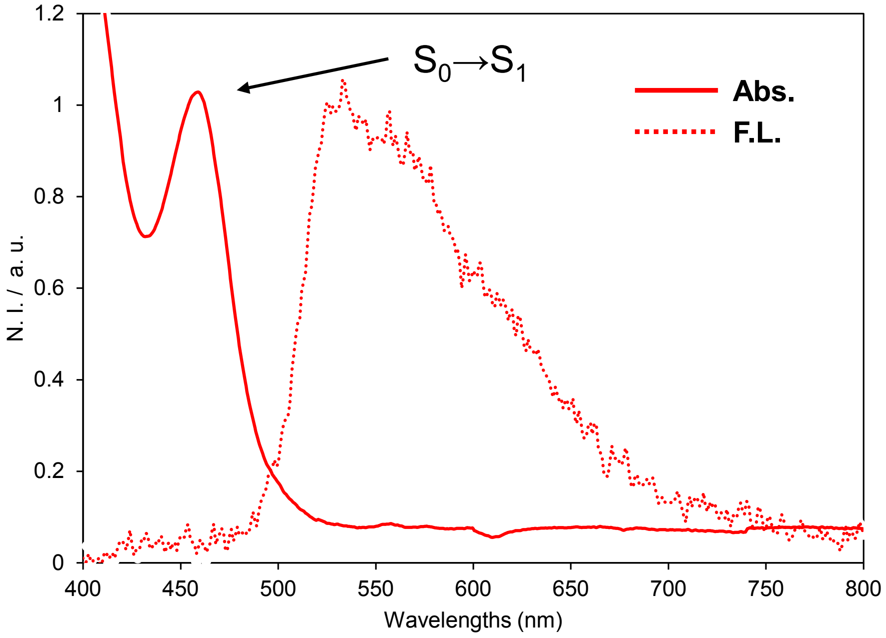

Figure S19 UV-Vis spectra and fluorescence (F.L.) spectra **Zn-L-Cs** in CH_2Cl_2.

10. PMMA composite films

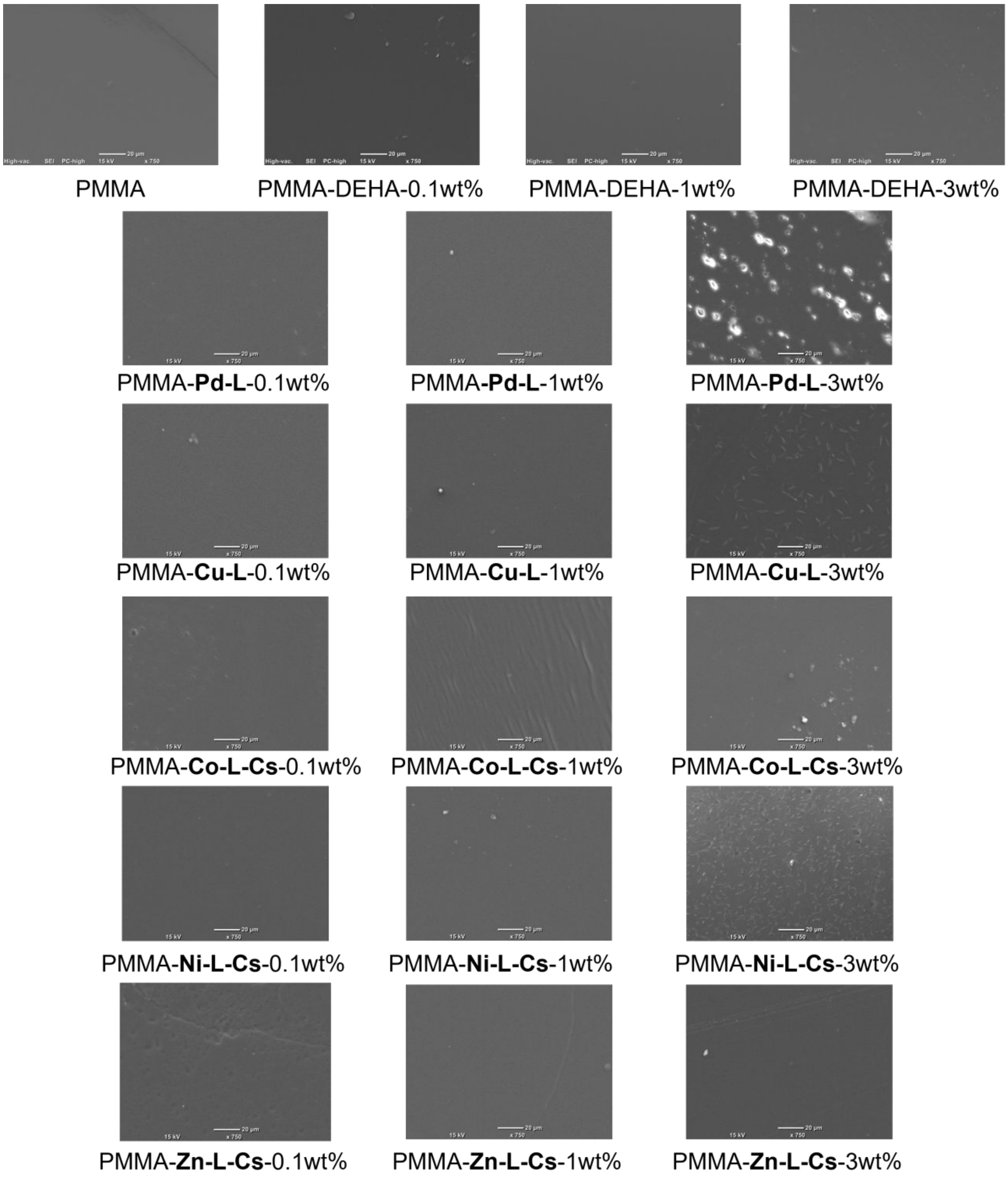

Figure S20 SEM images of PMMA composite films

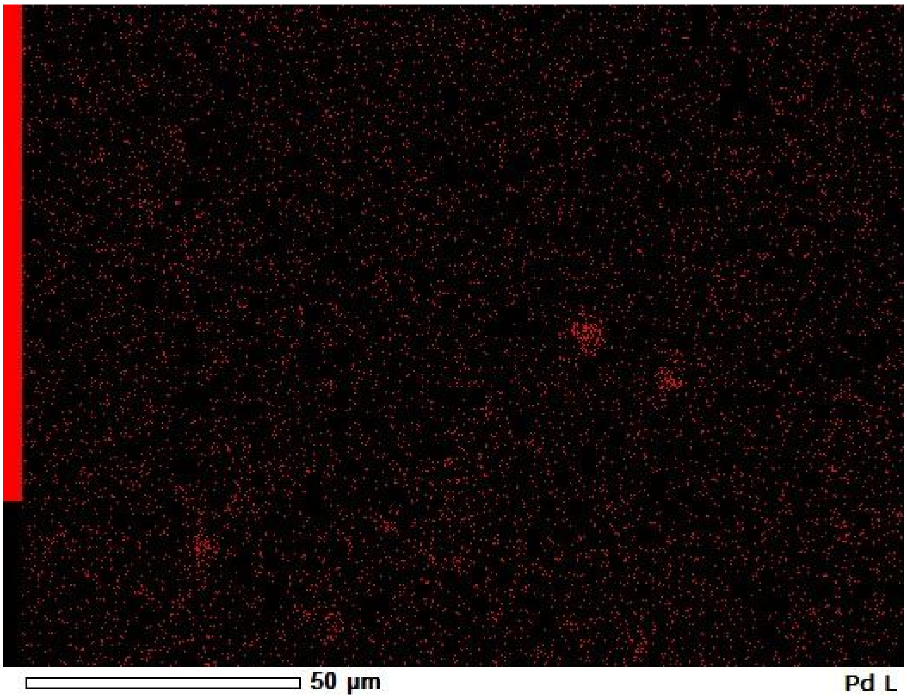

Figure S21 Elemental mapping of PMMA-Pd-L-3wt%

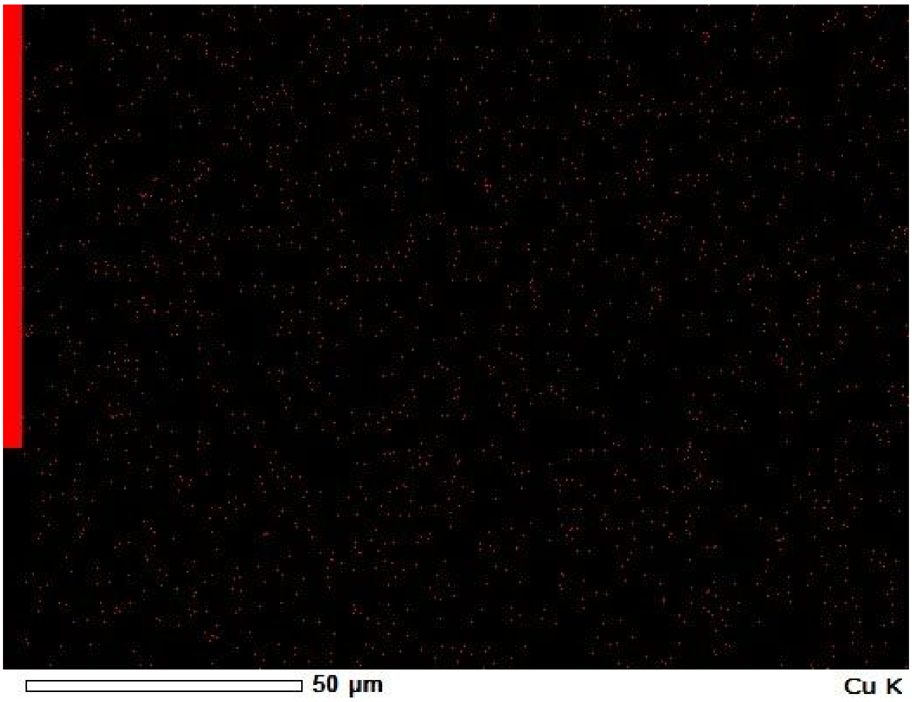

Figure S22 Elemental mapping of PMMA-Cu-L-3wt%

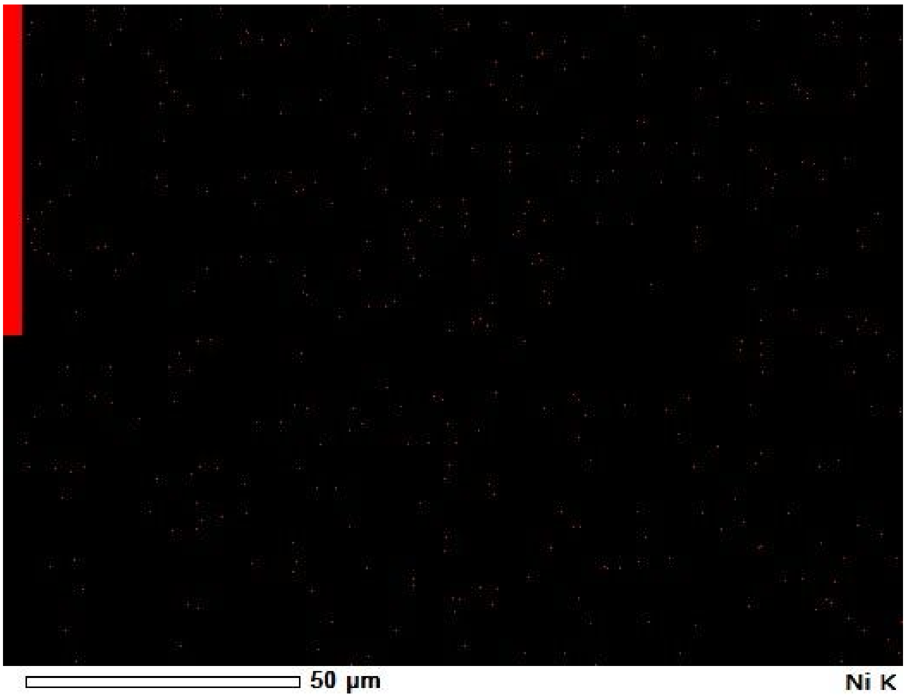

Figure S23 Elemental mapping of PMMA-Ni-L-Cs-3wt%

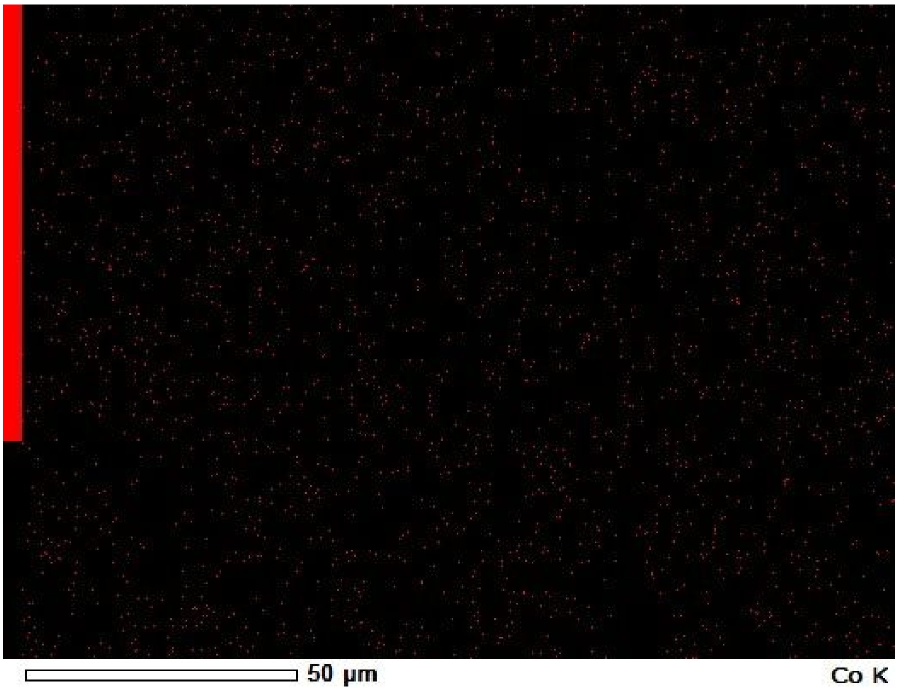

Figure S24 Elemental mapping of PMMA-Co-L-Cs-3wt%

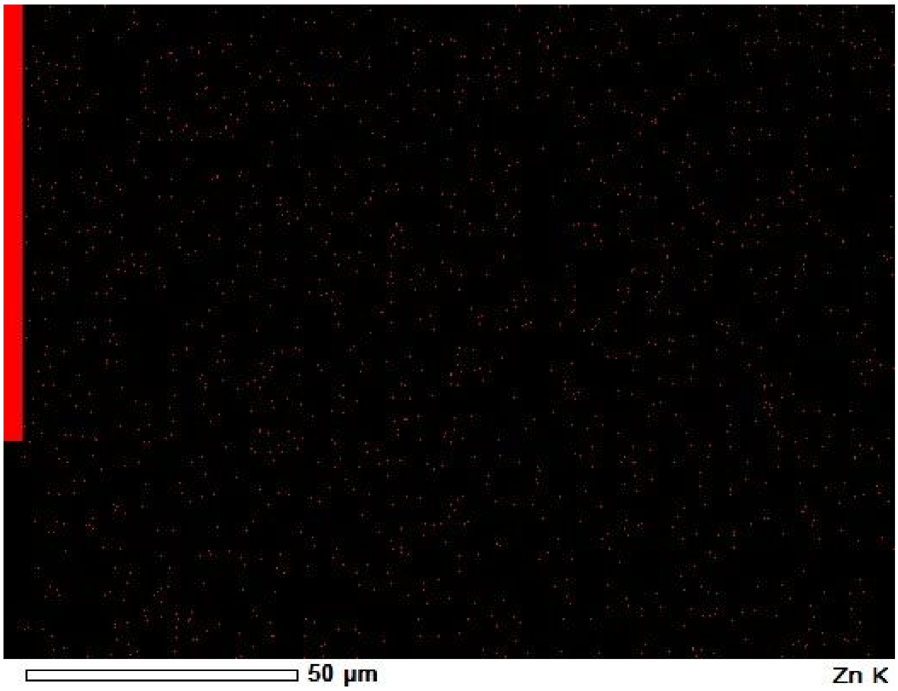

Figure S25 Elemental mapping of PMMA-Zn-L-Cs-3wt%

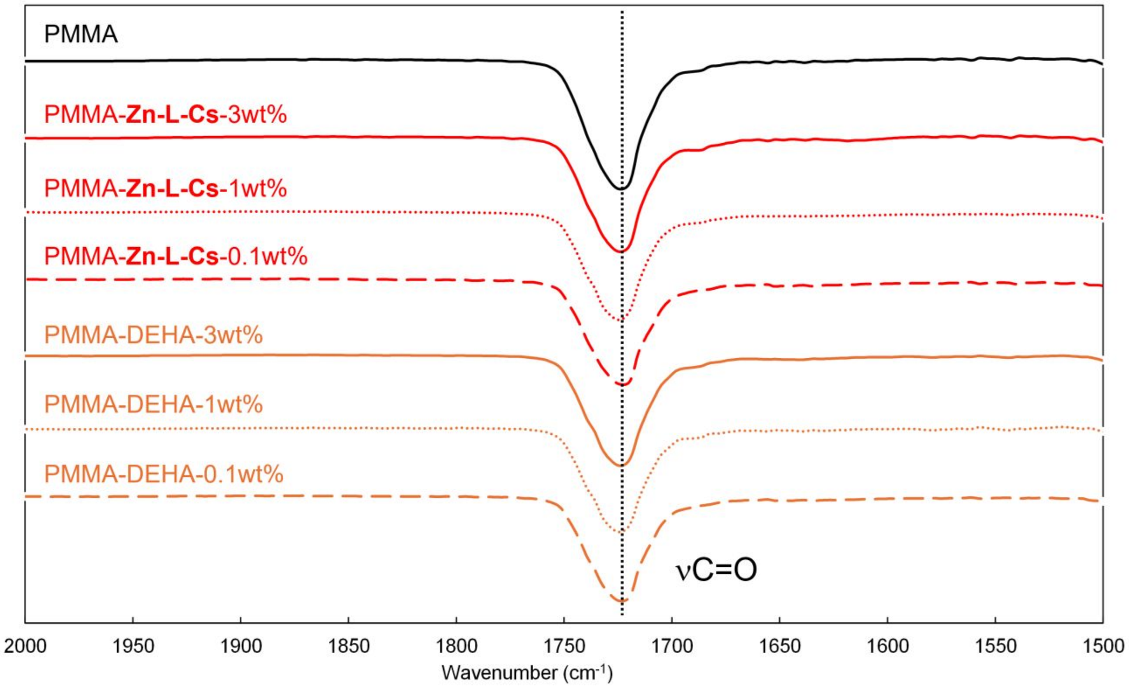

Figure S27 IR spectra of PMMA composite films (DEHA and **Zn-L-Cs**)

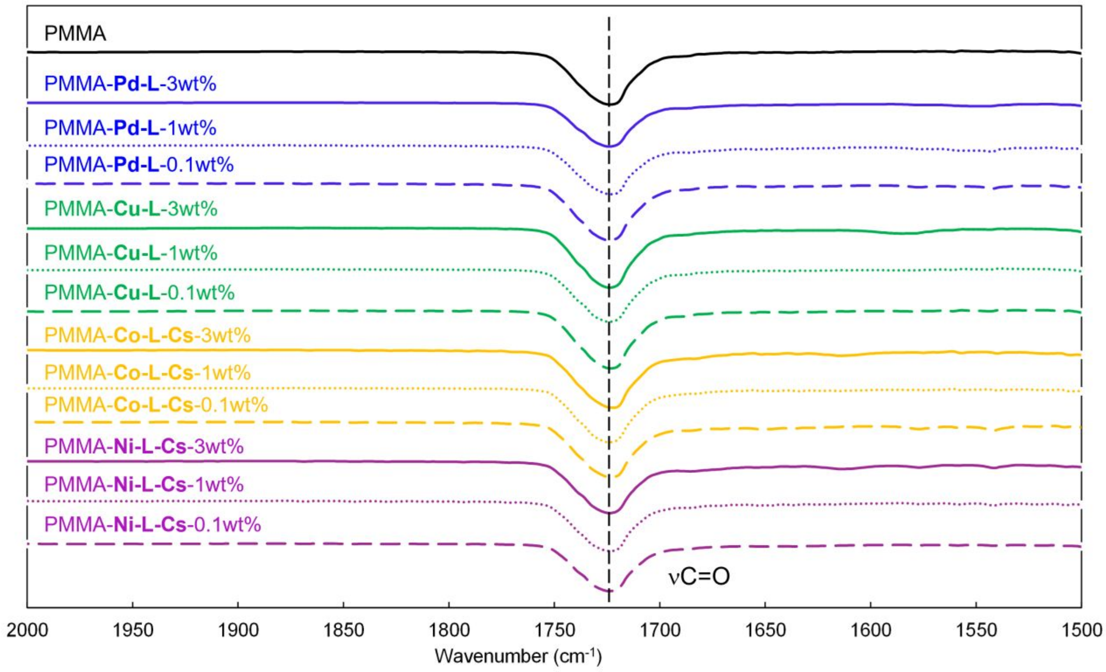

Figure S28 IR spectra of PMMA composite films (**Pd-L**, **Cu-L**, **Co-L-Cs**, and **Ni-L-Cs**)

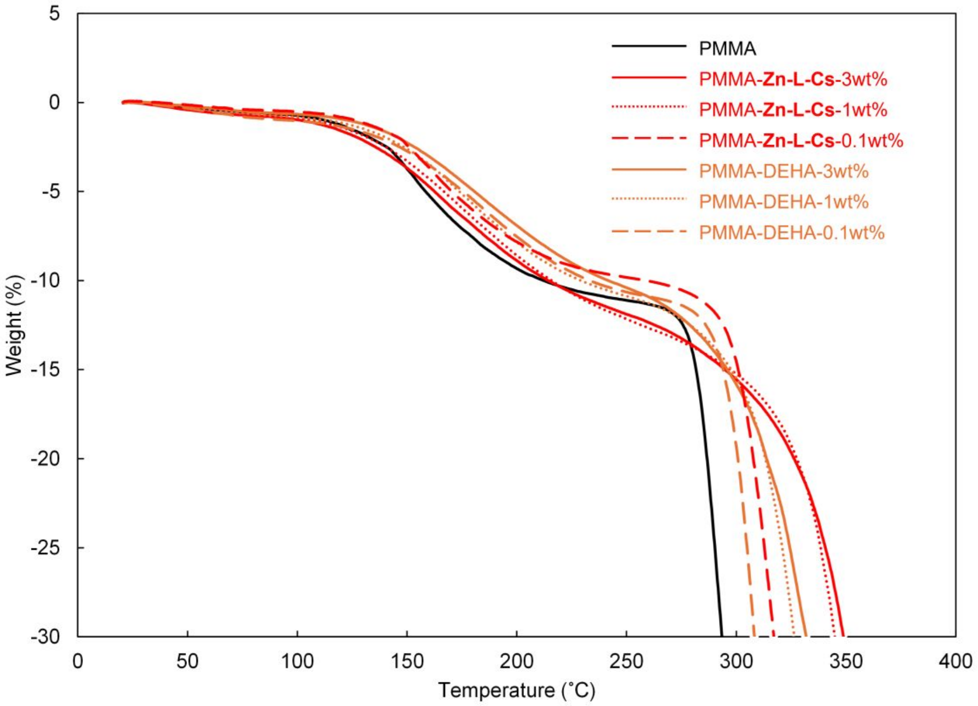

Figure S29 TG curve of PMMA composite films (DEHA and **Zn-L-Cs**)

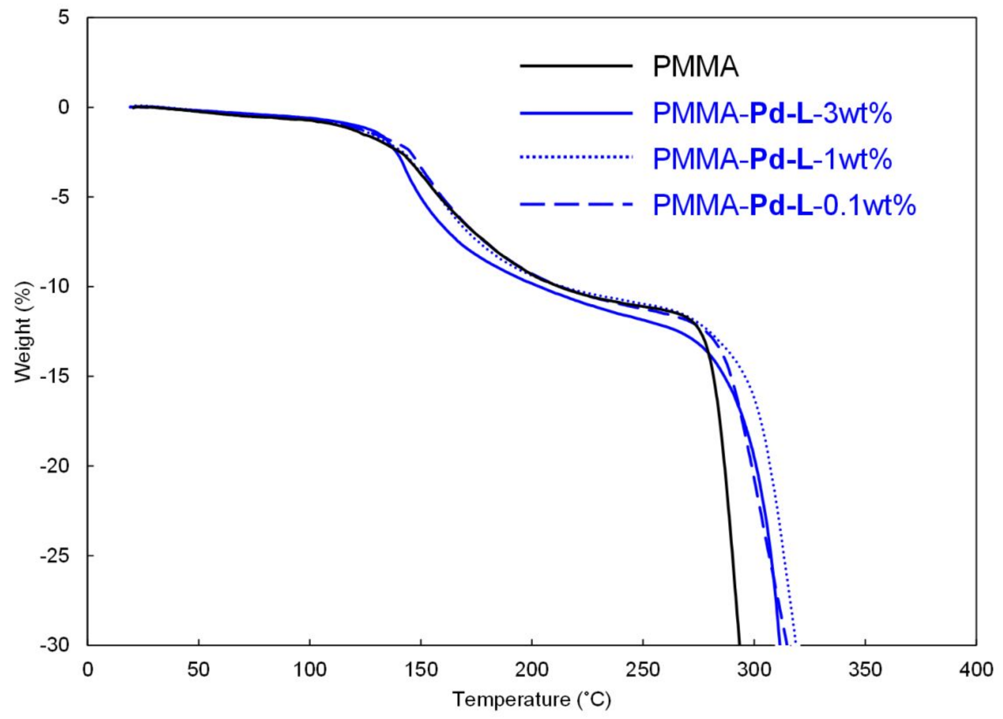

Figure S30 TG curve of PMMA-**Pd-L** composite films

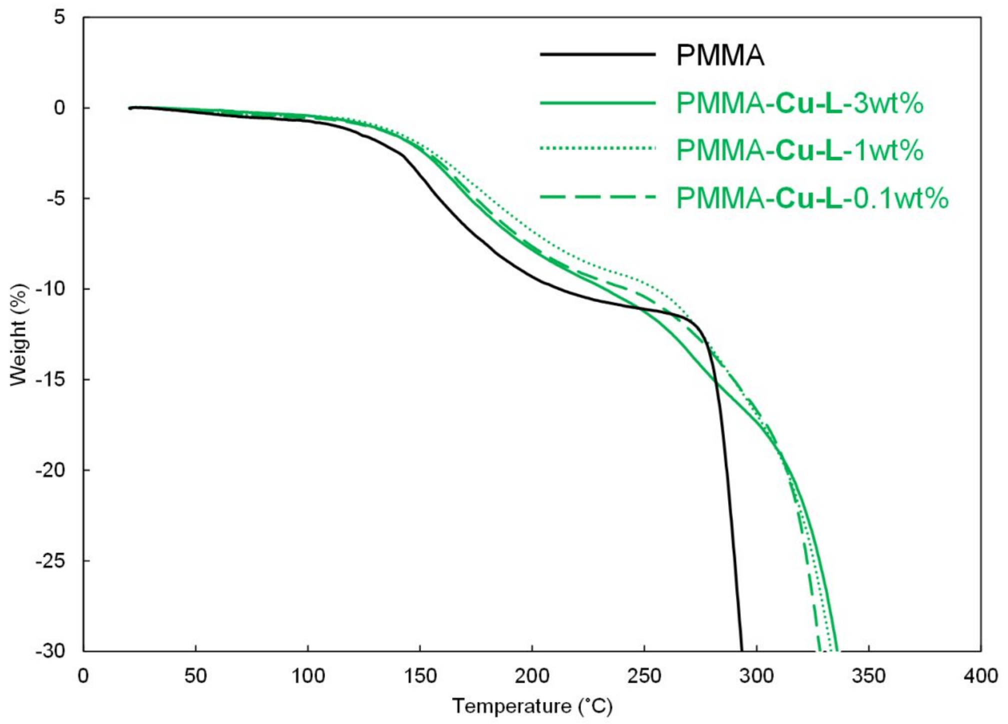

Figure S31 TG curve of PMMA-**Cu-L** composite films

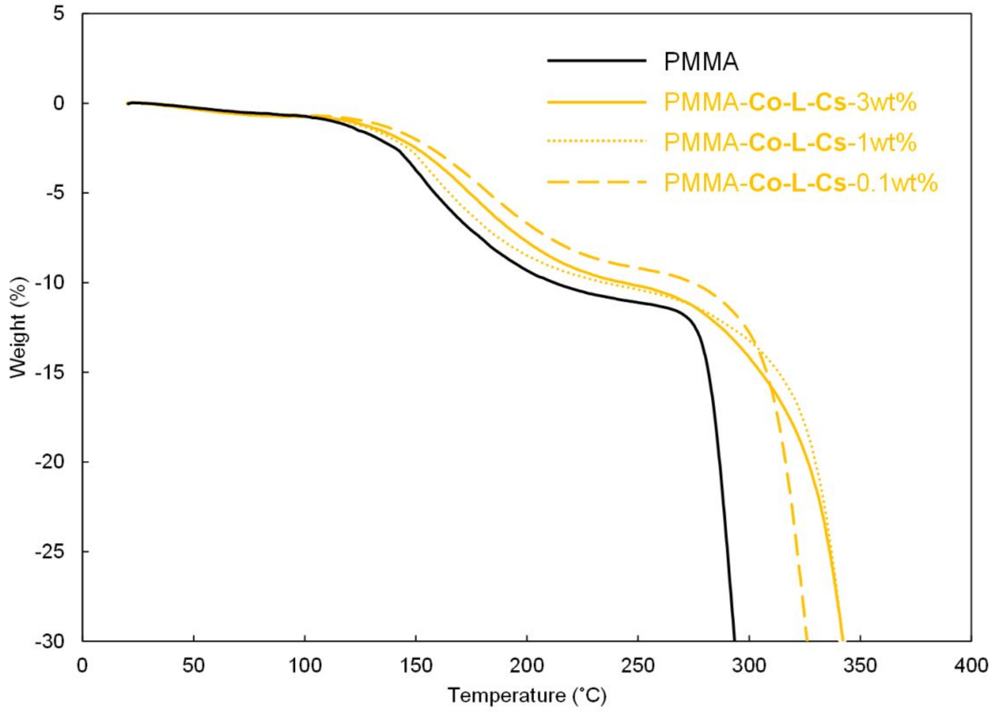

Figure S32 TG curve of PMMA-**Co-L-Cs** composite films

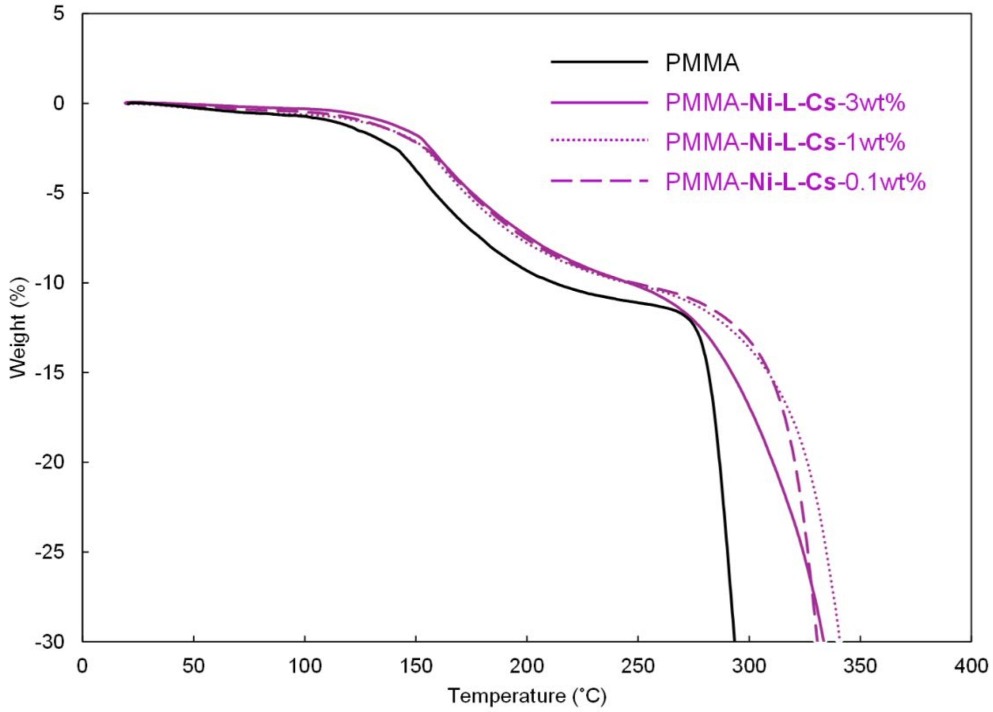

Figure S33 TG curve of PMMA-**Ni-L-Cs** composite films

11. Mass spectrum of compounds when mixing of DEHA, Ti(O*i*Pr)$_4$, and Cs$_2$CO$_3$ in *i*PrOH

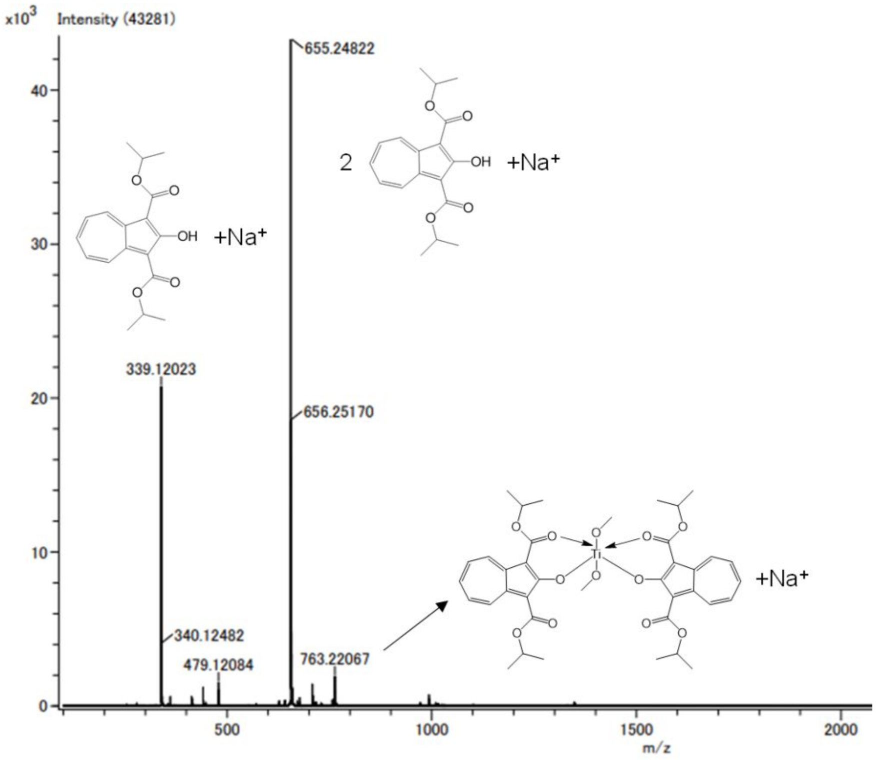

Figure S34 Mass spectrum of yellow powder formed as mixing of DEHA, Ti(O*i*Pr)$_4$, and Cs$_2$CO$_3$ in *i*PrOH followed by extraction with CHCl$_3$. Ti(D*i*PrHA)$_2$(OMe)$_2$ was observed because methanolysis of Ti(D*i*PrHA)$_2$(O*i*Pr)$_2$ proceeded using methanol as ionization source.

12. Reaction of DEHA with HClaq., NaOH, or Al(O*i*Pr)$_3$ in *i*PrOH

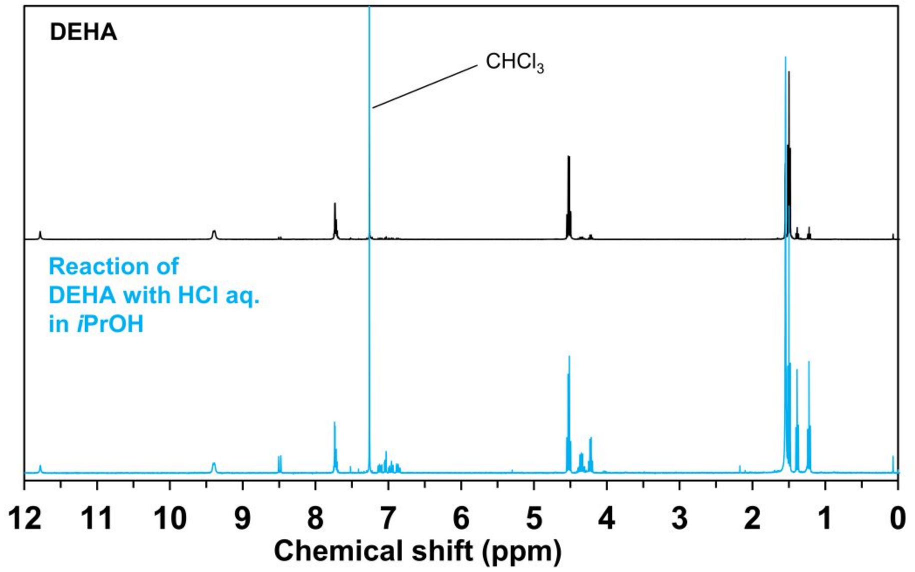

Figure S35 ^{1}H NMR spectra in $CDCl_3$ of DEHA and reaction product of DEHA and HCl aq. in *i*PrOH

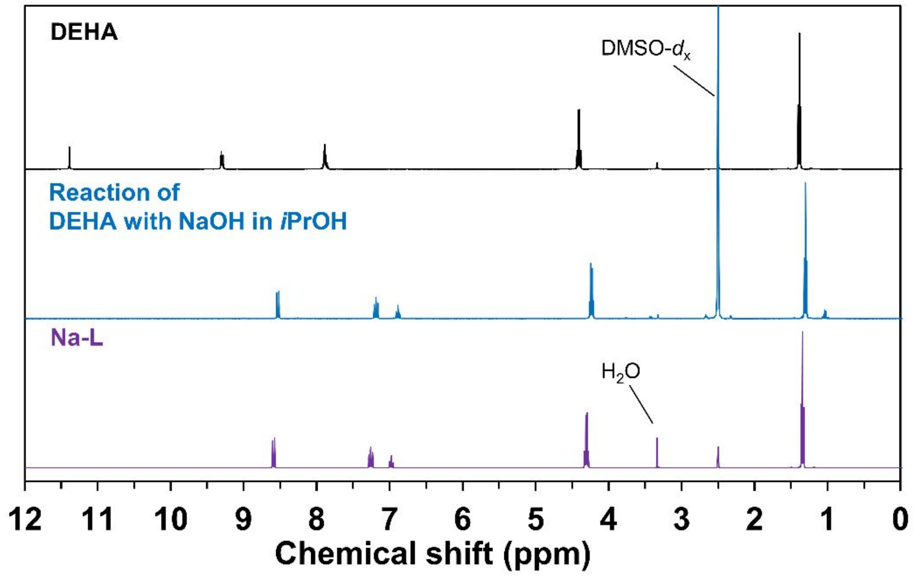

Figure S36 ^{1}H NMR spectra in DMSO-d_6 of DEHA, reaction product of DEHA and NaOH in *i*PrOH , and isolated **Na-L**

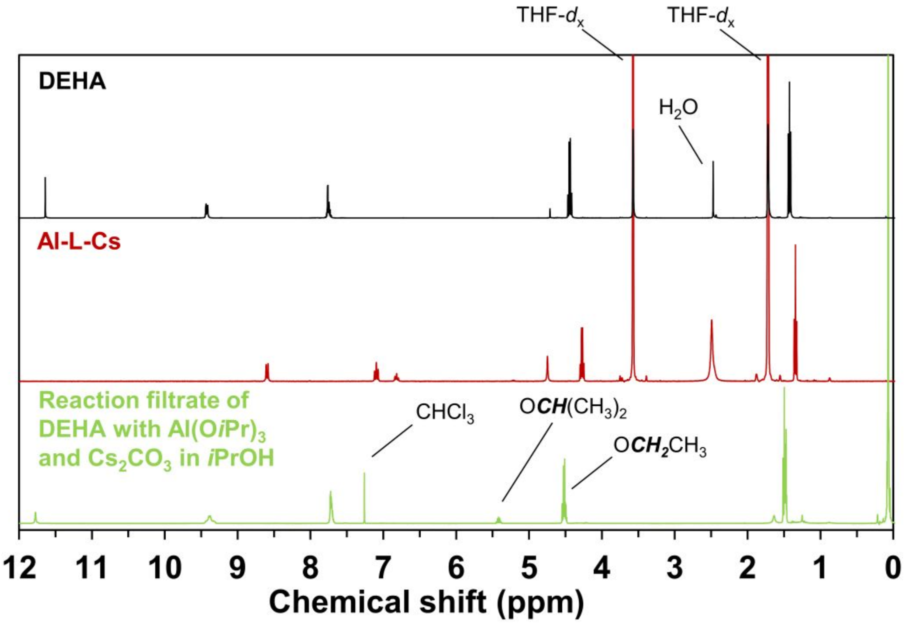

Figure S37 ^{1}H NMR spectra of DEHA and **Al-L-Cs** in THF-d_8; reaction filtrate (in $CDCl_3$) of DEHA with Al(O*i*Pr)$_3$ and Cs_2CO_3 in *i*PrOH

13. Mass spectra of Cu-L

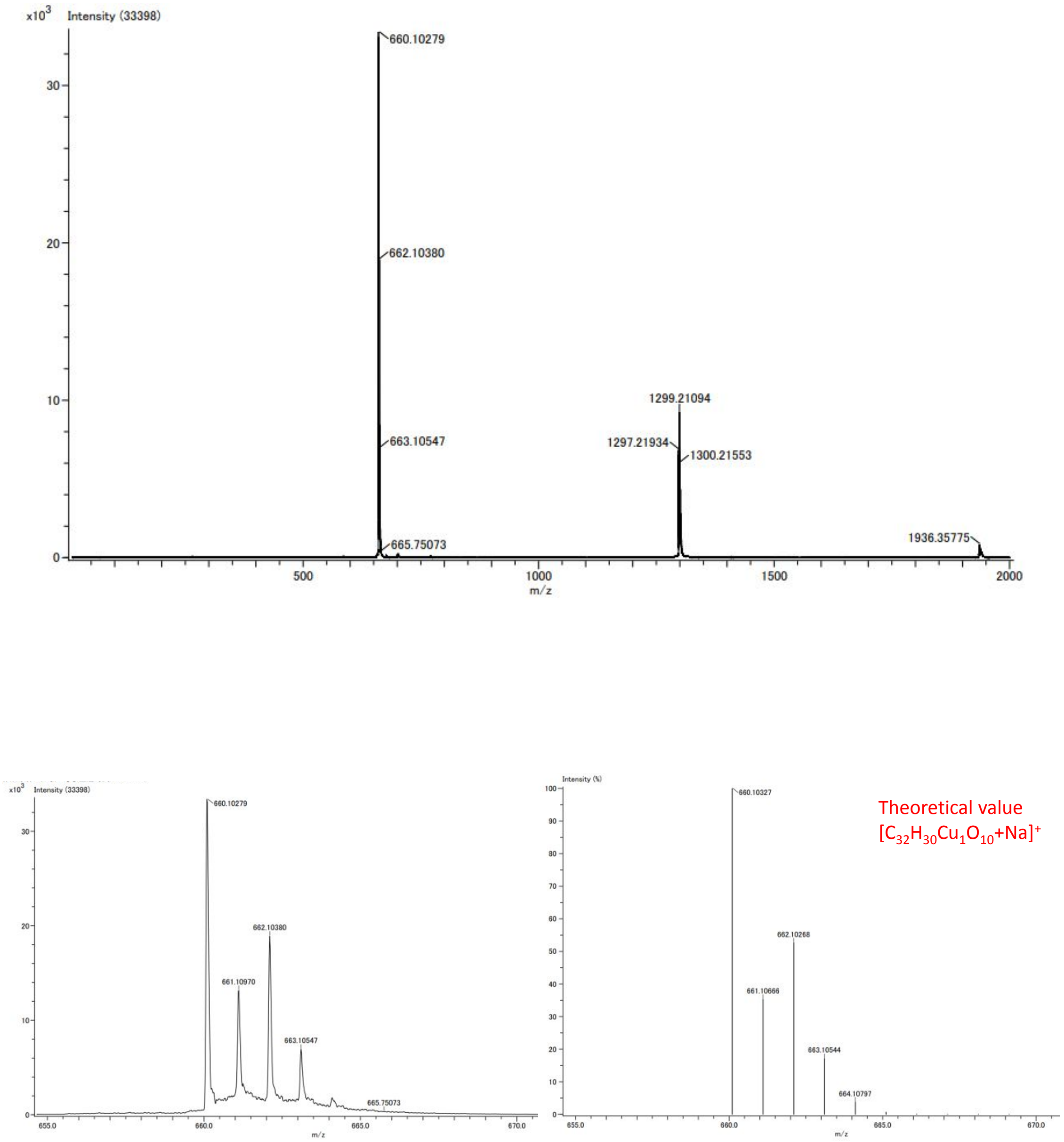

Figure S38 Mass spectra of **Cu-L**

14. DFT calculation conducted to compare thermodynamic stability between Pd-DEAA and Pd-L.

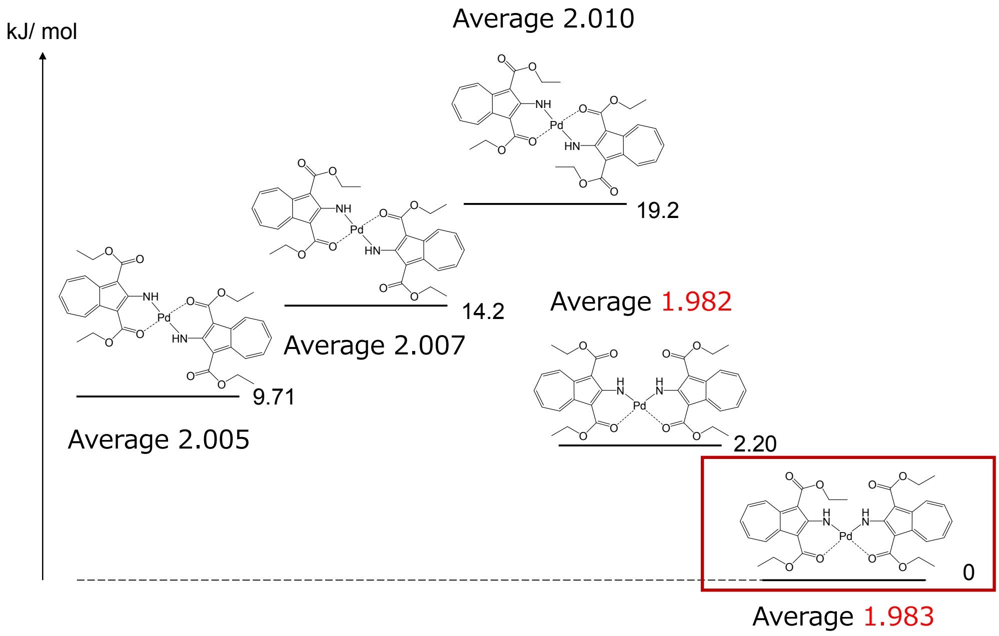

Figure S39 DFT calculation of **Pd-DEAA** and average distance of covalent Pd–N.

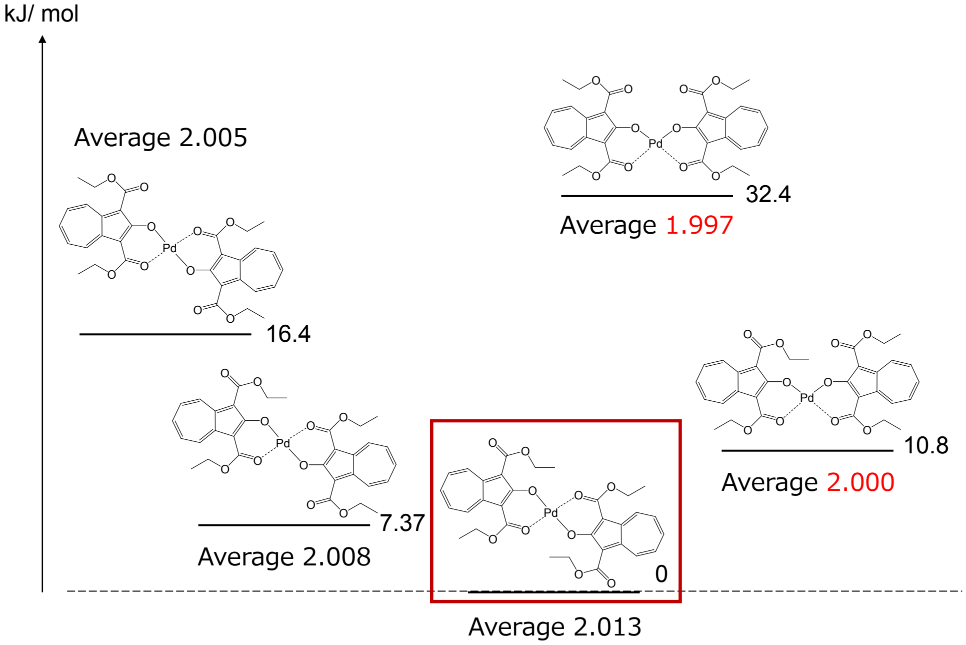

Figure S40 DFT calculation of **Pd-L** and average distance of covalent Pd–O.

15. EDX spectra, NMR spectra, and TG-DTA traces

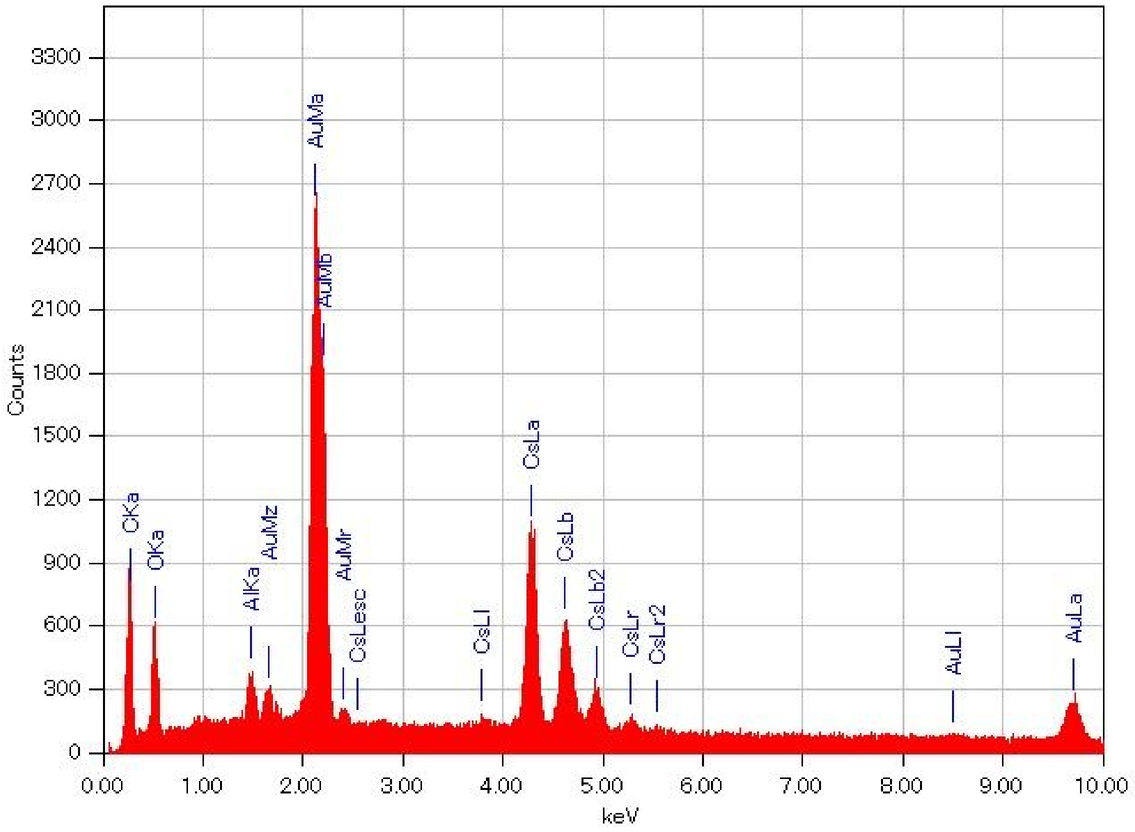

Figure S41 EDX spectra of **Al-L-Cs**

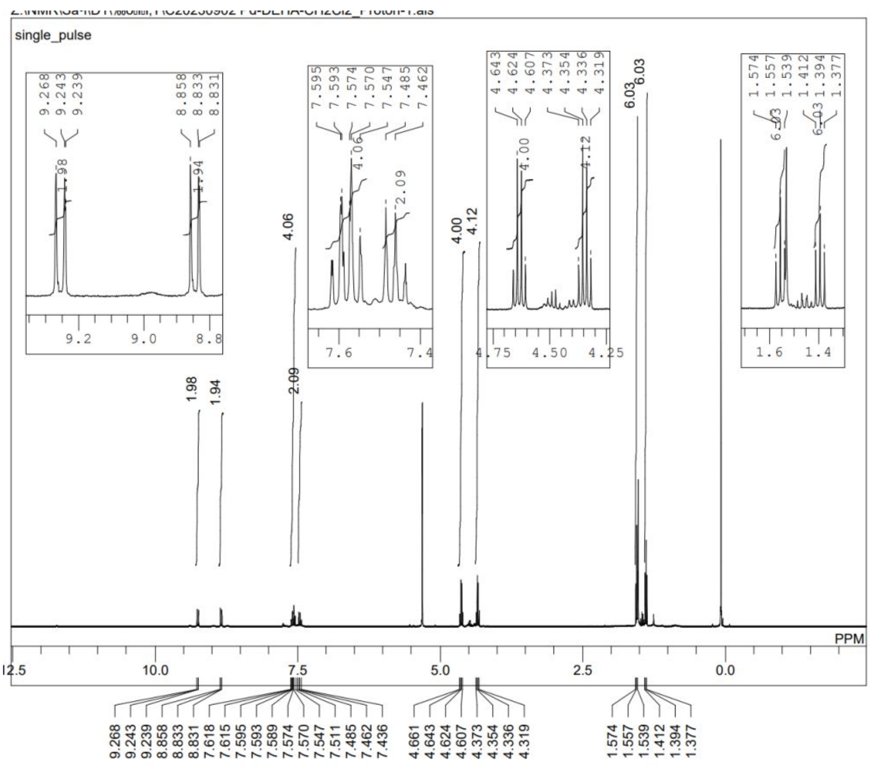

Figure S42 ^{1}H NMR of **Pd-L** in CD_2Cl_2

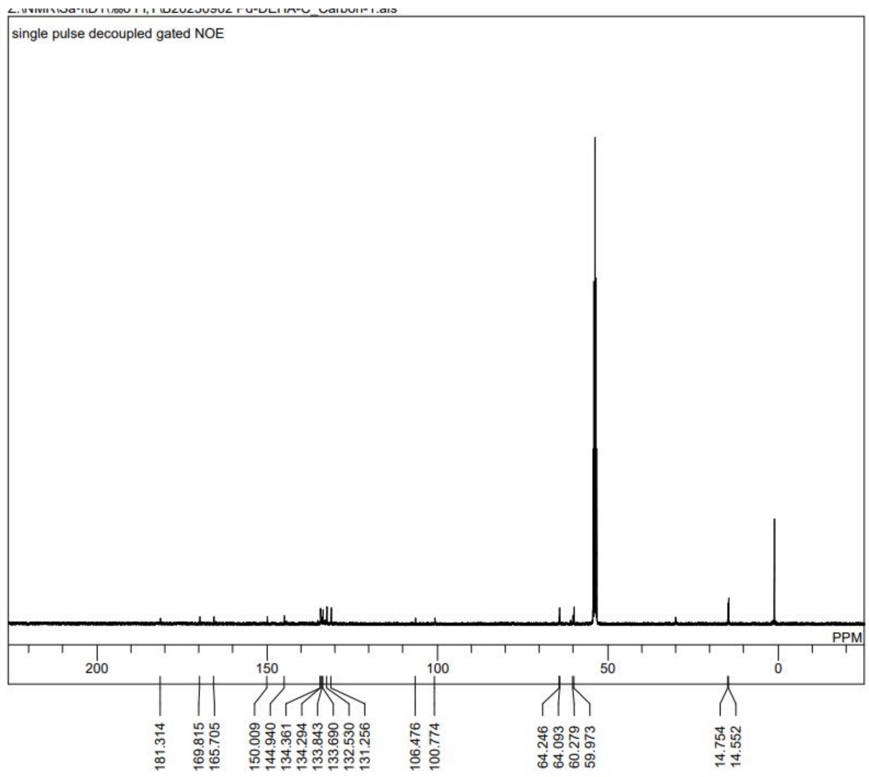

Figure S43 ^{13}C NMR of **Pd-L** in CD_2Cl_2

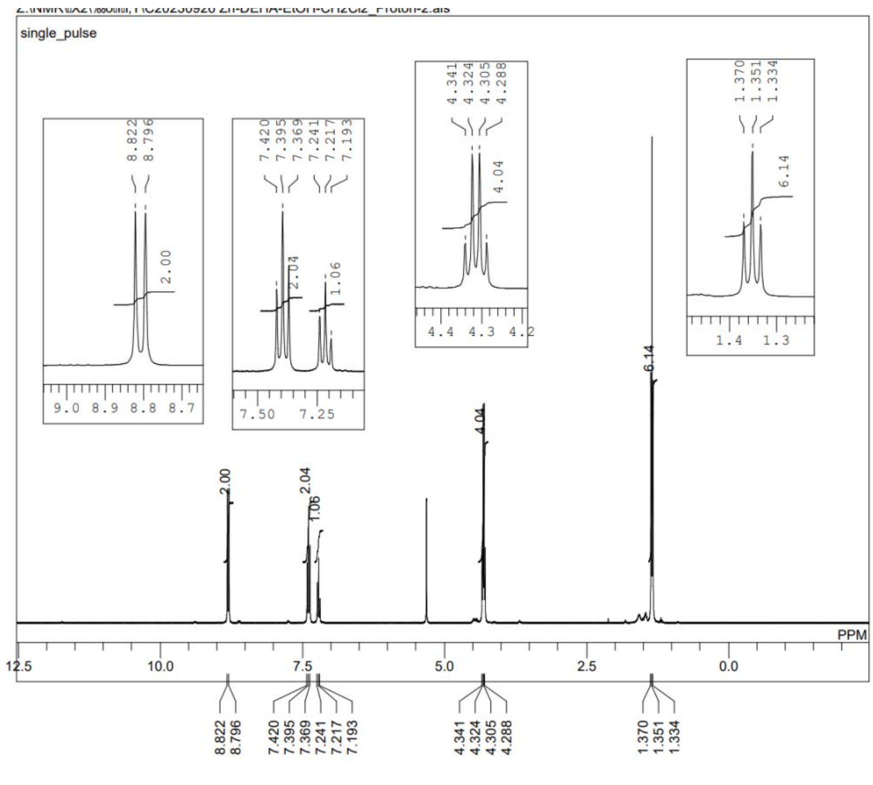

Figure S44 ^{1}H NMR of **Zn-L-Cs** in CD_2Cl_2

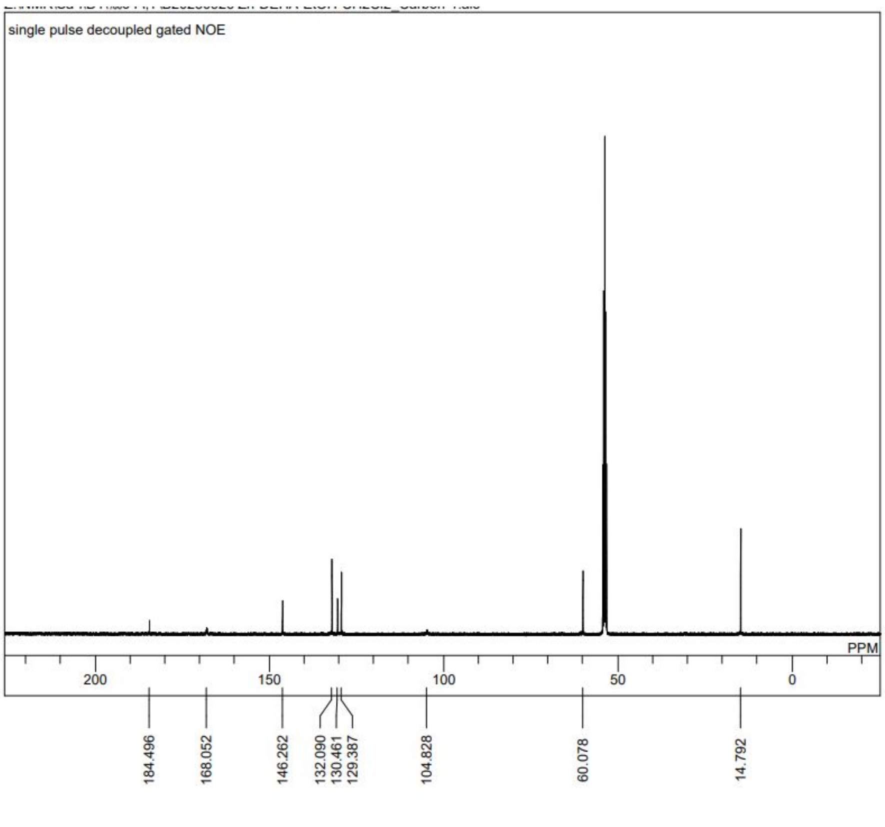

Figure S45 ^{13}C NMR of **Zn-L-Cs** in CD_2Cl_2

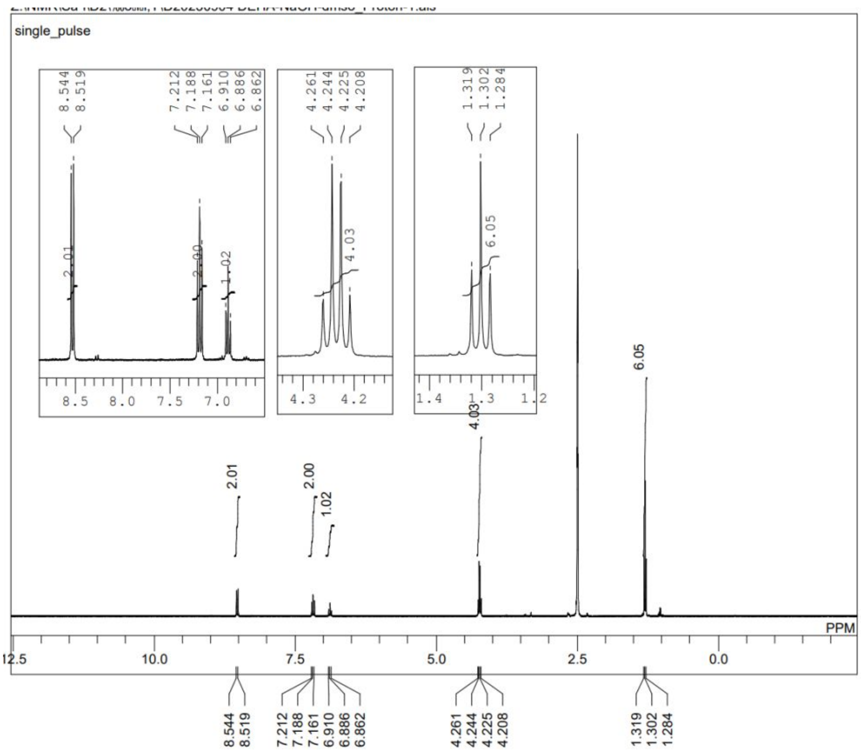

Figure S46 ^{1}H NMR of **Na-L** in DMSO-d_6

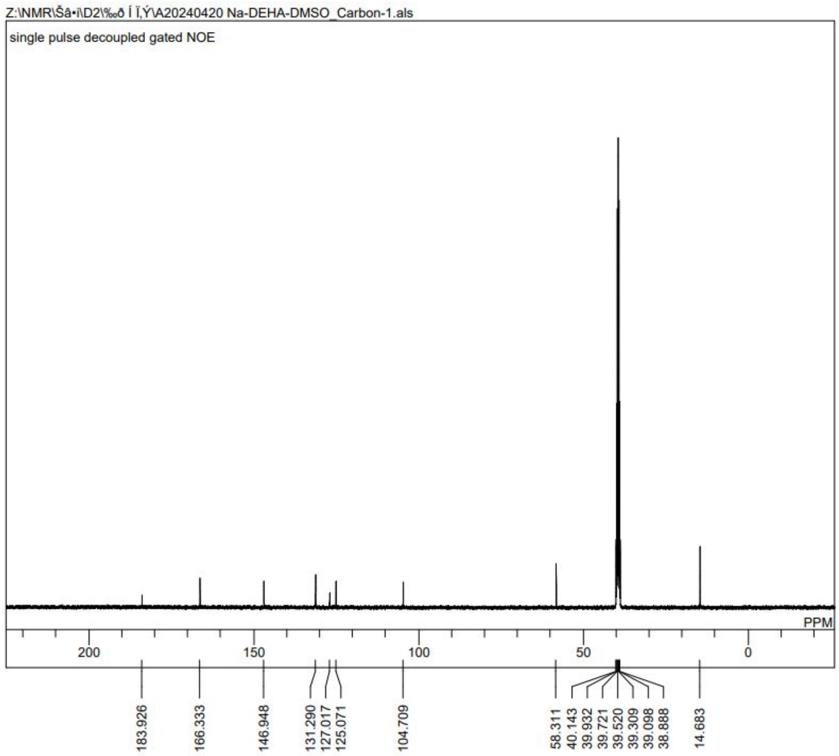

Figure S47 ^{13}C NMR of **Na-L** in DMSO-d_6

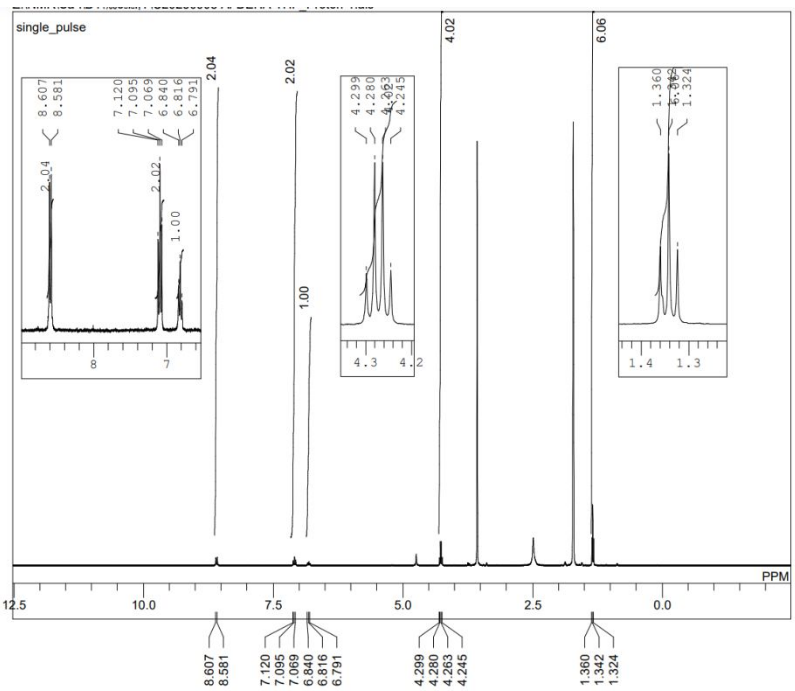

Figure S48 ^{1}H NMR of **Al-L-Cs** in THF-d_8

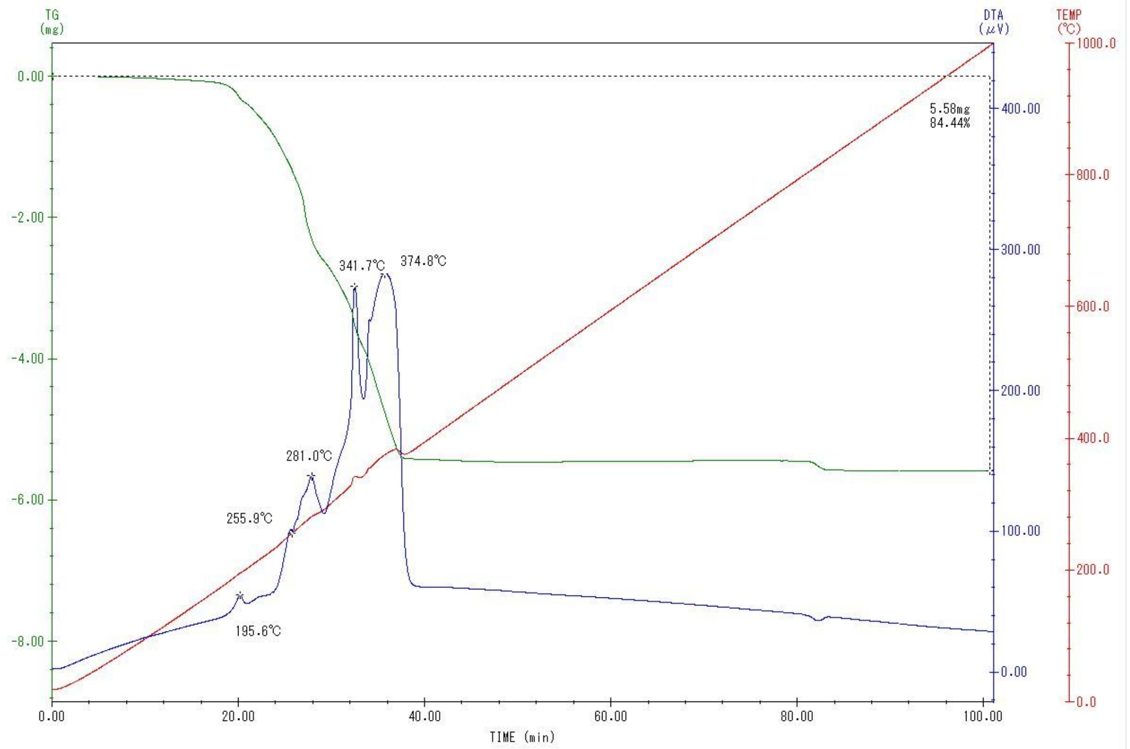

Figure S49 TG-DTA of **Pd-L**

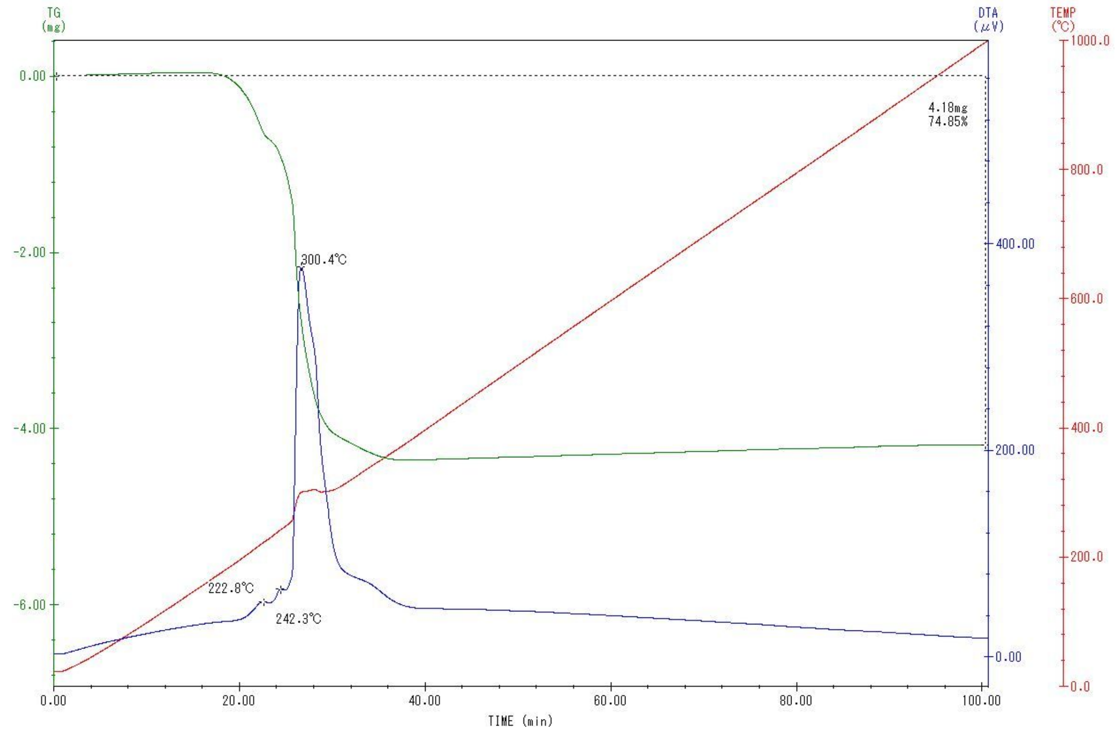

Figure S50 TG-DTA of **Cu-L**

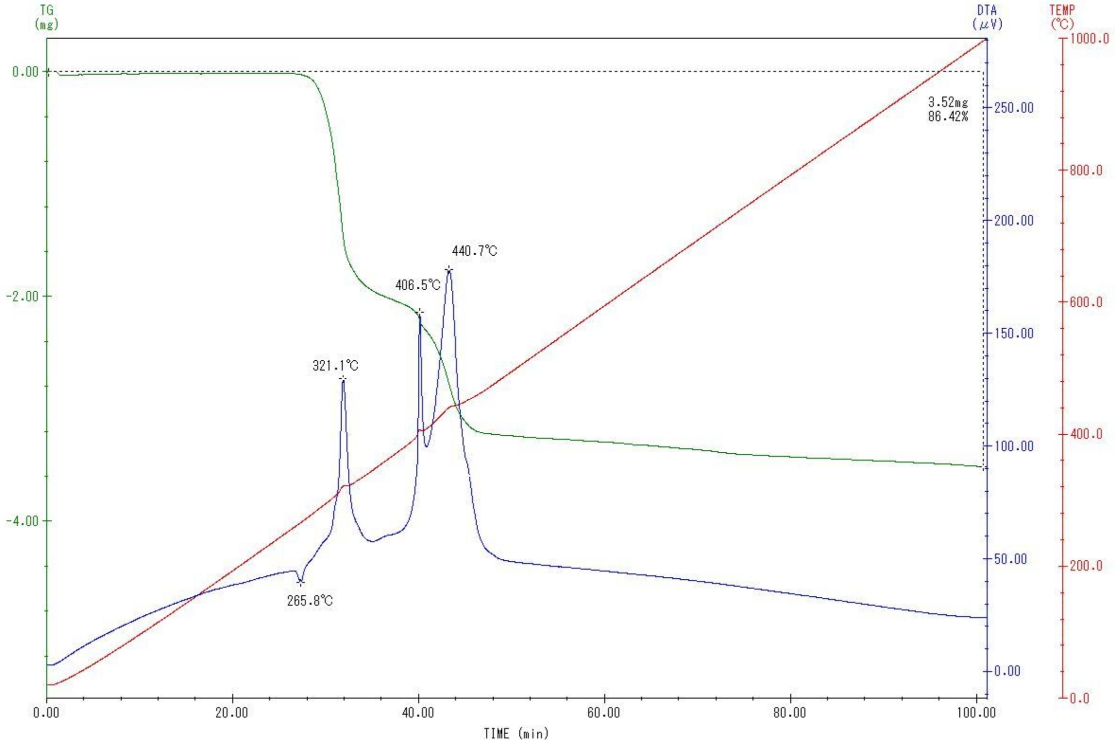

Figure S51 TG-DTA of **Ni-L-Cs**

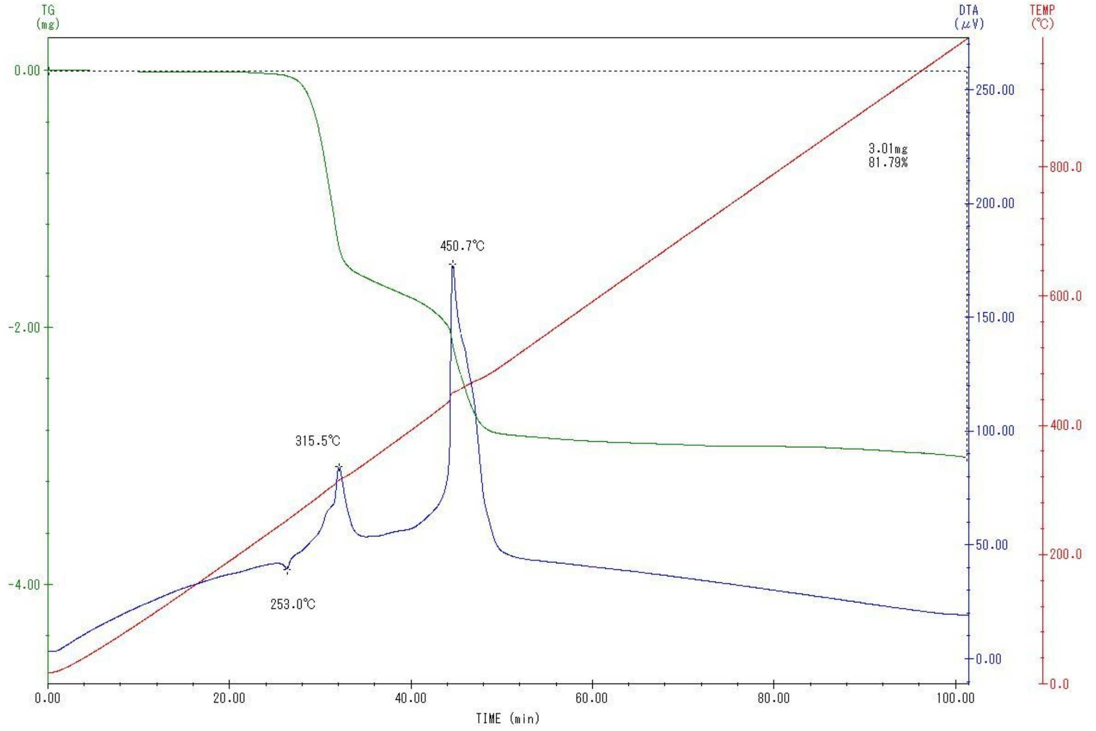

Figure S52 TG-DTA of **Co-L-Cs**

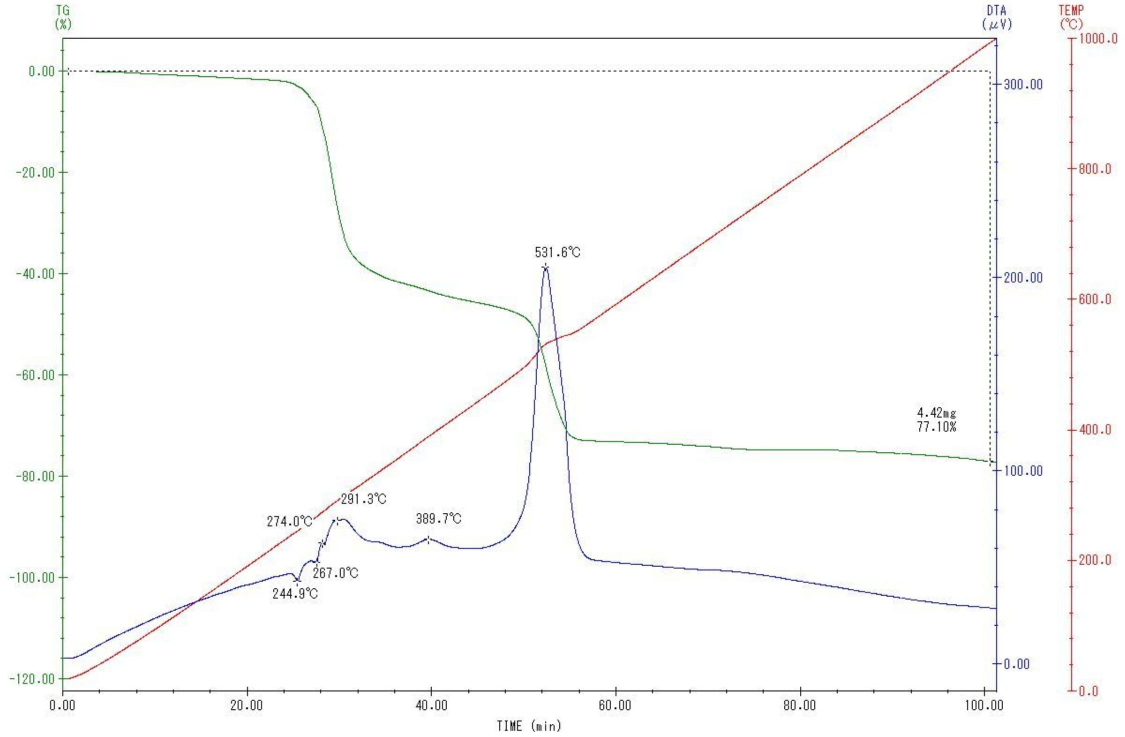

Figure S53 TG-DTA of **Zn-L-Cs**

16. Experimental section

Reagents

Tropolone, thionyl chloride ($SOCl_2$), *p*-toluenesulfonyl chloride, ethyl cyanoacetate, and sodium were purchased from Tokyo Chemical Industry Co. (Tokyo, Japan). Pyridine and iodine were purchased from Kanto Chemical Co., Ltd. (Tokyo, Japan) and purified by distillation. Wakogel@C-200 was purchased from FUJIFILM Wako Pure Chemical Corporation (Tokyo, Japan). Diethyl malonate was purchased from Sigma-Aldrich Inc. (Missouri, USA). Magnesium was purchased from Thermo Scientific Chemicals (Massachusetts, USA). Dehydrated benzene was obtained that commercial benzene was dried by sodium-benzophenone system followed by distillation. Dehydrate ethanol was obtained that commercial ethanol was dried by metallic magnesium and a small amount of iodine followed by distillation.

Synthesis of tropolone chloride (Tp-Cl)

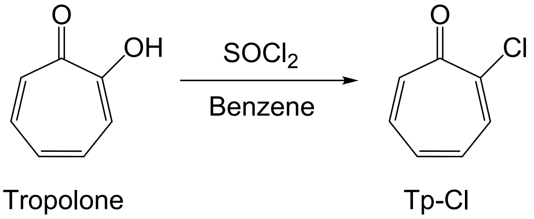

$SOCl_2$ and tropolone were added to dehydrated benzene under argon atmosphere and refluxed for 4 hours. The mixture was then concentrated to give a brown solid. After washing with hexane and concentrating again, a brown solid was obtained. The first fraction was isolated by column

chromatography (eluent: hexane:ethyl acetate=1:2). This was concentrated and dried under reduced pressure to give white needle-like crystals Tp-Cl (yield 93%).

m.p. 59.2–61.2 ℃, ^{1}H NMR (acetone-d_6/ 399 MHz): δ (ppm) 7.09–7.30 (m, 3H), 7.42 (t, J = 10.2 Hz, 1H), 7.98 (d, J = 10.2 Hz, 2H).

Synthesis of diethyl 2-hydroxyazulene-1,3-dicarboxylate (DEHA)

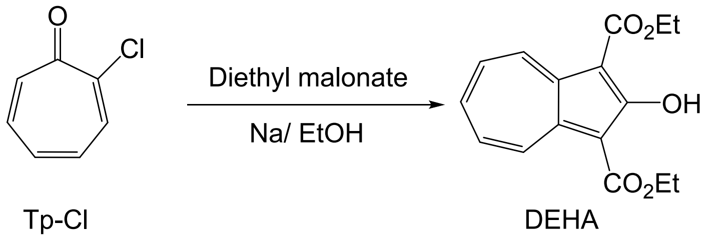

Sodium (0.5 g, 22 mmol) was added to dehydrated ethanol (40 mL) under argon atmosphere to prepare fresh NaOEt solution. Diethyl malonate (1.7 mL, 11 mmol) and Tp-Cl (0.5 g, 3.6 mmol) were added to fresh NaOEt solution, and the mixture was stirred at room temperature for 72 hours. Then 80 mL of water was added and filtered. The filtrate was dissolved in acetic acid, diluted with water, and extracted with chloroform. After concentration, the first fraction was isolated by silica gel column chromatography (ethyl acetate:hexane = 1:3) to afford DEHA as a yellow-orange solid (yield 58%).

m.p. 94.3–95.3 ℃, ^{1}H NMR (acetone-d_6/ 399 MHz): δ (ppm) 1.45 (t, J = 7.2 Hz, 6H), 4.48 (q, J = 7.2 Hz, 4H), 7.88 (t, J = 8.6 Hz, 3H), 9.38 (d, J = 8.6 Hz, 2H), 11.7 (s, 1H).

Synthesis of isolated Na-L by the reaction of DEHA with NaOH in ethanol

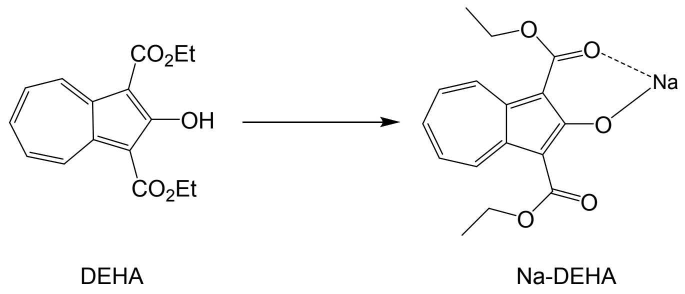

In a dried 20 mL vial, DEHA (0.05 g, 0.17 mmol), ethanol, and water (3:1, 5 mL) were added. In another 20 mL vial, sodium hydroxide (0.15 g, 3.7 mmol), ethanol, and water (3:1, 5 mL) were added. DEHA solution was added to sodium hydrate solution, and yellow precipitate was formed quickly. Solution was stirred for 10 min at 22±5 °C and yellow powder was obtained after filtration (yield 85%).

ESI-HRMS (m/z): calcd. for $[Na(L)+H]^+$ 311.08954; found: 311.08954.

^{1}H NMR (399 MHz, DMSO-d_6): δ (ppm) 1.34 (t, *J*= 7.2 Hz, 6.0 H), 4.30 (q, *J*= 7.2 Hz, 4.0 H), 6.97 (t, *J*= 10.2 Hz, 1.0 H), 7.26 (t, *J*= 10.2 Hz, 2.0 H), 8.59 (d, *J*= 10.2 Hz, 2.0 H).

^{13}C NMR (100 MHz, DMSO-d_6, ppm) 14.7, 58.3, 104.7, 125.1, 127.0, 131.3, 146.9, 166.3, 183.9.

Synthesis of tropolone *p*-toluenesulfonate (Tp-Ts)

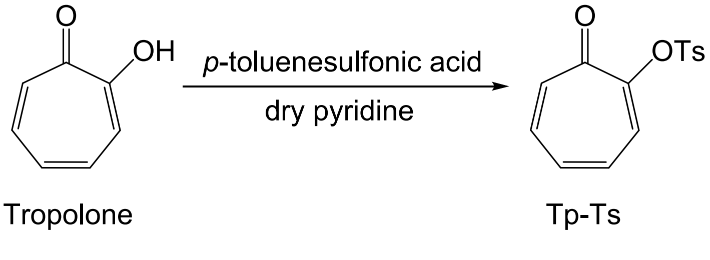

Tropolone (2.08 g, 17.0 mmol), *p*-toluenesulfonic acid chloride (4.08 g, 21 mmol), and dry pyridine (6 mL) were mixed in a dry 50 mL Erlenmeyer flask under argon atmosphere and stirred in an ice bath

for 5 minutes. After stirring at room temperature for 12 h, the mixture was washed well with cold water followed by filtration. The resulting beige solid was dried under reduced pressure and recrystallized with ethanol to give beige needle crystals **Tp-Ts** (yield 90%).

m.p. 157.0–158.1 °C, ^{1}H NMR ($CDCl_3$/ 500 MHz): δ (ppm) 2.45 (s, 3H), 6.98–7.23 (m, 4H), 7.35 (d, *J* = 8.1 Hz, 2H), 7.46 (d, *J* = 8.1 Hz, 1H), 7.93 (d, *J* = 8.1 Hz, 2H).

8.3 Synthesis of diethyl 2-aminoazulene-1,3-dicarboxylate (DEAA)

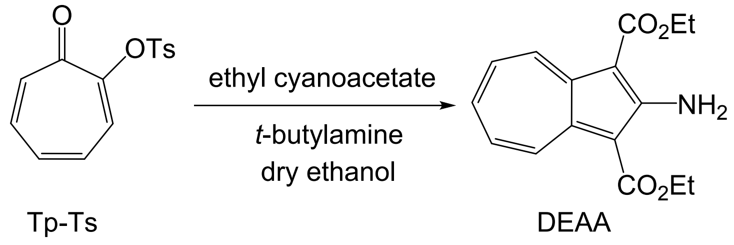

Tp-Ts (4.00 g, 13.9 mmol), dry ethanol (43.5 mL), ethyl cyanoacetate (3.1 mL), and *t*-butylamine (3.1 mL) were added to a dry flask under argon atmosphere and stirred in an ice bath for 10 min. The mixture was stirring at room temperature until **Tp-Ts** dissolved. Then, the mixture was allowed to stand for 12 h. Orange crystal was formed and filtered. Water was added to the filtrate and allowed to stand for 3 h to obtain orange powder followed by filtration. These orange crystal and powder were recrystallized with ethanol and dried under reduced pressure to give ornge crystals **DEAA** (yield 70%).

m.p. 95.5–97.0 °C, ^{1}H NMR ($CDCl_3$/ 500 MHz): δ (ppm) 1.48 (t, *J* = 7.1 Hz, 6H), 4.47 (q, *J* = 7.1 Hz, 4H), 7.44 (t, *J* = 9.6 Hz, 1H), 7.55 (t, *J* = 9.6 Hz, 2H), 7.79 (s, 2H), 9.16 (d, *J* = 9.6 Hz, 2H).

Synthesis of DAAA

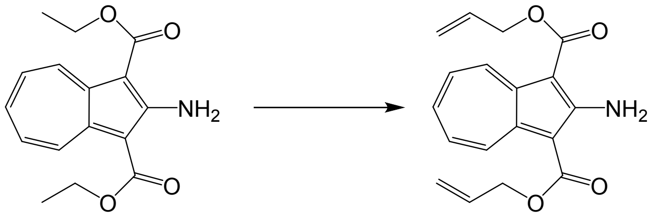

DEAA **DAAA**

In a dried 100 mL recovery flask, DEAA (0.10 g, 0.35 mmol) was dissolved in allyl alcohol (30 mL), and then Ti(O*i*Pr)$_4$ (0.52 mL, 0.18 mmol) was added and dissolved with stirring. Cs_2CO_3 (0.17 g, 0.52 mmol) was then added, and the mixture was stirred at reflux for 1 day. The reaction solution was evaporated, washed with water, and extracted with dichloromethane. After evaporation, the solution was again dissolved in dichloromethane and passed through silica gel. After evaporation, orange viscous liquid **DAAA** was obtained in 90% yield.

^{1}H NMR ($CDCl_3$/ 399 MHz): δ (ppm) 4.93 (d, *J*=5.6 Hz, 4.0H), 5.32 (dd, *J*=10.4, 1.2 Hz, 2.0H), 5.45 (dd, *J*=17.2, 1.2 Hz, 2.0H), 6.08–6.18 (m, 2.0H), 7.45 (t, *J*=9.6 Hz, 1.0H), 7.56 (t, *J*=9.6 Hz, 2.0H), 7.78 (s, 2H), 9.16 (d, *J*=9.4 Hz, 2.0H). ^{13}C NMR ($CDCl_3$/ 100 MHz): δ (ppm) 64.7, 99.6, 118.3, 131.7, 132.9, 133.0, 133.2, 146.5, 162.7, 166.2.

References

(S1) Steyl, G. Bis(tropolonato)palladium(II) *Acta Cryst*. **2005**, *E61*, m1860–m1862. https://doi.org/10.1107/S1600536805026619

(S2) Hamid, M.; Zeller, M.; Hunter, A. D.; Mazhar, M.; Tahir, A. A.; Redetermination of bis(2,4-pentanedionato)palladium(II) *Acta Cryst.*, **2005**, *E61*, m2181–m2183. https://doi.org/10.1107/S1600536805030692
